# Dynamic structural profiling of PINK1 mutations (T313M and L347P) reveals a vital molecular perturbation namely phospho-Serine 65 ubiquitin recognition point in mitophagy mediated autosomal recessive Parkinson’s disease (ARPD)

**DOI:** 10.3389/fnmol.2026.1875988

**Published:** 2026-07-31

**Authors:** Deborah Vincent, C. Sudandiradoss

**Affiliations:** Department of Biotechnology, School of Bio Sciences and Technology, Vellore Institute of Technology, Vellore, Tamil Nadu, India

**Keywords:** autosomal recessive Parkinson’s disease (ARPD), IDR, mitophagy, molecular dynamics, PINK1, PTM, SNP, ubiquitin phosphorylation (Ser65)

## Abstract

**Introduction:**

Autosomal recessive Parkinson’s disease (ARPD) arises from impaired mitophagy due to dysfunction of the PINK1/Parkin pathway, where PINK1-mediated phosphorylation of ubiquitin at Ser65 is essential for pathway activation. However, experimental limitations obscure the effects of disease-associated mutations on intrinsically disordered regions (IDRs) and post-translational modification (PTM) dynamics.

**Methods:**

An integrated computational pipeline was employed to screen 825 PINK1 missense variants, identifying two high-confidence deleterious mutations, T313M and L347P, within the kinase domain. Variant prioritization was complemented by conserved residue, IDR, and PTM analyses, followed by protein–protein docking, molecular dynamics simulations, MM/PBSA binding free-energy calculations, principal component analysis (PCA), and dynamic cross-correlation matrix (DCCM) analysis.

**Results:**

T313M overlapped a conserved phosphorylation site, whereas L347P mapped to conserved active-site residues, with complementary support from IDR analysis. Docking analysis revealed a progressive reduction in binding affinity from the wild type (–88.4 ± 8.2) to L347P (–81.9 ± 3.4) and T313M (–77.4 ± 4.9), accompanied by decreased electrostatic stabilization (–357.6 → –260.6 → –235.5 kcal/mol) and buried surface area (1741.6 → 1624.1 → 1547.4 Å^2^). Molecular dynamics simulations demonstrated that T313M produced the greatest structural and dynamic perturbations, whereas L347P induced moderate destabilization with increased solvent exposure. Although MM/PBSA analysis indicated broadly comparable binding energetics across all systems, PCA and DCCM analyses revealed increased conformational flexibility and altered residue communication in the mutant complexes, particularly T313M.

**Discussion:**

These findings establish a mechanistic link between mutation-induced structural dynamics and impaired PINK1–ubiquitin recognition at Ser65, providing a mutation-specific framework for understanding early mitophagy impairment in ARPD and supporting future molecular assessment and targeted therapeutic development.

## 1 Introduction

Parkinson’s disease (PD) is the second most common neurodegenerative disorder worldwide, characterized by the progressive degeneration of dopaminergic neurons in the substantia nigra pars compacta of the mid-brain resulting in development of cardinal motor symptoms, such as bradykinesia, rigidity, and resting tremor (Chamraj, 2023; Li et al., 2025; Yin and Dieriks, 2025). While the vast majority of patients with PD have a sporadic form of the disease, 8% have an inherited form, with known causative genes at an early onset (Cogan et al., 2025). Among these familial PD patients, autosomal recessive Parkinson disease (ARPD) is a distinct entity, resulting primarily from loss-of-function mutations in PARK6, encoding for the PTEN-induced putative kinase 1 (PINK1) and PARK2, encoding for the Parkin protein (Cogan et al., 2025; Kitada et al., 1998). PINK1 was found to be one of the causative genes for early-onset ARPD, with patients developing the disease at an early-onset, with a similar clinical presentation to that observed for Parkin-associated PD (Olszewska et al., 2022). PINK1 is a serine/threonine kinase that localizes to the mitochondrial outer membrane (MOM), with its kinase domain oriented toward the cytoplasm to facilitate phosphorylation of cytosolic substrates (Zhou et al., 2008). When PINK1 accumulates on the outer mitochondrial membrane, it directly activates Parkin, an E3 ubiquitin ligase, by phosphorylating both Parkin and ubiquitin. This activation triggers Parkin’s translocation to damaged mitochondria. Once recruited, Parkin ubiquitinates multiple outer mitochondrial membrane proteins, marking the organelle for removal. These ubiquitin tags are recognized by autophagy receptors, leading to engulfment of the damaged mitochondrion by autophagosomes and its degradation in lysosomes, a process called mitophagy (Ganley, 2022). In Parkinson’s disease, when PINK1 stabilization and its kinase activity, or Parkin recruitment is impaired, damaged mitochondria are not efficiently cleared. As a result, dysfunctional mitochondria accumulate, leading to reduced ATP production, increased reactive oxygen species (ROS), and cellular stress. In vulnerable neurons (especially dopaminergic neurons), this chronic mitochondrial dysfunction contributes to neuronal injury and progressive neurodegeneration characteristic of Parkinson’s disease (Gladkova et al., 2017; Lazarou et al., 2012; Vives-Bauza et al., 2010). This failure to eliminate depolarized, reactive oxygen species (ROS)-generating mitochondria has been causally implicated in dopaminergic neuronal death in PD, positioning PINK1 stabilization and activation as the pivotal initiating event in neuroprotective mitophagy (Ganley, 2022).

Mitochondrial damage induced PINK1 at the MOM is one of crucial early events in mitophagy, which facilitates phosphorylation of ubiquitin at Serine 65 (Ser65), producing phospho-Serine 65 ubiquitin (Ser65-p-Ub) (Vives-Bauza et al., 2010). Ubiquitin functions as a central signaling modifier in mitochondrial quality control, extending beyond its classical role in proteasomal degradation (Gladkova et al., 2017). PINK1 localizes to the translocase of the outer mitochondrial membrane complex, wherein it dimerizes and undergoes autophosphorylation to achieve activation. Activated PINK1 subsequently phosphorylates ubiquitin conjugated to outer mitochondrial membrane proteins at Ser65. This phosphorylated ubiquitin recruits Parkin, inducing a conformational change that exposes its ubiquitin-like domain (UBL) (Lazarou et al., 2012). PINK1 then phosphorylates the exposed UBL domain at Ser65, thereby fully activating Parkin. Fully activated Parkin ubiquitinates multiple outer mitochondrial membrane substrates, yielding mono-ubiquitin, multi-monoubiquitin, and polyubiquitin chains that include K63- and K48-linked linkages, establishing a feed-forward amplification loop that enhances ubiquitin phosphorylation and drives efficient mitophagy (Lambourne et al., 2023). The resulting phospho-ubiquitin at ser65, serves as a critical intermediate signaling point that recruits and activates E3 ubiquitin ligase Parkin, while PINK1-mediated phosphorylation of the Parkin ubiquitin-like (UBL) domain further enhances its activation (Kane et al., 2014; Kazlauskaite et al., 2014).

Thus, phosphorylation of ubiquitin at Ser65 plays a central role in mitophagy initiation by acting both as a recruitment signal and an allosteric activator of Parkin, thereby promoting mitochondrial quality control in familial forms of Parkinson’s disease such as ARPD (Bayne and Trempe, 2019; McWilliams et al., 2018). As this phosphorylation event is mediated through a direct structural and functional interplay between PINK1 and ubiquitin, understanding the molecular architecture and interaction dynamics of the PINK1–Ubiquitin complex becomes critical to elucidate the mechanistic basis of mitophagy initiation. Minor perturbations in this pathway, particularly those arising from high-risk missense mutations within the PINK1 kinase domain, can disrupt ubiquitin’s critical phosphorylation signaling point and destabilize PINK1–Ubiquitin interaction at a structural level, thereby impairing mitophagy signaling. This deficit in mitochondrial quality control fosters the accumulation of dysfunctional mitochondria, a defining pathological trait in early-onset ARPD. The accumulation of dysfunctional mitochondria consequently promotes excessive production of reactive oxygen species (ROS) (Henrich et al., 2023), impaired ATP generation (Gao et al., 2022), and the release of pro-apoptotic factors such as cytochrome c, ultimately contributing to neuronal degeneration. This phosphorylation cascade, engaging both ubiquitin and PINK1, is vital for mitophagy onset and is frequently disrupted in neurodegenerative disorders like Parkinson’s disease, in which mutations in PINK1 or Parkin compromise mitochondrial clearance.

Beyond structured domains, emerging evidence suggests that intrinsically disordered regions (IDRs) and post-translational modifications (PTMs) play a crucial regulatory role in modulating PINK1 activity, substrate recognition, and protein–protein interactions. However, due to their inherent flexibility, transient conformations, and heterogeneous modification states, these features remain poorly resolved through conventional structural and biochemical approaches, creating a significant gap in understanding their contribution to PINK1–Ubiquitin complex stability and function. This limitation is particularly significant in the context of Parkinsonian neurodegeneration, where dopaminergic neurons within the Substantia Nigra Pars Compacta are especially vulnerable to mitochondrial dysfunction because of their elevated metabolic demand and strong reliance on efficient oxidative phosphorylation. Progressive degeneration of these neurons leads to decline in dopamine levels in the striatum, disrupting basal ganglia circuitry that is essential for coordinated motor control. This dysfunction manifests clinically as core motor symptoms, including bradykinesia and hypokinesia. Notably, these symptoms typically become apparent only after significant loss of dopaminergic neurons, indicating the presence of a prolonged pre-symptomatic phase during which underlying molecular and cellular dysfunction progressively accumulates before overt clinical presentation (Surmeier, 2018; Yang et al., 2006) during which early molecular dysfunction precedes overt neurodegeneration (Janezic et al., 2013). In our study, we have employed a computational framework to model one of the early events in the PINK1–Ubiquitin-mediated mitophagy pathway.

Specifically, PINK1 was computationally modeled in its phosphorylated state at Ser228, alongside ubiquitin phosphorylated at Ser65, with ATP positioned within the kinase binding pocket and Mg^2 +^ incorporated in to mimic a catalytically competent environment for PINK1 activity consistent with preservation of its ability to phosphorylate ubiquitin (Kumar et al., 2017; Okatsu et al., 2018). This configuration enabled structural characterization of the PINK1–Ubiquitin complex under conditions representative of its activated state. Structural insights into PINK1 have been largely derived from insect orthologs such as *Tribolium castaneum* and *Pediculus humanus corporis*, which have enabled detailed characterization of its kinase domain architecture and activation mechanisms (Gan et al., 2024; Woodroof et al., 2011). Extending insights from ortholog-based structural frameworks to the human context, we examined the structural orientation and interaction dynamics of the modeled complex and evaluated the potential impact of high-risk ARPD-associated missense mutations in PINK1 on these interactions, particularly in the context of structural stability and substrate recognition. Furthermore, IDRs and PTM sites were systematically analyzed to investigate how conformational flexibility and regulatory modifications may influence PINK1–Ubiquitin binding dynamics and mutation-induced functional perturbations to provide further context for mutation-induced protein-protein interaction and their structural, functional and conformational variability. In this regard, *in silico* approaches provide a robust and scalable framework to investigate IDR and PTM, whose intrinsic flexibility, transient interactions, and heterogeneous modification states, particularly in kinase proteins limit precise characterization by traditional experimental techniques. Through integrating approaches such as structural modeling of the PINK1 kinase domain alongside dynamic simulations and PTM mapping, these methods enable systematic analysis of structural and functional perturbations that often remain experimentally inaccessible (John and Sudandiradoss, 2025). Rather than replacing experimental approaches, this integrative computational framework provides complementary insights into how structural perturbations including mutation-induced alterations, IDR-mediated flexibility, and PTM-dependent regulation collectively influence PINK1–Ubiquitin interactions and the initiation of mitophagy in ARPD.

## 2 Materials and methods

### 2.1 Baseline structural modeling and stereochemical quality assessment of human PINK1 and ubiquitin retrieval

The three-dimensional structure of human PTEN-induced kinase 1 (PINK1; UniProtKB: Q9BXM7) was retrieved from the AlphaFold Database.^1^ The three-dimensional structure of human PINK1 (UniProt accession Q9BXM7) was generated using the AlphaFold-predicted full-length model as the structural template. The model comprises all 581 amino acid residues (residues 1–581) and includes the N-terminal mitochondrial targeting sequence (residues 1–34), the transmembrane region (residues 94–110), the serine/threonine protein kinase domain (residues 156–511), and the C-terminal regulatory extension (residues 512–581). A deep learning-based structural prediction platform that utilizes advanced neural network architectures to predict protein three-dimensional conformations from amino acid sequences with near-experimental accuracy. By integrating evolutionary and structural information, this framework enables accurate modeling of residue-residue and protein-protein interactions, thereby advancing structural biology and structure-based discovery. The retrieved human PINK1 structure exhibited regions with low predicted Local Distance Difference Test (pLDDT) scores and was therefore subjected to structural refinement and energy minimization prior to downstream analyses. Energy minimization was performed using Swiss-PdbViewer (DeepView),^2^ wherein steric clashes were resolved through geometric optimization. The GROMOS96 force field was employed to relax the structure into a thermodynamically favorable energy state while preserving the overall protein fold.

The ubiquitin structure was retrieved from the RCSB Protein Data Bank (PDB ID:1UBQ),^3^ and non-essential chains were removed prior to refinement. The 4WEP structure, resolved by X-ray crystallography at 1.90 Å resolution, demonstrated high structural precision and reliable atomic positioning, with R-work and R-free values of 0.193 and 0.237, respectively, indicating strong agreement between the structural model and experimental data with minimal overfitting. Additional structural quality metrics further supported model reliability, including 0% Ramachandran outliers, a clashscore of 6, 0.6% side-chain outliers, and 4.1% RSRZ outliers, reflecting only minor local fitting deviations. Similarly, the AlphaFold Predicted Aligned Error (PAE) profile for PINK1 displayed predominantly low error distributions along the diagonal, indicating reliable intra-residue distance prediction and stable secondary structural organization, particularly within the kinase core, whereas elevated inter-domain PAE regions suggested conformational flexibility and positional uncertainty at the terminal regions. The catalytically active conformation of the PINK1 kinase domain was computationally modeled by incorporating phosphorylated serine residues at Ser228 of PINK1 and Ser65 of ubiquitin to represent the activated phospho-state required for substrate interaction. Concurrently, ATP and Mg^2 +^ ions were positioned within the kinase active site based on previously characterized structural orientations to recapitulate the catalytic environment and preserve biologically relevant alignment within the ATP-binding pocket. This configuration enabled accurate representation of the active PINK1 kinase state for subsequent docking and molecular dynamics simulations.

Comprehensive structural validation analyses were subsequently performed to evaluate the stereochemical integrity, residue-environment compatibility, and overall structural reliability of the refined PINK1 model prior to downstream interaction studies and mutant analysis. Ramachandran plot assessment was employed to examine backbone φ (phi) and ψ (psi) torsion angle distributions, enabling classification of residues into favorable, allowed, and disallowed conformational regions as a measure of stereochemical quality. Residue environment compatibility between the three-dimensional structure and the corresponding amino acid sequence was also assessed through environmental profile analysis, while overall structural integrity was evaluated using non-bonded atomic interaction statistics. Collectively, these validation procedures confirmed that the refined protein structures used for protein-protein interaction analyses were stereochemically accurate and structurally reliable.

### 2.2 Ortholog-guided structural modeling of ATP–Mg^2 +^ complex

Protein sequences of PTEN-induced kinase 1 (PINK1) orthologs from human (PINK1_HUMAN),^4^ mouse (PINK1_MOUSE),^5^ and *Tribolium castaneum* (PINK1_TRICA)^6^ were retrieved from UniProt to represent evolutionarily conserved orthologs across species. Multiple sequence alignment was performed using ClustalW^7^ with default parameters to identify conserved regions within the kinase domain, focusing on canonical catalytic motifs such as the HRDLKPEN loop and DFG motif. These conserved features were subsequently used to guide structural modeling of the human PINK1 ATP–Mg^2 +^ complex, which was generated using CHARMM-GUI. ATP and Mg^2 +^ were positioned within the catalytic pocket based on conserved kinase architecture and residue geometry derived from the ortholog alignment. Structural visualization and interaction analysis, including identification of hydrogen bonds and metal coordination, were carried out using PyMOL-plugins through both three-dimensional representations and two-dimensional interaction mapping.

### 2.3 Assessment of missense SNPs in PINK1

The single nucleotide polymorphisms (SNPs) associated with the human PINK1 gene were compiled and analyzed through an extensive computational pipeline. The missense mutations were initially analyzed using the SIFT (Sorting Intolerant From Tolerant) (Sim et al., 2012)^8^ and PolyPhen-2 (Adzhubei et al., 2013) (Polymorphism Phenotyping v2)^9^ tools to predict the potential impact of the mutations on protein’s function. The SIFT method uses a sequence homology-based approach to predict the evolutionarily conserved nature of amino acid residues in the protein sequences and the potential impact of the amino acid substitution based on the probability of the residue being conserved during evolution. The PolyPhen-2 method uses a Naïve Bayes probabilistic classifier that takes into account various sequence and structural features, as well as evolutionarily conserved information, to predict the potential impact of the amino acid substitution. Missense variants were annotated using the Ensembl Variant Effect Predictor (VEP).^10^ Variant identifiers (rsIDs) were submitted in batch mode through custom Python scripts to retrieve gene annotations, amino acid substitutions, transcript consequences, and pathogenicity predictions, including SIFT and PolyPhen-2 scores. As individual variants may be associated with multiple transcript-specific predictions, a transcript-prioritization strategy was implemented. For PolyPhen-2, the highest score reported across all transcripts were retained together with its corresponding qualitative prediction, thereby capturing the most damaging predicted effect for each variant. For SIFT, the lowest score reported across all transcripts was retained along with its associated prediction, as lower SIFT scores indicate a greater likelihood of functional impairment. Variants were subsequently prioritized using a combined pathogenicity-based filtering approach. Functional impact prediction was performed in parallel using SIFT and PolyPhen-2 on the complete set of annotated PINK1 missense variants. Variants were retained only if they met both predefined criteria (SIFT score ≤ 0.05 and PolyPhen-2 score ≥ 0.90), thereby generating a high-confidence set of deleterious variants for subsequent analyses. The filtered variants were then annotated against the ClinVar database (NCB1)^11^ to evaluate their reported clinical significance. Variants classified as Pathogenic, Likely Pathogenic, or Pathogenic/Likely Pathogenic were selected for downstream investigation. To increase confidence in variant interpretation, only variants supported by ClinVar review statuses indicating that assertion criteria had been provided were retained for subsequent structural and functional analyses. These variants were further filtered using tools such as : SNP&GO (Capriotti et al., 2013)^12^ and EVE (Frazer et al., 2021).^13^ SNP&GO uses a Support Vector Machine-based algorithm that combines the information obtained from the sequences of proteins with the Gene Ontology-based information to predict the association of the mutations with diseases.

The EVE tool employs a deep learning model based on the concept of Variational Autoencoders to predict the pathogenicity of the mutations in the sequences of proteins. In order to evaluate the structural implications of the mutations, the curated mutations were analyzed with the Missense3D (Ittisoponpisan et al., 2019)^14^ tool, which uses a structure-based rule system to detect disruptions in the structure caused by the mutations, including steric clashes, charge introduction in the burial, cavity formation, or disruption of secondary structure elements. Furthermore, the impact of the mutations on the stability of the proteins was also analyzed with CUPSAT (Parthiban et al., 2006)^15^ tool CUPSAT is the “Cologne University Protein Stability Analysis Tool.” Based on the above computational investigations, most damaging mutations in terms of function, pathogenicity, structure, and stability were selected for further structural modeling and PPI studies.

### 2.4 Post-translation modification and domain architecture analysis on PINK1

In the post-translational modification analysis of the serine/threonine kinase protein PINK1, experimentally validated databases as well as computational prediction tools were used to identify the complete set of regulation sites. The experimentally validated post-translational modification sites of the protein were collected from the PhosphoSitePlus database (Hornbeck et al., 2015), which contains experimentally identified PTM sites from both high-throughput and low-throughput experiments, as well as from the UniProt database, which contains annotated functional sites as well as kinase-associated modification sites. NetPhos 3.1 (Blom et al., 1999), tool that employs a neural network-based prediction of serine/threonine/tyrosine phosphorylation sites, was used as a prediction tool to identify the experimentally uncharacterized regulation sites of the protein.

### 2.5 Evolutionary conservation analysis and structure-based active site pocket analysis

The analysis of the conserved region of PINK1 was carried out to determine the evolutionarily conserved residues that may be important in the structural integrity and functional activity of the enzyme. The amino acid sequence of PINK1 was analyzed using the multiple sequence alignment tool PRALINE (Simossis and Heringa, 2005), which uses position-specific scoring matrices (PSSMs) and secondary structure prediction for accurate alignment of the sequences. The sequences of PINK1 homologues derived from evolutionarily related species were also included in the analysis to obtain accurate and reliable sequence conservation data. The sequence similarity of the PINK1 residues and motifs was clearly outlined in the alignment. Surface pocket prediction was performed using PrankWeb (Jendele et al., 2019) to identify potential binding-prone regions, which were further evaluated in the context of protein–protein interaction interfaces derived from docking and structural analysis. Here, the experimentally determined protein structure of PINK1, or one at is computationally predicted, is input into Prankweb, and potential binding pockets are ranked according to their probability scores and geometric descriptors. The residues within the top-ranked pockets are targeted as potential active site or binding site residues.

The integration of multiple sequence conservation analysis with the structure-based active site prediction enabled the identification of key residues that were both evolutionarily and functionally relevant. This approach enabled the development of a robust computational framework to define the key functional determinants of the PINK1 protein.

### 2.6 Comparative protein-protein docking analysis of interactions between PINK1 and ubiquitin

Protein–protein docking studies were performed to investigate the interactions between the modeled phosphorylated PINK1–ubiquitin (pSer65-Ub) complexes of both wild-type and mutant PINK1 structures. Protein–protein docking was performed using the HADDOCK version 2.4 (High Ambiguity Driven biomolecular DOCKing) with default docking parameters unless otherwise specified. (Honorato et al., 2024). Ambiguous Interaction Restraints (AIRs) were defined based on the predicted functional and mutation-associated residues of PINK1 and the corresponding ubiquitin interface residues. HADDOCK employed its standard three-stage protocol comprising rigid-body energy minimization (it0), semi-flexible simulated annealing refinement (it1), and explicit water refinement. Docked models were ranked using the default HADDOCK scoring function, which combines van der Waals, electrostatic, desolvation, restraint violation, and buried surface area terms. Default clustering based on the fraction of common contacts (FCC) was used, and no non-default restraints or scoring parameters were applied. The top-ranked cluster, determined by the most negative HADDOCK score and Z-score, was selected for subsequent structural analyses and molecular dynamics simulations. The resulting docking conformations were clustered according to interface root mean square deviation (i-RMSD), and clusters were ranked based on docking scores derived from a weighted combination of van der Waals energy, electrostatic energy, desolvation energy, and restraint violation energy terms. The top-ranked complexes were selected for downstream interaction analysis, and their binding interfaces were evaluated to characterize key intermolecular interactions contributing to complex stability. Visualization of the intermolecular interactions of the docked complexes was performed by using LigPlot+ (Laskowski and Swindells, 2011), which produced graphical representations of the hydrogen bonds and hydrophobic interactions at the protein–protein interfaces in two-dimensional form, thus allowing an in-depth characterization of the key residue-based interactions that are crucial for the stability of the complexes.

### 2.7 Molecular dynamics–based evaluation of interaction-associated conformational stability

Molecular dynamics simulations of protein–protein complexes were conducted using GROMACS version 2025.4 (Huang and MacKerell, 2013) employed as the MD engine. Protein topologies were generated employing the CHARMM36 force field, while ATP and Mg ions were parameterized via SwissParam (Bugnon et al., 2023). ATP and Mg were incorporated to emulate a catalytically competent kinase environment, wherein Mg stabilizes the ATP–kinase complex by neutralizing negative charges on the ATP phosphate groups, facilitates optimal coordination geometry essential for phosphoryl transfer, and buttresses pivotal structural features, such as PINK1’s DFG motif and activation loop, which are crucial for kinase conformation and substrate binding. The protein complexes were placed in a dodecahedral simulation box with a minimum distance of 1.0 nm between the protein surface and the box boundary and solvated with explicit water molecules. Na+ and Cl- ions were added to neutralize the system. Standard protonation states corresponding to physiological pH (∼7.0) were assigned during system preparation. Energy minimization was performed using the steepest descent algorithm, followed by 100 ps NVT equilibration at 300 K using the V-rescale thermostat and 100 ps NPT equilibration at 1 bar using the Parrinello–Rahman barostat. Production molecular dynamics simulations were subsequently carried out for 200 ns with coordinates recorded every 20 ps. To ensure reproducibility and statistical robustness, three independent simulation replicates were performed for each system using different initial velocity distributions.

The post-Simulation trajectory analysis was used to study the structural stability of the protein complexes. Root Mean Square Deviation (RMSD) was used to calculate the deviation in the structure of the backbone atoms of the protein. Root Mean Square Fluctuation (RMSF) was used to calculate the flexibility of the residues in the structure of the protein, represented in nm. The radius of gyration (Rg) was also calculated to measure the compactness of the protein structure during the simulation and was reported in nm. The solvent accessible surface area (SASA) was also calculated using the rolling probe method, which calculates the accessible surface area of the protein to solvent molecules, and was reported in nm^2^. Furthermore, hydrogen bonds were also analyzed based on geometric criteria, such as distances between the donor and acceptor of ≤ 0.35 nm and hydrogen-bonded angles of ≥ 120°, to understand the hydrogen bonding patterns during the simulation.

#### 2.7.1 Molecular mechanics Poisson-Boltzmann surface area (MM/PBSA) analysis of binding energetics

To complement the dynamic insights from principal component analysis and dynamic cross-correlation matrix analyses, the binding energetics of PINK1–ubiquitin complexes were evaluated using the MM/PBSA approach. This method provides a quantitative assessment of protein–protein interactions by decomposing the total binding free energy into distinct energetic contributions, offering a thermodynamic perspective on interaction stability and specificity. Calculations were conducted on equilibrated trajectories from the production phase of molecular dynamics simulations, with representative snapshots sampled at regular intervals to ensure comprehensive conformational coverage. The total free energy was partitioned into molecular mechanics and solvation components. The molecular mechanics terms included bonded contributions (bonds, angles, improper dihedrals, correction maps, Urey–Bradley, and the non-bonded interactions) that describe the system’s internal structural integrity, alongside non-bonded van der Waals and electrostatic energies the key drivers of protein–protein interface stability via packing and electrostatic complementarity. Solvation energy was further divided into polar and non-polar components to account for desolvation effects and hydrophobic contributions at the interface. The gas-phase energy and solvation energy were then combined to yield the overall binding free energy, enabling evaluation of the balance between intermolecular interactions and solvent effects. This detailed energetic decomposition characterizes the forces governing PINK1–Ub interactions, positioning MM-PBSA as an ideal method for assessing mutation-induced changes in interface stability and energetics alongside structural and dynamic analyses. Binding free energy calculations were conducted using gmx_MMPBSA on the final 1,000 frames of each trajectory, and the reported values represent the mean ± standard deviation across the three replicates.

### 2.8 Conformational landscape analysis using principal component analysis (PCA) derived free energy landscape and dynamic cross-correlation matrix (DCCM)

PCA was performed on the production MD trajectories of PINK1–Ub ubiquitin complexes to evaluate collective conformational dynamics. Trajectories were pre-processed by removing solvent and ions, followed by least-squares fitting using Cα atoms to eliminate translational and rotational motions. The covariance matrix of Cα atomic fluctuations was constructed and diagonalized using the GROMACS tools *gmx covar* and *gmx anaeig*, where *gmx covar* was employed to generate and diagonalize the covariance matrix, and *gmx anaeig* was utilized to analyze the resulting eigenvectors and eigenvalues. The first two principal components (PC1 and PC2) were used to generate a two-dimensional free energy landscape for identifying dominant conformational states. The same covariance matrix obtained from *gmx covar* was further utilized to compute residue-wise cross-correlation coefficients for DCCM analysis. The resulting correlation matrices were visualized as heatmaps to identify correlated and anti-correlated motions within the complexes. Correlation coefficients ranged from −1 to +1, where +1 indicated fully correlated motion, −1 indicates fully anti-correlated motion, and 0 indicates no correlation between residue pairs, these parameters were further considered for DCCM analysis. Comparative analysis between unmodified and phosphorylated systems was performed to assess changes in residue communication and long-range dynamic interactions induced by Ser65 phosphorylation.

## 3 Results and discussion

### 3.1 Model optimization and system preparation for PINK1-Ubiquitin complex

The modeled PINK1 structure exhibited regions with low pLDDT confidence scores, signifying diminished reliability in specific local structural features. To enhance structural fidelity for downstream analyses, the model underwent refinement via the SWISS-MODEL homology modeling pipeline, which improved backbone geometry and side-chain conformations, thereby increasing its appropriateness for docking and molecular dynamics simulations. Further, this refinement involved assessing global structural assessment through SAVES 6.0 scores and Ramachandran plots to confirm the dihedral angles of residues were within acceptable parameters. The ubiquitin structure (PDB ID:1UBQ), originally obtained as a multichain assembly, was represented as a single monomeric chain, consistent with the functional role of ubiquitin in PINK1-mediated phosphorylation. To represent the biologically relevant phosphorylated state, the Ser65 residue of ubiquitin was computationally modeled as phosphoserine (SEP). In addition, phosphorylation at Ser228 of PINK1 was computationally modeled to reflect its activated state. These residue-level changes enabled the construction of a physiologically relevant PINK1–Ub (Ser65-p-Ub) complex, ensuring that subsequent docking and simulation analyses accurately capture phosphorylation-dependent interaction dynamics. Likewise, the refined PINK1 model exhibited satisfactory stereochemical quality and overall structural stability upon validation. Analysis of the Ramachandran plot indicated that 86.4% of residues reside in the most favored regions, with 12.1% in allowed regions, reflecting superior overall backbone stereochemistry. Merely 0.8% of residues occupied disallowed regions, such that over 98% are positioned in favored or allowed areas: A profile indicative of a robust structural model with negligible steric strain. Side-chain evaluation revealed five residues with atypical χ1–χ2 rotamer conformations; side-chain parameters placed four in favorable regions and one in a less favorable region, suggesting only localized deviations. Backbone geometry was largely consistent (maximum residue deviation of 19.7; bond length/angle deviation of 4.5), with no bad contacts or steric clashes. However, one cis peptide bond and five D-amino acids are unusual and warrant verification to exclude modeling artifacts or context-specific features. The Z-score analysis showed moderate deviations in Ramachandran plot appearance alongside a suboptimal backbone conformation score, suggesting possible localized strain amid generally acceptable stereochemical parameters (Supplementary Figure 1). Subsequently, RMS Z-scores for bond lengths, bond angles, and side-chain planarity remained within acceptable limits. Additionally, the overall G-factor of 0.36 indicated good stereochemistry, while the ERRAT quality factor of 94.309 confirmed reliable non-bonded interactions and a robust global structure. Residue-wise validation revealed most residues within acceptable error thresholds, exhibiting only minor sequence-wide fluctuations. Overall, these metrics affirm good model quality and stereochemical reliability, although refinement of localized backbone deviations could be beneficial.

### 3.2 Ortholog-guided conservation and structural characterization of the PINK1 catalytic pocket

**FIGURE 1:**
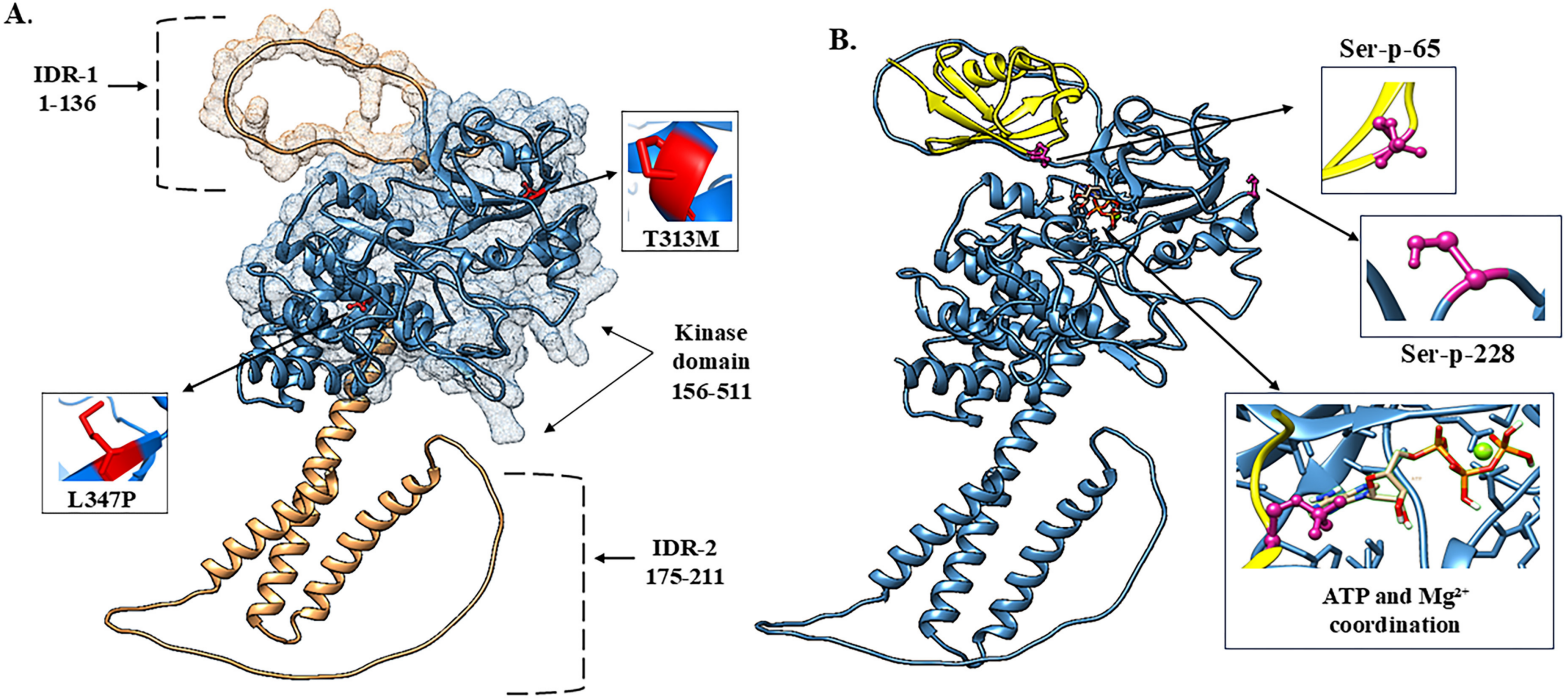
Structural representation of the wild-type PINK1–ubiquitin complex. The PINK1 kinase domain is shown in cartoon representation, while Intrinsically Disordered Regions (IDRs) are depicted as a transparent surface. Ubiquitin is bound at the substrate recognition interface. **(A)** Overall complex highlighting domain organization, IDRs, kinase domain, and mutation sites (T313 and L347). **(B)** Close-up view of the active site showing Mg^2 +^ coordination and phosphorylated residues, including ubiquitin Ser65 (Ser_p65) and the autophosphorylation site Ser228 (Ser_p228) in human PINK1, emphasizing their roles in catalytic activity and substrate recognition.

**FIGURE 2:**
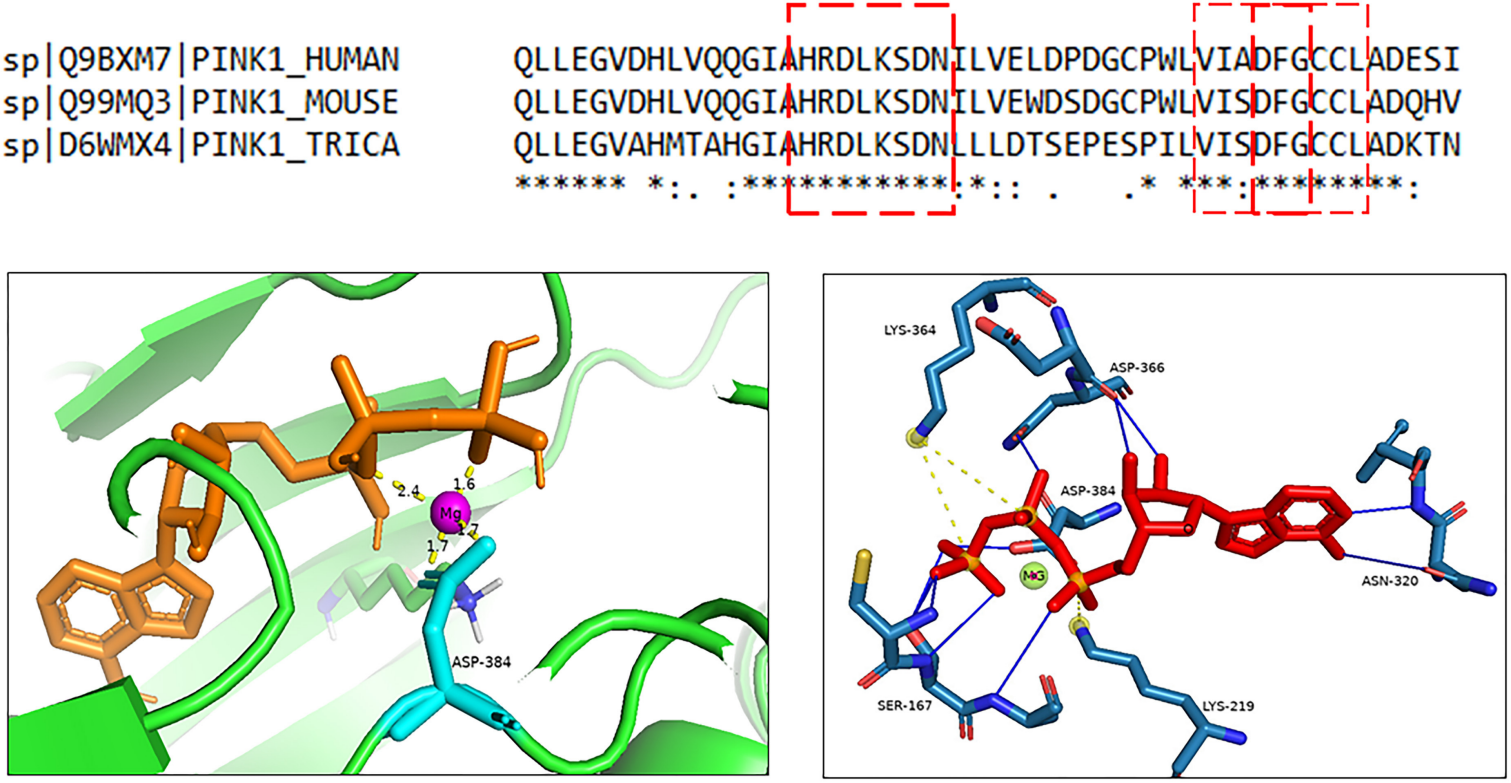
Domain architecture and active-site features of the PINK1–ubiquitin complex. Ortholog-specific domains are highlighted and mapped onto the structure to illustrate conserved and divergent regions. The left inset shows the Mg^2 +^ coordination environment along with bound ATP within the catalytic site. The right inset presents a 2D interaction diagram depicting Mg^2 +^ coordination and ATP-binding interactions, emphasizing key residues involved in catalysis and nucleotide stabilization.

Comparative sequence analysis of PINK1 orthologs from human (PINK1_HUMAN), mouse (PINK1_MOUSE), and *Tribolium castaneum* (PINK1_TRICA) revealed strong evolutionary conservation within the kinase domain (Figures 1B, 2), particularly across key catalytic motifs. The HRDLKPEN catalytic loop and DFG motif were strictly conserved, with residues Asp366 and Asp384 invariant across all species, consistent with their established roles in catalysis and Mg^2 +^ coordination. Integration of sequence conservation with structural modeling indicates that ATP is accommodated within a canonical kinase binding pocket, where its phosphate groups are positioned in proximity to Lys364 and Asp366, supporting stabilization within the catalytic environment. The hinge-region residue Val241 contributes to adenine binding, while Gly386 provides local conformational flexibility that may facilitate phosphate accommodation (Gan et al., 2022; Kumar et al., 2017). Although Lys219 was observed to interact with ATP, its lower conservation across orthologs and peripheral positioning suggest that it is unlikely to serve as a primary anchoring residue. The Mg^2 +^ ion is positioned within the catalytic pocket in a geometry consistent with active kinase conformations, with primary coordination mediated by Asp384 of the DFG motif at a distance of ∼2.0–2.3 Å. Asp366 further contributes to the catalytic environment, supporting proper orientation of ATP phosphates, while residues such as Asn320 and Ser167 may provide indirect stabilization, including water-mediated interactions, although their precise roles cannot be conclusively determined from the present model. Overall, mapping sequence conservation onto the structural model highlights a clear distinction between conserved catalytic residues and less conserved peripheral residues; the former localizes to the ATP-binding and Mg^2 +^ coordination sites and underpin catalytic function, whereas the latter may contribute to local structural or electrostatic stabilization without direct involvement in catalysis. Collectively, the concordance between conserved sequence features and the modeled structural arrangement supports the structural plausibility of the ATP–Mg^2 +^ binding configuration in human PINK1. These findings have been pictorially elaborated in Figures 1B, 2, respectively. However, our results provide a strong foundation for future experimental validation of PINK1-mediated ubiquitin recognition and advance an overall understanding of the molecular basis of early-onset autosomal recessive Parkinson’s disease and will facilitate the development of mechanism-based therapeutic strategies targeting PINK1-mediated mitophagy.

### 3.3 Multi-tool pathogenicity and stability assessment of PINK1 mutations

A total of 825 missense mutations in Human PINK1 (PTEN-induced kinase 1; UniProt: Q9BXM7). A total of 825 missense variants were collected and subjected to functional annotation using the Ensembl Variant Effect Predictor (VEP). Variant identifiers (rsIDs) were submitted in batch mode to the Ensembl VEP database, which was used to retrieve variant consequences, affected genes, transcript annotations, amino acid substitutions, and *in silico* pathogenicity predictions. The returned annotations were parsed and processed using a custom Python script. For variants associated with multiple transcript-specific predictions, a conservative prioritization strategy was adopted. PolyPhen-2 scores were aggregated by selecting the maximum score reported across all transcripts for each variant, thereby retaining the most damaging predicted effect. The corresponding PolyPhen-2 qualitative prediction (benign, possibly damaging, or probably damaging) associated with the maximum score was also retained. Similarly, SIFT scores were aggregated by selecting the minimum score reported across all transcripts for each variant, as lower SIFT scores indicate a higher probability of deleterious functional effects. The corresponding SIFT prediction was retained for downstream analyses. Variants were subsequently filtered to retain those predicted as deleterious by SIFT (score ≤ 0.05) and exhibiting high PolyPhen-2 scores ( ≥ 0.90), indicative of potentially damaging amino acid substitutions. This filtering step reduced the initial set of 825 missense variants to 276 high-confidence deleterious variants. To assess clinical relevance, the filtered variants were annotated using the ClinVar database. Clinical significance classifications were then examined, and variants annotated as Pathogenic, Likely Pathogenic, or Pathogenic/Likely Pathogenic were retained. Among these, 203 variants had corresponding ClinVar records, of which 20 were classified as pathogenic, likely pathogenic, or pathogenic/likely pathogenic. Finally, variants were further prioritized based on ClinVar review status, retaining only those supported by review categories indicating “criteria provided” (single submitter or multiple submitters with no conflicts) (Table 1). This systematic filtering strategy resulted in five high-confidence clinically relevant variants: A168P (rs768091663), T313M (rs74315359), L347P (rs28940285), F385L (rs763416852), and C388R (rs1553146806), which were selected for subsequent structural and molecular dynamics analyses. These 5 variants were therefore filtered as the final high-confidence candidates and subjected to detailed multi-tool pathogenicity characterization encompassing evolutionary (EVE), disease-association (SNP&GO), structural (Missense3D), and thermodynamic (CUPSAT) prediction approaches. Thermodynamic stability analysis further revealed that most variants destabilized the protein structure, with L347P exhibiting the strongest destabilizing effect (ΔΔG = −7.23 kcal/mol), while T313M displayed moderate yet second strongest destabilizing mutation (ΔΔG = −2.79 kcal/mol). Importantly, both T313M and L347P showed consistent deleterious predictions across functional, evolutionary, structural, and thermodynamic analyses. Based on the collective evidence obtained through SIFT, PolyPhen-2, EVE, structural assessment, and thermodynamic stability evaluation, T313M (rs74315359) and L347P (rs28940285) were prioritized for subsequent structural and interaction analyses. These variants were further examined to determine their potential overlap with PTM sites and kinase domain region, owing to their predicted impact on structural stability and functional regulation within the PINK1 kinase domain in Figure 1A. The stepwise filtering process of the variants have been represented in Supplementary Figure 1 and Supplementary Table 1. While, the total collection of 825 missense variants have been reported in Supplementary Table 2, respectively.

**TABLE 1:** Computational prediction and structural impact analysis of missense variants using multi-tool assessment.

rs ID	A.A substitution	SIFT		Polyphen v-2		EVE		SNP & GO		Missense 3D		CUPSAT	
		Prediction in values	Score	HumVar prediction	Score	Prediction in values	Score	Effect	RI	Prediction	Residue type	ΔΔ G kcal/mol	Stability
rs768091663	A168P	Affect	0.00	2.00	1.00	2.00	0.68	Disease	4	Buried	Damaging	2.39	Stabilizing
rs74315359	T313M	Affect	0.00	2.00	1.00	2.00	0.78	Neutral	0	Buried	Damaging	−2.79	Destabilizing
rs28940285	L347P	Affect	0.00	2.00	1.00	2.00	0.77	Disease	3	Buried	Damaging	−7.23	Destabilizing
rs763416852	F385L	Affect	0.00	2.00	1.00	2.00	0.72	Disease	2	Buried	Benign	−0.65	Destabilizing
rs1553146806	C388R	Affect	0.00	2.00	1.00	2.00	0.74	Disease	3	Buried	Damaging	2.19	Stabilizing

Functional effects of amino acid substitutions were evaluated using SIFT, PolyPhen-2 (HumVar), EVE, SNP&GO, Missense3D, and CUPSAT. Qualitative predictions from PolyPhen-2 (HumVar), EVE, and Missense3D residue exposure were converted into numerical values to enable standardized comparison (HumVar: 0 = benign, 1 = possibly damaging, 2 = probably damaging; EVE: 0 = benign, 1 = uncertain, 2 = pathogenic; residue type: 0 = exposed, 1 = buried). SIFT and SNP&GO scores are presented alongside their respective prediction values. Structural effects were further assessed using Missense3D and stability changes (ΔΔG, kcal/mol) predicted by CUPSAT, where positive values indicate stabilizing and negative values indicate destabilizing mutations.

### 3.4 Structural and regulatory landscape of PINK1 coupling intrinsically disorder regions and post-translational modification organization

We analyzed the conformational flexibility and binding-prone regions within PINK1 and identified two major intrinsically disordered regions along with a functionally important linker/transition region in the PINK1 protein sequence, as presented in Supplementary Tables 1, 3. The IDR-1, which spans from 1 to 136, includes the N-terminal mitochondrial targeting sequence. The amino acids present in the IDR-1 region have IUPred scores higher than the threshold of 0.5, with representative scores of R42 (0.5419), G43 (0.5419), P52 (0.5665), and K135 (0.5176). These scores confirm the presence of intrinsic disorder. The ANCHOR scores of the IDR-1 region were below 0.5, indicating the presence of disordered regions but the absence of binding-prone character. The boundary regions of IDR-1, S136 (IUPred: 0.6375, ANCHOR: 0.52) and K137 (IUPred: 0.6227, ANCHOR: 0.54), transitioned from disordered region additionally exhibited a transition towards a binding-prone state, initiating the linker or transition region from 136 to 146. The disordered character of the IDR-1 regions shifts into the linker or transition region, where the ANCHOR scores of the regions show increasing character, peaking at R146 (ANCHOR: 0.68). These regions have the ability to undergo disorder-to-order transitions when they interact with binding partners, thus acting as molecular recognition features.

The most functionally significant disordered region was found to be IDR-2, which corresponds to residues 175–211, as this region was found to possess the highest levels of disorder and binding propensity in the entire PINK1 sequence. IUPred scores for IDR-2 were found to rise dramatically from residue L182 (0.5211) to peak at residue G202 (0.9381), where it recorded the highest score for disorder in the entire sequence. Similarly, ANCHOR scores for IDR-2 rose in parallel from residue L182 (0.60) to peak at residue G202 (0.95), where it recorded the highest score for disorder and MoRF propensity in the entire sequence. This consistently elevated scores across the entire IDR-2 region, with 28 out of 30 residues exhibiting scores above 0.70, indicate strong binding propensity and a high likelihood of undergoing disorder-to-order transitions during protein–protein interactions. We further characterized the structural and functional organization of human PINK1, Pfam domain mapping was integrated with IUPred-based intrinsic disorder predictions. The domain analysis delineated thirteen distinct functional regions spanning the full-length protein (Supplementary Table 2). The N-terminal region (residues R10–G24) encodes a mitochondrial targeting sequence with an amphipathic helix responsible for mitochondrial import and membrane targeting, followed by a transmembrane-proximal hydrophobic segment (L45–L56) mediating membrane association. A pre-kinase regulatory linker (R90–F98) provides structural stabilization preceding the kinase domain. The central protein kinase domain encompassed several functionally critical motifs: the glycine-rich ATP-binding loop (GxGxxG; G309–K316), which coordinates ATP phosphate binding and harbors the pathogenic T313 residue; the VAIK catalytic motif (V327–K330) for ATP positioning; and the hinge region (V340–P349), which stabilizes the catalytic pocket and contains the pathogenic L347 residue (La Sala et al., 2016; Strong et al., 2011). The catalytic loop HRDLKPEN motif (H357–N363) governs phospho-transfer, while the DFG motif (D384–G386) coordinates Mg^2 +^ for kinase activation. A substrate-positioning activation segment (T386–D397) and a ubiquitin recognition surface (E417–K423) underscore the direct structural basis for Phospho-Ubiquitin binding.

Complementing these domain annotations, disorder analysis revealed that the N-terminal targeting sequence, the pre-kinase regulatory linker, and the post-kinase C-terminal regulatory region (D525–W529) exhibit elevated disorder scores, consistent with their roles as flexible regulatory and interaction-permissive segments. The extreme C-terminal tail (R560–S571) similarly showed partial disorder, implicating it in protein stability regulation. Notably, the core kinase domain residues, including those surrounding the pathogenic T313M and L347P mutation sites, retained low disorder scores, confirming their structural rigidity within the folded kinase domain. Collectively, the integration of Pfam domain mapping and IUPred disorder prediction highlights that PINK1 employs a modular architecture wherein a structured catalytic core is flanked by intrinsically disordered regulatory regions, and that the T313M and L347P mutations reside within highly conserved, ordered kinase motifs critical for ATP binding and catalytic pocket integrity (Supplementary Table 3).

MetaPredict evaluation revealed that IDR-2 residues were consistently predicted as disordered and binding prone. Beyond residue 211, the PINK1 sequence enters an ordered region (212–581) that includes the kinase domain, although only a few residues, S261 (IUPred: 0.5055), K262 (0.5419), R263 (0.5382), G264 (0.5419), and P416 (0.5254), have slightly higher disorder propensity and are classified as local or isolated disorder within the ordered kinase domain. Most importantly, the prioritized mutations T313M and L347P are located in the ordered kinase domain region (212–581) outside the IDR boundaries. However, their proximity to the IDR-2 region (175–211) is significant, as this latter region is characterized by peak binding prone propensity at G202 (ANCHOR: 0.95). Because T313M and L347P are located within the ordered kinase domain and outside the identified IDRs, any functional communication between these mutations and IDR-2 remains speculative. Further structural and biochemical studies are required to determine whether these mutations influence the dynamics or interaction propensity of IDR-2. Moreover, the isolated disorder of S261–G264 within the ordered kinase domain upstream of T313 implies the existence of locally flexible regions that may vary in their sensitivity to the T313M mutation.

We identified a total of 16 post-translational modification (PTM) sites within the PINK1 sequence, including phosphorylation, ubiquitination, and acetylation sites, as presented in Supplementary Table 5.

The most prominent PTM type was phosphorylation, with ten serine and threonine residues reported as phosphorylation sites. Of these, S495 had the highest NetPhos score of 0.989, followed by S230 (0.971), S402 (0.941), and T257 (0.916). These serine and threonine residues are high-confidence phosphorylation sites, which are most likely involved in the regulation of the kinase activity of PINK1. S284 had a moderate phosphorylation score of 0.531, S261 had a score of 0.598, and S228 had a score of 0.522. T313, T313 exhibited a NetPhos score of 0.553 and has been reported as a phosphorylation-associated residue in PhosphoSitePlus. However, the present study does not experimentally evaluate the phosphorylation status of T313 or the effect of the T313M substitution on this modification. Ubiquitination sites were present at K135, K137, K164, K186, K319, and K433, whereas acetylation at K262 has already been reported. The presence of ubiquitination at K135 and K137, both of which are located within the IDR-1 boundary and the linker/transition zone, respectively, points towards the presence of a direct link between the disordered N-terminal region of PINK1.

**FIGURE 3:**
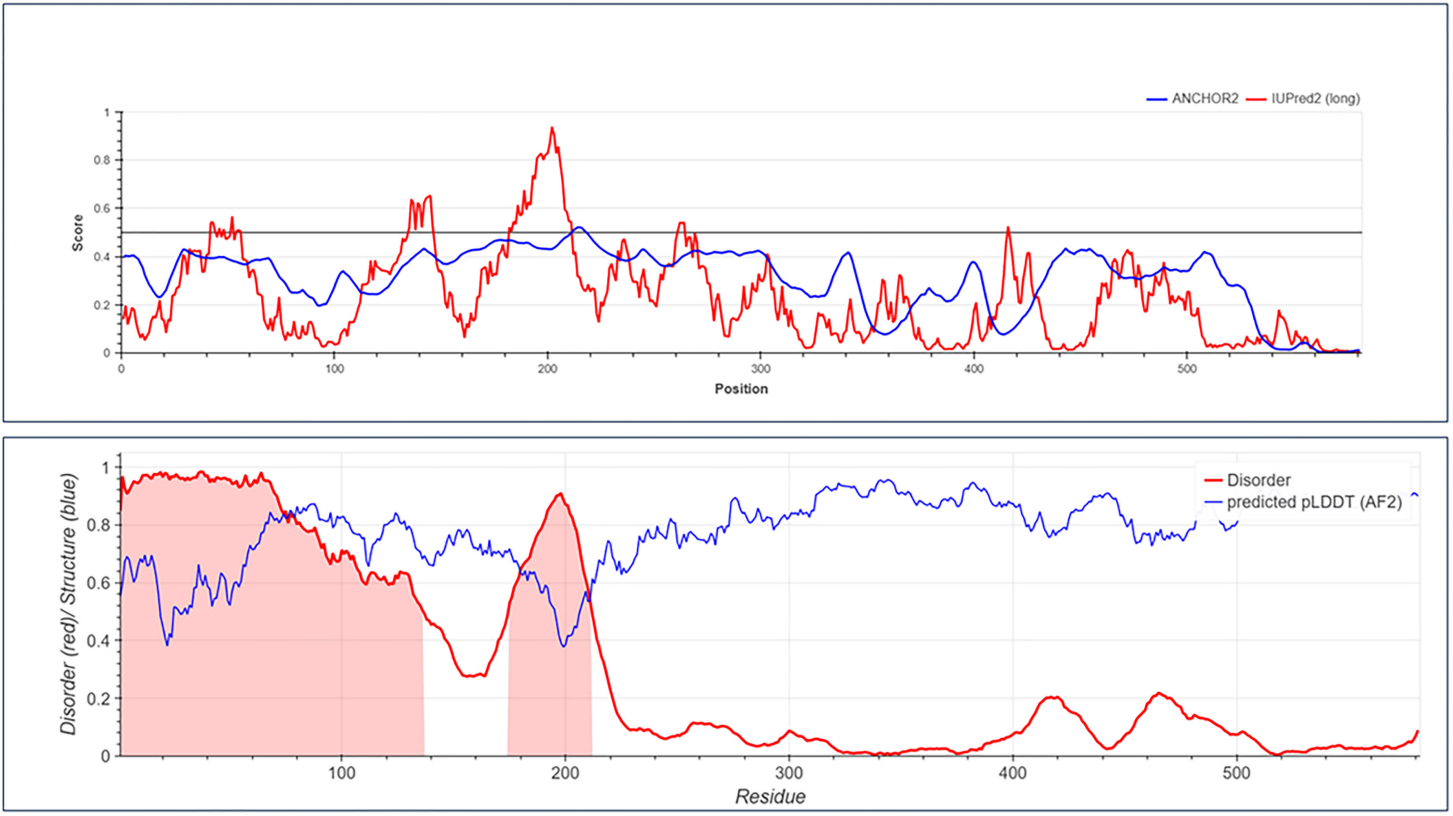
Intrinsic disorder prediction profiles of PINK1. The top panel shows the IUPred-based disorder plot, while the bottom panel represents the Metapredict output. Regions above the threshold (red) indicate predicted IDRs, whereas regions below the threshold (blue) correspond to ordered segments. Shaded regions highlight consensus disorder-prone segments across both predictors, emphasizing functionally relevant flexible regions.

**FIGURE 4:**
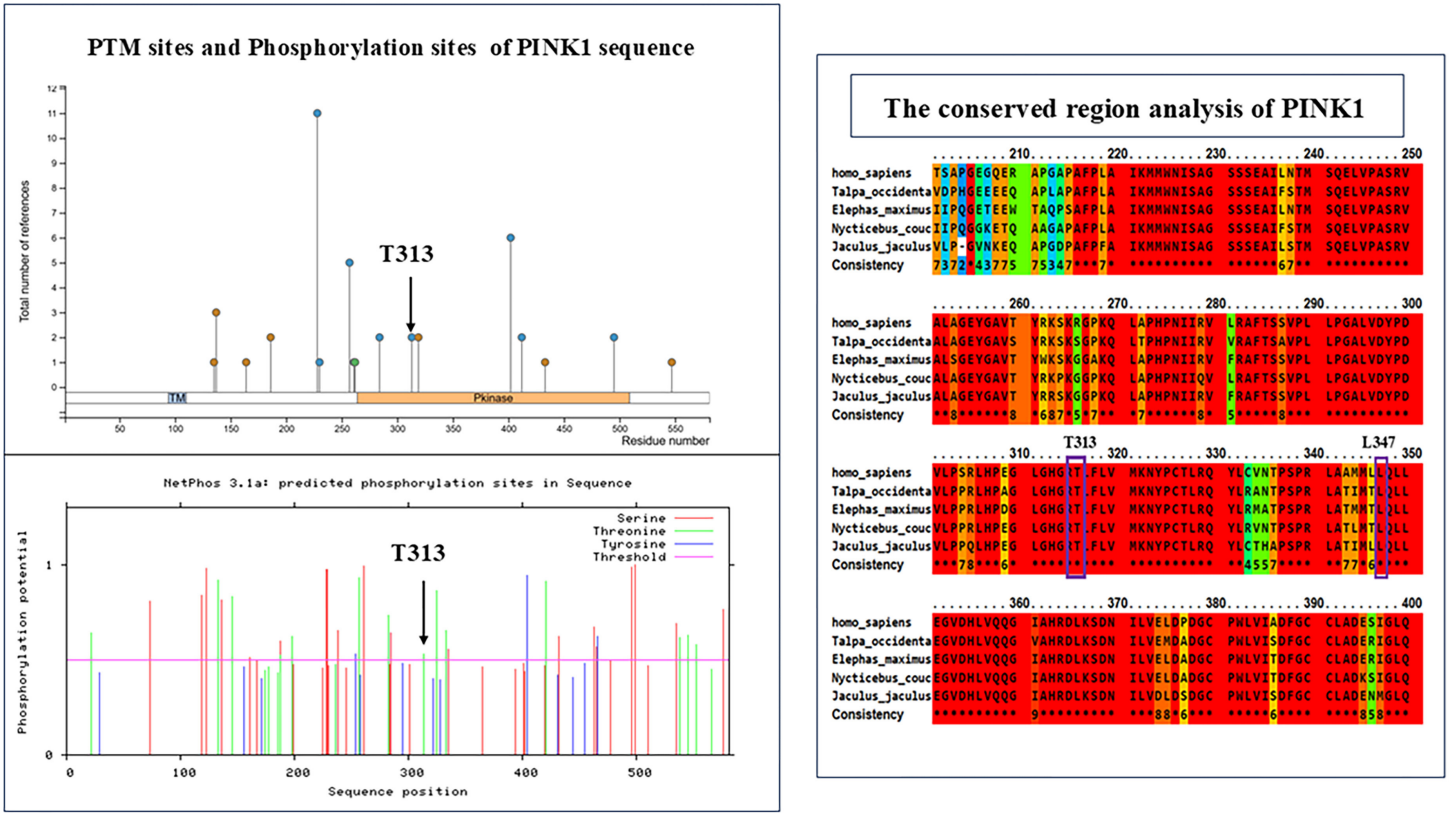
Post-translational modification and conservation analysis of PINK1. The left panel shows PTM site predictions from PhosphoSitePlus and NetPhos, highlighting key phosphorylation and ubiquitination residues across the protein. The right panel presents multiple sequence alignment (MSA) generated using PRALINE, where highly conserved residues are indicated in purple, emphasizing evolutionarily conserved regions likely critical for structural stability and functional activity.

Within this functional architecture, T313M is located near the ATP-binding region of the kinase domain and affects a residue reported to be associated with phosphorylation. Therefore, the mutation may influence local regulatory mechanisms or kinase function, although this possibility requires experimental validation. Similarly, L347P occurs within a structurally conserved region adjacent to catalytic motifs of the kinase domain. Given the known structural effects of proline substitutions, this mutation may perturb local secondary structure and catalytic-site organization; however, these effects remain computational predictions that require biochemical and structural confirmation. Taken together, the IDR and PTM analyses offer additional insight into the potential structural and functional relevance of the prioritized T313M and L347P variants identified through the SNP filtering pipeline. The findings suggest that T313M may affect a phosphorylation-associated residue located near the ATP-binding region, while L347P may influence the structural integrity of a conserved catalytic segment. Although these observations provide plausible mechanistic explanations for the potential impact of these variants, the proposed effects remain computational predictions and require validation through phosphoproteomic studies, kinase activity assays, structural investigations, and cellular functional analyses. Collectively, the results of the IDR and PTM analysis could provide a robust rationale for the pathogenicity of the T313M and L347P mutations (Figures 3, 4).

### 3.5 Characterization of evolutionary conservation and active site architecture

The conserved region analysis of PINK1 using PRALINE multiple sequence alignment from five divergent mammalian species, namely *Homo sapiens*, *Talpa occidentalis*, *Elephas maximus*, *Nycticebus coucang* and *Jaculus jaculus*, showed the evolutionary conservation of the kinase domain (Figure 4). The regions 310–330 and 341–352 showed nearly complete conservation indicated by an asterisk score in the consistency row of the output. In the 310–330 region, the invariant amino acids are T313, A321, A324, and A327. In the 341–352 region, the invariant amino acids are L343, L347, T348, and A352. In the 341–352 region, the invariant amino acids are also present. In the 310–330 region of the kinase domain of PINK1, the invariant amino acids are T313, A321, A324, and A327. In the 341–352 region specific to the kinase domain of PINK1, the invariant amino acids are L343, L347, T348, and A352

We employed Structure-based active site prediction to assess top-ranked binding pocket (Rank 1, score: 9.28, probability: 0.542, average conservation score: 1.249) that included 25 residues from three functionally unique sub-regions of the PINK1 kinase domain: the ATP-binding P-loop region (residues 162–170 and 219), substrate recognition region (residues 275, 318, 319, 321, 324, 327), and the catalytic HRD motif cluster region (residues 362, 364, 366, 367, 369, 383, 384, 386, 387, 409) with a high average conservation score of 1.249 for all residues within the identified active site. The prioritization of active residues in PINK1 for protein–protein docking was achieved through the integrated analysis of binding pocket prediction, evolutionary conservation, domain and motif annotation, PTM profiling, and SNP mutational data. The residues A321, A324, and A327 were directly chosen from the PrankWeb top-ranked pocket, where they are part of the substrate recognition sub-region, fully conserved in all five species according to the PRALINE alignment, and lie within the Ser/Thr kinase domain (316–501) in spatial proximity to the ubiquitination site K319.

The multiple sequence alignment further revealed that the prioritized missense mutations, T313M and L347P are evolutionarily well-conserved in all five closely related mammalian species. More specifically, T313 lies in the asterisked region of evolutionary conservation between positions 310–330, whereas L347 lies in the equally evolutionarily well-conserved region between positions 341–352. This further confirmed that both positions are evolutionarily well-conserved and functionally indispensable in the kinase domain of the PINK1 protein. The evolutionary conservation of these positions in combination with their spatial proximity to or overlap with the top-ranked active site pocket (score: 9.28, probability: 0.542) served to further inform the selection of the final set of PINK1 active residues to be used in the protein-protein docking. More specifically, T313, A321, A324, A327, L343, L347, T348, and A352 were prioritized as the PINK1 active residues in combination with I44, L10, K49, R72, S65, K68, and R74 as the ubiquitin active residues in accordance with their well-characterized roles as the canonical hydrophobic recognition surface, direct phosphorylation target Ser65, and flanking electrostatic interface residues that define the biologically relevant PINK1-Ubiquitin interaction surface.

### 3.6 Binding energetics of PINK1-ubiquitin complexes

**FIGURE 5:**
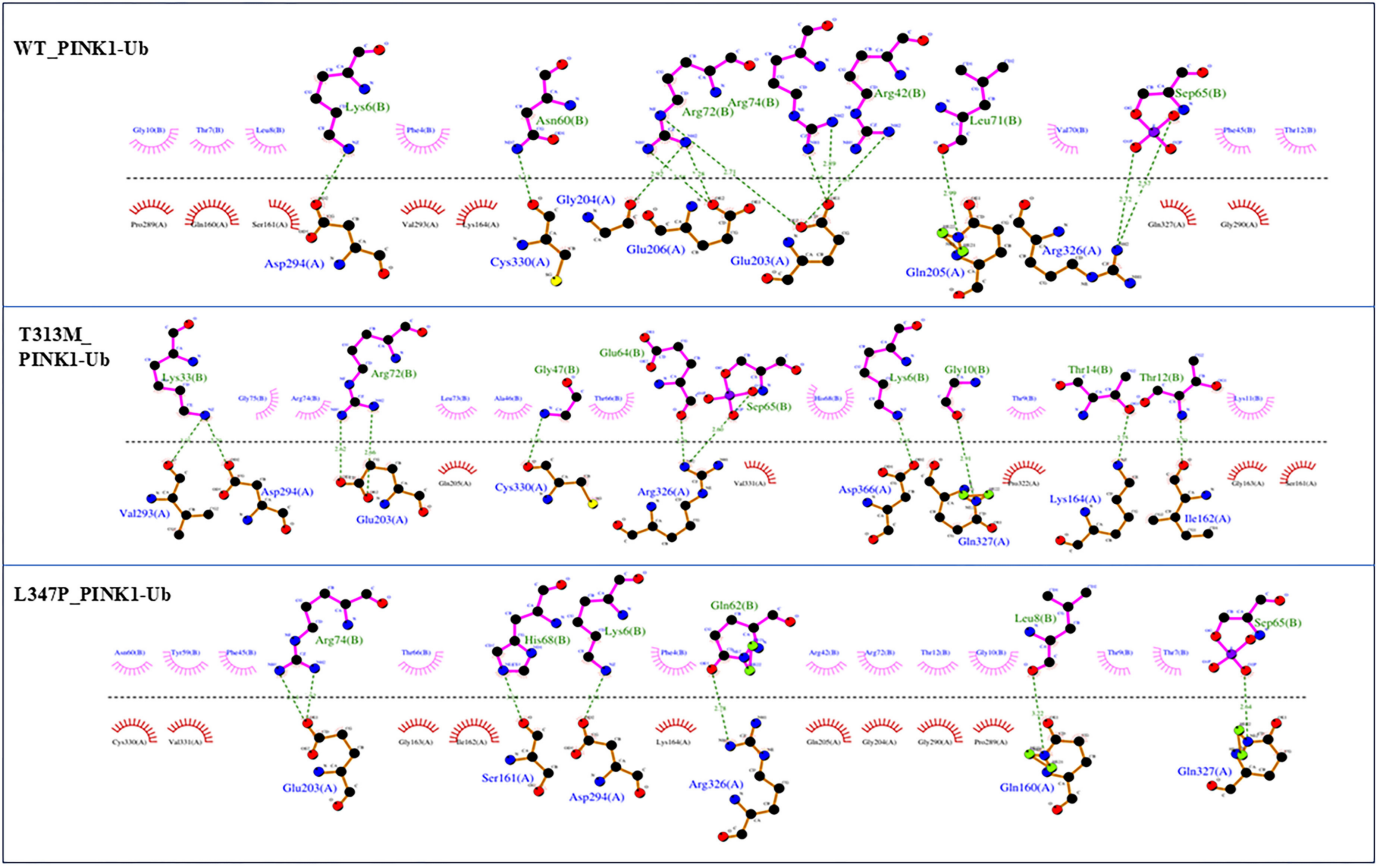
Two-dimensional interaction maps of WT and pathogenic PINK1–Ub complexes revealing conserved engagement of phosphoserine 65 (pSer65) at the binding interface.

The Parkinson’s disease-associated variants T313M and L347P were subsequently introduced into the modeled PINK1 wild-type structure using the Rotamers tool in UCSF Chimera. Side-chain conformations were selected based on favorable rotamer probabilities and minimal steric clashes. Standard protonation states corresponding to physiological pH (∼7.0) were assigned, with Asp/Glu deprotonated and Lys/Arg protonated. The resulting wild-type and mutant models were subjected to energy minimization and subsequent molecular dynamics simulations. Guided flexible protein–protein docking of wild-type PINK1 and the T313M and L347P mutant complexes with native ubiquitin was performed using the residues identified from active-site analysis to investigate the impact of the missense mutations on PINK1–Ubiquitin interactions. A total of eight active residues were defined for PINK1 (T313, A321, A324, A327, L343, L347, T348, and A352) and seven active residues for ubiquitin (I44, L8, K49, S65, K68, R72, and R74), which were used as Active Interaction Regions (AIRs) during HADDOCK docking. The docked structures were then analyzed by two-dimensional interaction analysis using LigPlot+ (refer Figure 5) to understand the hydrogen bonding and hydrophobic contacts in the interface of the docked complexes. The interaction profiles of the three complexes showed different and altered contact patterns at the interface due to the introduction of the prioritized mutations, thus providing a direct structure-based rationale for the effects of T313M and L347P on the PINK1-Ubiquitin interface. In addition, a comparative docking study demonstrated a hierarchy of binding affinity and complex stability for the post-phosphorylation state complexes. The wildtype PINK1 was identified to have the most favorable complex with Ser65-phosphorylated ubiquitin (pSer65-Ub), while the L347P mutant was next in order of preference, and finally, the T313M mutant. This order of scores for wildtype and mutants indicates a destabilization of the complex due to mutations. This is also evident from a decrease in the electrostatic interaction energy and BSA for both mutants. Among these two mutants, T313M was found to have the largest destabilization of the complex.

From a mechanistic point of view, the observed decrease in the binding affinity can be attributed mainly to a considerable loss in the electrostatic component, which is a key factor for the recognition of the phosphorylated form of the ubiquitin protein, given the negatively charged phosphate moiety at position 65 of the ubiquitin protein sequence. Although the van der Waals component is found to be mostly unchanged for all the systems under consideration, the loss in the electrostatic component and the interface for the mutant complexes point to a loss in the specific recognition of the phosphorylated form. Besides the smaller size of the complexes and the altered convergence for the mutant complexes indicate a loss in the stability and reliability of the complexes. Although a slight deviation is observed for the Z-scores for the complexes under consideration. The docking interactions and binding affinity scores have been depicted in Figure 5.

The wild-type WT_PINK1_Ub complex exhibited the best docking characteristics, with a score of −88.4 ± 8.2, signifying it as the strongest interaction among all complexes. The L347P mutant was also studied, and it was observed to have a reduced score of −81.9 ± 3.4. The worst interaction was observed for the T313M mutant, having a score of −77.4 ± 4.9. This indicates that both mutants are causing destabilization of the interaction with phosphorylated ubiquitin, but T313M is more destabilizing. Significantly, cluster analysis indicated that the wild-type complex possessed the highest level of convergence, with 123 structures falling within 10 clusters, which was the largest and most populated clustering profile, implying a stable and well-defined binding mode for the wild-type complex. In contrast, the pathogenic variants displayed a lower level of convergence, with 99 structures falling within 14 clusters for L347P, and 98 structures falling within 12 clusters for T313M, implying a higher conformational heterogeneity for these two variants, suggesting that the L347P and T313M substitutions may destabilize the native binding conformation relative to the wild-type. which may indicate a lack of conformational convergence and stability for the mutant complexes. The electrostatic interactions were the main drivers for all the complexes, which is a reflection of the presence of a negatively charged phosphate group on the ubiquitin protein at position Ser65. The electrostatic contribution was highly favorable for the WT complex, where the electrostatic energy was estimated to be −357.6 kcal/mol ± 36.3. However, this contribution was greatly reduced for L347P, where the electrostatic energy was estimated to be −260.6 kcal/mol ± 34.7. This contribution was even lower for T313M, where the electrostatic energy was estimated to be −235.5 kcal/mol ± 62.1. This Substantial decline of electrostatic stabilization in both mutants suggests a disrupted intermolecular interaction in the recognition of phosphorylated ubiquitin. Considering the pivotal importance of phospho-specific interactions in PINK1-mediated signaling, this suggests that both mutants could impair the molecular recognition necessary for the initiation of mitophagy.

Van der Waals interactions were relatively comparable for all systems, with values of −40.0 ± 7.1 kcal/mol (WT), −43.2 ± 7.6 kcal/mol (L347P), and −43.5 ± 6.0 kcal/mol (T313M). This indicates that hydrophobic packing and short-range interactions are conserved between the systems. However, a difference was observed in the desolvation energy. The WT complex has a higher positive desolvation energy, compared to the lower values for the mutants, L347P and T313M, which is a possible indicator of reduced solvent accessibility and non-optimal interface formation. The values for the mutants were 2.9 ± 2.3 kcal/mol and 3.0 ± 3.7 kcal/mol, respectively, compared to the WT value of 14.2 ± 2.5 kcal/mol. The buried surface area, which is a significant measure of interface strength, was also highest for the WT complex, at 1741.6 ± 158.8 Å^2^, followed by L347P at 1624.1 ± 234.4 Å^2^, and then T313M at 1547.4 ± 60.8 Å^2^. This reduction in buried surface area for all mutants indicates a loss of contact, which is consistent with weaker interfaces. The RMSD from the lowest-energy structure showed that, for the WT complex, there was greater conformational variation within the cluster, at 7.3 ± 0.1 Å, whereas for both mutants, their RMSD was ∼3.8 Å. Although this could imply structural similarity within the cluster, it does not necessarily imply better binding but may imply that both mutants are adopting a less optimal but more constrained mode of binding. Restraints violation energy was also higher in the L347P mutant (105.7 ± 20.2 kcal/mol) and T313M mutant (101.3 ± 38.6 kcal/mol) compared to WT (89.1 ± 34.4 kcal/mol), suggesting that the mutant systems deviated more from ideal docking restraints. The Z-scores for the top clusters were −1.6 for WT, −1.8 for L347P, and −1.4 for T313M. Though the Z-score for the top cluster in the L347P mutant was slightly lower than that for WT, it should be noted that the Z-score is a measure that depends on the distribution of the clusters for a particular docking simulation. Therefore, it is not possible to compare the Z-scores for the different systems. Instead, it suggests that the top cluster for each system is one of the most reliable among all the clusters for a particular docking simulation.

Altogether, the docking outcomes suggest that the wild-type PINK1 forms the most stable and favorable complex with the phosphorylated form of ubiquitin. Moreover, the L347P and T313M mutations both weaken the interaction between PINK1 and the phosphorylated form of ubiquitin; however, the T313M mutation appears to weaken the interaction more significantly than the L347P mutation. This suggests a more significant impact on the recognition of the phosphorylated form of ubiquitin. This interaction profile is a supportive computational finding that these mutations in the PINK1 can influence the interaction between PINK1 and the phosphorylated form of ubiquitin. The interaction scores and energies have been depicted in Table 2.

**TABLE 2:** Protein–protein docking analysis of wild-type and mutant PINK1–ubiquitin complexes.

Complexes	HADDOCK score	Cluster size	RMSD (Å)	Van der waals energy (kcal/mol)	Electrostatic energy (kcal/mol)	Desolvation energy (kcal/mol)	Buried surface area (Å^2^)	Z-score
WT_PINK1_Ub	−88.4 ± 8.2	34	7.3 ± 0.1	−40 ± 7.1	−357.6 ± 36.3	14.2 ± 1.25	1741.6 ± 158.8	−1.6
L347P_PINK1_Ub	−81.9 ± 3.4	16	3.8 ± 0.2	−43.2 ± 7.6	260.6 ± 34.7	2.9 ± 2.3	1624.1 ± 234.4	−1.8
T313M _PINK1_Ub	−77.4 ± 4.9	15	3.8 ± 0.1	−43.5 ± 6.0	−235.5 ± 62.1	3.0 ± 1.37	1547.4 ± 60.8	−1.4

Docking was performed using HADDOCK, and the resulting complexes were evaluated based on HADDOCK score, cluster size, RMSD, van der Waals and electrostatic energies, desolvation energy, buried surface area, and Z-score. Comparative analysis highlights the impact of T313M and L347P mutations on the binding affinity and structural stability of the PINK1–ubiquitin interaction.

### 3.7 Mutation-induced structural reorganization through flexibility, compactness and solvent exposure

**FIGURE 6:**
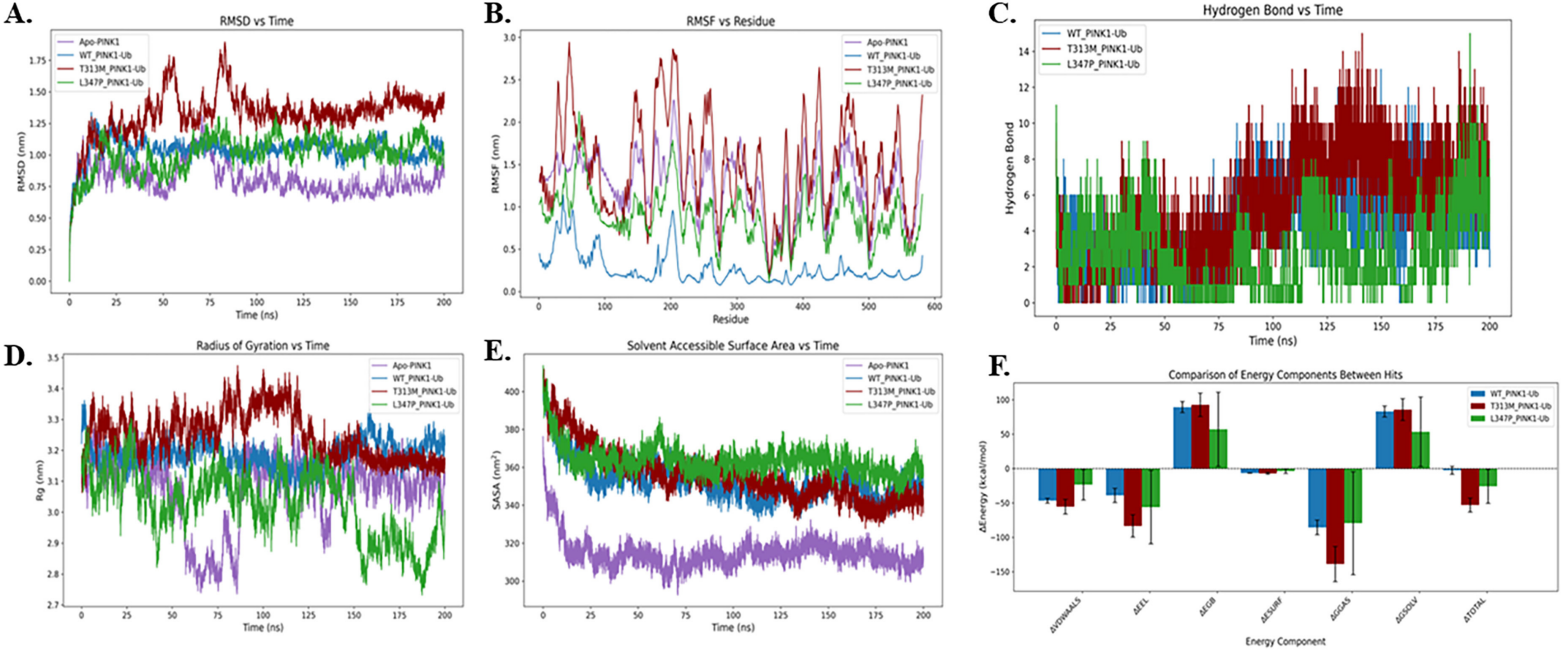
Comparative molecular dynamics analysis of WT_PINK1–Ub, T313M_PINK1–Ub, and L347P_PINK1–Ub systems (Replicate 1). **(A)** Root mean square deviation (RMSD), **(B)** root mean square fluctuation (RMSF), **(C)** intermolecular hydrogen bond profile, **(D)** radius of gyration (Rg), **(E)** solvent-accessible surface area (SASA), and **(F)** MM/PBSA energy decomposition analyses for WT_PINK1–Ub, T313M_PINK1–Ub, and L347P_PINK1–Ub complexes during the 200 ns molecular dynamics simulation. RMSD, RMSF, Rg, and SASA analyses were used to evaluate structural stability, flexibility, compactness, and solvent exposure, respectively. MM/PBSA energy decomposition was performed to assess the contribution of individual energetic terms to complex stability.

**FIGURE 7:**
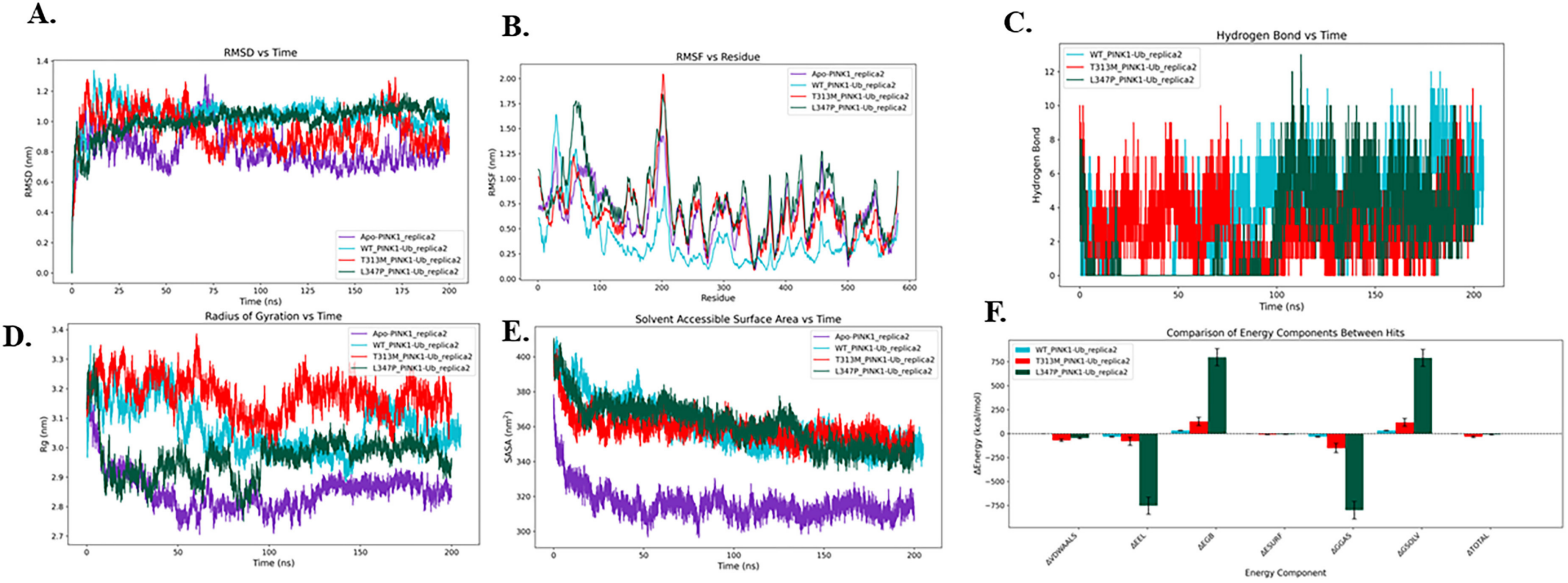
Comparative molecular dynamics analysis of WT_PINK1–Ub, T313M_PINK1–Ub, and L347P_PINK1–Ub systems (Replicate 2). **(A)** RMSD, **(B)** RMSF, **(C)** intermolecular hydrogen bonds, **(D)** radius of gyration, **(E)** SASA, and **(F)** MM/PBSA energy decomposition profiles obtained from the second independent molecular dynamics simulation replicate. The analyses were performed to evaluate the reproducibility of mutation-induced structural and energetic changes observed across replicate simulations.

**FIGURE 8:**
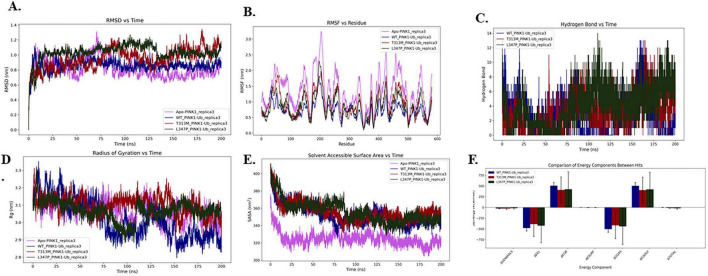
Comparative molecular dynamics analysis of WT_PINK1–Ub, T313M_PINK1–Ub, and L347P_PINK1–Ub systems (Replicate 3). **(A)** RMSD, **(B)** RMSF, **(C)** intermolecular hydrogen bond profile, **(D)** radius of gyration, **(E)** SASA, and **(F)** MM/PBSA energy decomposition analyses obtained from the third independent simulation replicate. Consistency across replicates was assessed to improve confidence in the observed mutation-associated dynamic behavior.

To improve the statistical robustness of the molecular dynamics (MD) analyses, three independent 200-ns simulations were performed for the WT PINK1–ubiquitin complex and the T313M and L347P mutant systems. Average values and standard deviations were calculated for RMSD, RMSF, radius of gyration (Rg), solvent-accessible surface area (SASA), and intermolecular hydrogen-bond interactions. The replicate plots have been presented in Figures 6–8, respectively.

Analysis of structural deviations revealed that the WT complex maintained a relatively stable conformational profile throughout the simulations, whereas both mutant systems exhibited increased structural fluctuations. The T313M variant displayed the highest average RMSD (1.0700 ± 0.2185 nm), followed by L347P (1.0177 ± 0.0140 nm) and WT (0.9785 ± 0.1050 nm), indicating greater deviation from the initial structure and suggesting mutation-induced destabilization of the complex. Consistent with this observation, residue-level flexibility analysis demonstrated markedly elevated RMSF values for T313M (1.0189 ± 0.4278 nm) and L347P (0.8416 ± 0.0593 nm) relative to WT (0.4521 ± 0.2223 nm), indicating increased local mobility in the mutant systems. Residue-wise RMSF mapping provided further mechanistic insight into mutation-induced conformational changes. The WT complex exhibited relatively uniform fluctuations across the protein sequence, with only minor flexible segments. In contrast, the T313M mutant displayed multiple highly dynamic regions spanning residues 39–62, 142–157, 174–213, 250–263, 420–428, and 464–480. The most pronounced fluctuations were observed around residues 201–209, where RMSF values exceeded 2.4 nm. The L347P mutant also exhibited enhanced flexibility but predominantly within a more localized region encompassing residues 193–207, with peak RMSF values approaching 1.9 nm. These findings indicate that T313M induces widespread conformational perturbations, whereas L347P primarily causes localized structural rearrangements. The radius of gyration analysis further supported these observations. T313M exhibited the highest Rg value (3.1760 ± 0.0733 nm), indicating reduced structural compactness compared with WT (3.0923 ± 0.0843 nm). In contrast, L347P displayed an Rg value (3.0199 ± 0.0528 nm) comparable to WT, suggesting that its destabilizing effects are less pronounced at the level of global protein compactness. Together, the RMSD, RMSF, and Rg analyses consistently identify T313M as the variant associated with the greatest dynamic instability. SASA analysis demonstrated increased solvent exposure in both mutant complexes relative to WT. Interestingly, L347P exhibited the highest average SASA value (360.2930 ± 3.7158 nm^2^), followed by T313M (356.9244 ± 1.6072 nm^2^) and WT (355.3442 ± 4.5139 nm^2^). This observation suggests that although T313M displays the strongest dynamic destabilization, L347P promotes greater solvent accessibility of the protein surface. Therefore, the two mutations may affect the PINK1–ubiquitin complex through distinct structural mechanisms. Intermolecular hydrogen-bond analysis revealed differences in interface dynamics among the systems. T313M maintained a slightly higher average number of hydrogen bonds (3.7720 ± 1.4532) than WT (3.4606 ± 0.7426), whereas L347P exhibited fewer hydrogen bonds (3.1206 ± 0.8755). However, the larger fluctuations observed in the mutant systems indicate increased dynamic rearrangement of interfacial contacts. Thus, hydrogen-bond counts alone do not fully capture interface stability and should be interpreted together with RMSD, RMSF, and conformational analyses. The clustered docking solutions for all three PINK1–ubiquitin complexes using Ambiguous Interaction Restraints (AIRs) defined for the predicted PINK1 interface residues 313, 321, 324, 327, 340, 347, 348, and 352. For the wild-type PINK1–ubiquitin complex, 109 water-refined models (54% of the generated models) were grouped into 15 clusters, with Cluster 12 identified as the top-ranked cluster. The T313M mutant yielded 101 clustered models (50%) distributed across 13 clusters, with Cluster 8 selected as the highest-ranked solution. The L347P mutant produced 91 clustered models (45%) organized into 11 clusters, with Cluster 2 representing the top-ranked cluster. For all systems, the highest-ranked cluster was selected based on the most favorable HADDOCK score and Z-score for subsequent interface characterization and molecular dynamics simulations. Trajectory inspection demonstrated stabilization of all systems following the initial equilibration phase, supporting convergence of the simulations. Nevertheless, both mutant systems sampled broader conformational spaces than WT, consistent with their elevated RMSD and RMSF values. Collectively, the replicate-supported analyses indicate that the WT PINK1–ubiquitin complex maintains the most stable interaction interface, whereas T313M induces the greatest conformational perturbation and dynamic instability. L347P exhibits a milder destabilizing effect but is associated with increased solvent exposure, suggesting a distinct mechanism of structural alteration.

Although replicate simulations substantially improved the robustness of the molecular dynamics analyses, several limitations should be acknowledged. The simulations remain constrained by the accessible timescale and the assumptions inherent to the employed force field and solvent model. While convergence was observed for the analyzed trajectories, longer simulations could reveal additional conformational states not sampled within the current simulation window. Furthermore, different structural descriptors highlighted distinct aspects of mutation-induced perturbation. Specifically, T313M consistently exhibited the greatest dynamic instability based on RMSD, RMSF, and Rg analyses, whereas L347P displayed the highest solvent-accessible surface area. Therefore, the mutations likely alter PINK1–ubiquitin interactions through different structural mechanisms rather than a single uniform destabilization pathway warranting experimental validation and extended simulations would be valuable for further confirming these observations. The average of the replicates have been depicted in Supplementary Table 6 and the intermolecular interactions have been plotted in Supplementary Figure 5.

#### 3.7.1 MM/PBSA binding free energy decomposition and energetic interpretation

MM/PBSA calculations were performed to estimate the binding energetics of the WT_PINK1–Ub, T313M_PINK1–Ub, and L347P_PINK1–Ub complexes. The calculated mean binding free energies were −1551.84 ± 559.87 kcal/mol for WT, −1547.44 ± 556.01 kcal/mol for T313M, and −1546.54 ± 558.16 kcal/mol for L347P. All systems exhibited negative binding free energy values, indicating thermodynamically favorable complex formation. Although minor differences in mean binding free energy were observed among the systems, these differences were small relative to the associated standard deviations. Consequently, the MM/PBSA results do not provide sufficient evidence to support substantial mutation-induced changes in binding affinity or direct impairment of PINK1–ubiquitin recognition. Instead, the comparable binding free energies suggest that the overall thermodynamic favourability of complex formation is largely preserved in both mutant systems. Therefore, the functional consequences of T313M and L347P are more likely to arise from mutation-associated alterations in structural stability, conformational flexibility, and dynamic behavior observed during molecular dynamics simulations rather than from major changes in binding energetics. The MM/PBSA results should thus be interpreted as supporting broadly similar binding affinities across the three systems while highlighting the importance of dynamic and structural perturbations in understanding the potential effects of these variants. The MM/PBSA plots for replicates have been depicted in Figures 6–8. The mean and standard deviation values have been represented in Supplementary Table 6, respectively.

### 3.8 Conformational landscape analysis: PCA- derived free energy landscape analysis and dynamic cross-correlation

To further investigate the impact of the T313M and L347P mutations on the dynamic behavior of the PINK1–ubiquitin complex, principal component analysis (PCA) and dynamic cross-correlation matrix (DCCM) analyses were performed. PCA revealed that the WT complex occupied a relatively compact conformational space characterized by a dominant low-energy basin, indicating stable structural dynamics throughout the simulation. In contrast, both mutant systems explored broader conformational landscapes, reflecting increased flexibility and conformational heterogeneity. Among the variants, T313M exhibited the widest distribution across the principal components and sampled multiple metastable states, suggesting substantial disruption of the native conformational equilibrium. The L347P mutant also displayed an expanded conformational space compared with WT but remained less dispersed than T313M.

**FIGURE 9:**
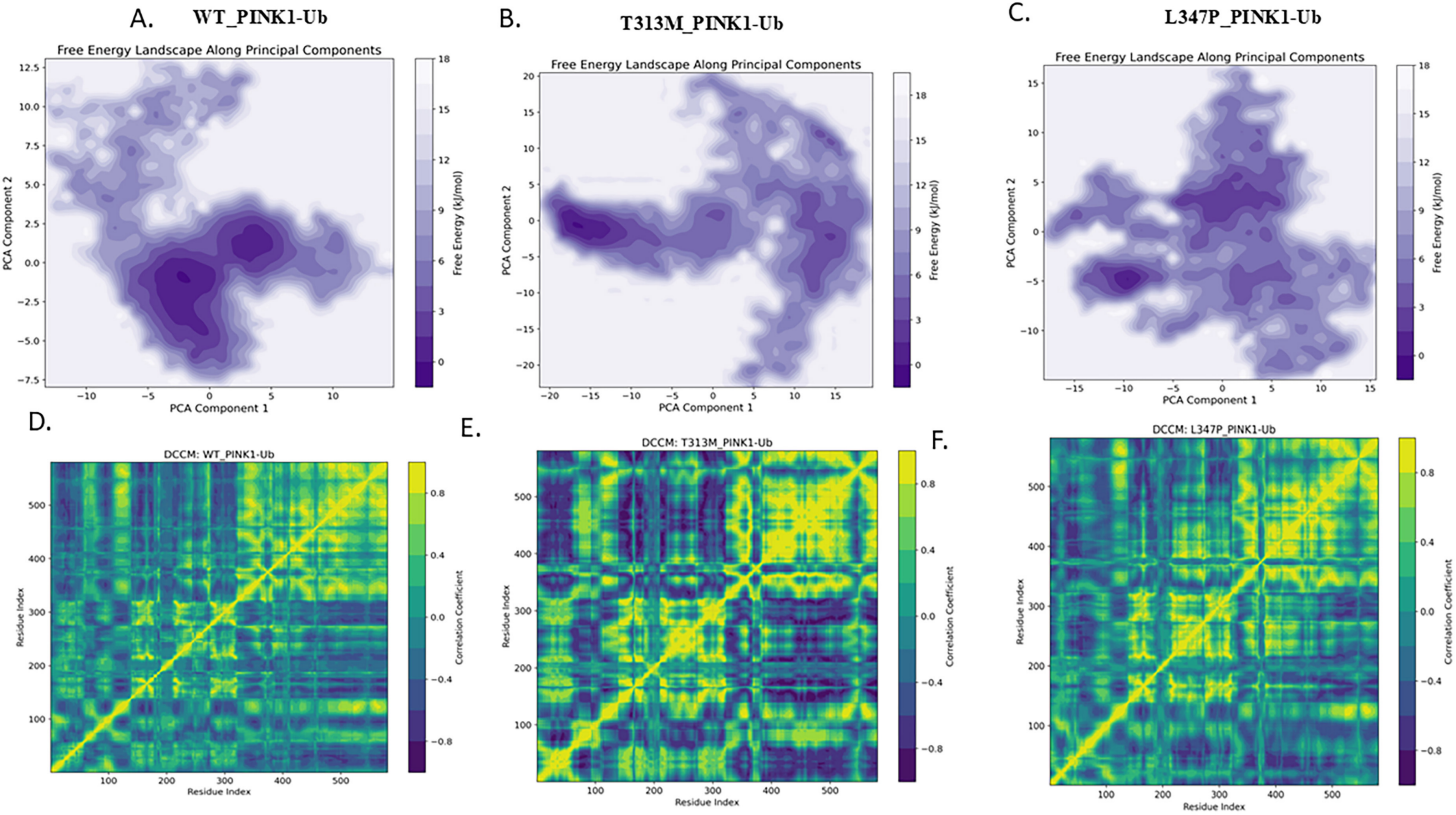
Free energy landscape (FEL) and dynamic cross-correlation matrix (DCCM) analyses of WT and pathogenic PINK1–Ub complexes. **(A–C)** Free Energy Landscapes along principal components (PC1 vs. PC2) **(A)** WT_PINK1_Ub displays a single broad, deep energy minimum, indicating a well-defined and stable conformational ensemble. **(B)** T313M_PINK1_Ub shows a widely dispersed, shallow energy landscape with multiple minima, reflecting extensive conformational heterogeneity and reduced stability. **(C)** L347P_PINK1_Ub exhibits a fragmented landscape with several discrete low-energy basins, suggesting increased conformational sampling and partial destabilization relative to WT. **(D–F)** Dynamic Cross-Correlation Matrices (DCCM). **(D)** WT_PINK1_Ub shows predominantly correlated (positive, yellow) motions along structured regions with limited anti-correlated movements, consistent with coordinated domain dynamics. **(E)** T313M_PINK1_Ub displays a marked increase in both correlated and anti-correlated (negative, purple) residue motions across large regions, indicative of mutation-induced allosteric perturbations and loss of coordinated movement. **(F)** L347P_PINK1_Ub shows an intermediate pattern with expanded anti-correlated regions compared to WT, suggesting that the L347P substitution disrupts inter-domain communication. Together, FEL and DCCM analyses reveal that both pathogenic mutations destabilize the conformational and dynamic organization of the PINK1–Ub complex.

Consistent with the PCA findings, DCCM analysis demonstrated that the WT complex maintained a balanced network of correlated and anti-correlated residue motions. The DCCM correlation coefficient ranges from −1 to +1, where +1 represents fully correlated motions, −1 represents fully anti-correlated motions, and 0 indicates uncorrelated movements between residue pairs. The WT system displayed well-defined positive and negative correlation regions, indicative of coordinated intramolecular communication and stable ubiquitin-associated dynamics. In contrast, both mutations altered these dynamic communication networks. The T313M variant showed the most pronounced redistribution of correlated motions, accompanied by disruption of long-range residue coupling patterns observed in the WT system. Similarly, L347P modified residue-residue communication networks, although the extent of perturbation was comparatively moderate. The altered correlation patterns suggest that the mutations impair the coordinated motions required for maintaining native structural and functional interactions within the PINK1–ubiquitin complex. The PCA contours and the DCCM plots have been presented in Figure 9 and the other two replicates have been depicted in Supplementary Figures 3, 4. While the mean of MM/PBSA replicates are reported in Supplementary Table 7, respectively.

Taken together, the PCA and DCCM analyses demonstrate that the pathogenic mutations induce both conformational and communication-level perturbations. The broader conformational sampling observed in PCA corresponds with the disruption of residue correlation networks detected by DCCM, indicating that mutation-induced structural flexibility is accompanied by altered dynamic coupling between distant protein regions. Among the studied variants, T313M consistently exhibited the greatest deviation from WT behavior, suggesting a stronger destabilizing effect on the dynamic architecture of the PINK1–ubiquitin complex. These findings are in agreement with RMSD, RMSF, and radius of gyration analyses, collectively supporting the conclusion that T313M causes the most substantial dynamic perturbation, whereas L347P produces a moderate but measurable disruption of protein stability and communication. Dynamic cross-correlation matrix (DCCM) plots showing correlated and anti-correlated residue motions in WT, T313M, and L347P PINK1–ubiquitin complexes. Correlation coefficients range from −1 (strong anti-correlated motion; blue) to +1 (strong correlated motion; red), with values near 0 indicating weak or absent correlation.

## 4 Conclusion

In this study, we employed an integrative computational framework to assess the potential structural and functional ramifications of human PINK1 mutations in the ubiquitin binding interface. Structural assessment confirmed the modeled PINK1’s acceptable stereochemical characteristics, laying a groundwork for ensuing further computational analyses. Consistent with this, the conserved catalytic architecture and structurally coherent ATP–Mg^2 +^ binding environment offer a structurally informed context for examining the potential effects of missense mutations within the PINK1 kinase domain and ubiquitin-binding interface. An extensive multi-step SNP ranking protocol invariably identified T313M and L347P as the most damaging variants, followed by sequence conservation, structural interment, and predicted thermodynamic instability. Scrutiny of intrinsically disordered regions and prospective post-translational modifications revealed that, notwithstanding their placement within the structured kinase domain, the mutations’ adjacency to dynamic regulatory domains could undermine conformational pliability, phosphorylation likelihood, and interaction kinetics, thus extending their disruptive effects beyond localized structural perturbations. Phylogenetic conservation profiling and active-site prediction established that residues T313 and L347 occupy evolutionarily preserved functional territories and lie in spatial proximity to the ubiquitin-binding interface. Subsequently, these mutations exemplify discrete mechanistic archetypes: L347P occupies a profoundly conserved kinase domain expanse that intersects the inferred active site, portending overt compromise of structural coherence and catalytic residue alignment; Comparatively, T313M corresponds to a forecasted phosphorylation locus proximate to regulatory elements, intimating that its principal repercussions derive from attenuated post-translational modification. Previous studies have suggested that pathogenic mutations occurring at phosphorylation sites can disrupt PTM-dependent protein–protein interactions, particularly within intrinsically disordered regions, thereby altering signaling mechanisms (Rrustemi et al., 2024).

This mechanistic dichotomy between L347P and T313M is further substantiated by the overarching structural architecture. Although both mutations reside within the structured kinase domain, their juxtaposition to intrinsically disordered regions particularly the highly binding-prone IDR-2 implies interconnections between localized disruptions and distal conformational dynamics. Accordingly, L347P is positioned within the kinase domain and is predicted to influence the ubiquitin-binding interface due to its proximity to conserved structural elements, whereas T313M may exert more indirect effects by perturbing regulatory features, including phosphorylation-associated interactions and allosteric communication with adjacent flexible regions. Notably, both variants have been reported in association with Parkinson’s disease. The T313M mutation, located within the PINK1 kinase domain, segregates with early-onset Parkinson’s disease in a recessive manner, indicating a loss-of-function effect. Given the central role of the kinase domain in ubiquitin phosphorylation, this mutation is likely to impair PINK1-mediated activation of the ubiquitin–Parkin pathway (Chishti et al., 2006; Doostzadeh et al., 2007; Luo et al., 2014; Moriwaki et al., 2008) and disruption of PINK1 function has been shown to impair mitophagy and mitochondrial homeostasis, highlighting the importance of PINK1 integrity in maintaining cellular quality control mechanisms (Beilina et al., 2005; Hu et al., 2025).

Molecular docking simulations corroborate this paradigm, disclosing attenuated interaction stability across both mutants most acutely in T313M and vitiated electrostatic contributions vital for phosphorylated ubiquitin discernment. Protein–protein docking specifically demonstrated that the wild-type complex manifests optimal binding to phosphorylated ubiquitin, while the mutants, especially T313M, evince diminished affinity, enfeebled electrostatic engagements, and contracted buried surface areas. As electrostatic forces are indispensable for apprehending the anionic phosphate moiety at Ser65, their abatement furnishes a mechanistic underpinning for deficient phospho-specific recognition. Molecular dynamics simulations reinforce these structural inferences, unmasking compromised stability and aberrant dynamic profiles in mutant complexes vis-à-vis the wild-type. Across an array of trajectory-derived metrics encompassing RMSD, RMSF, radius of gyration, and SASA. The mutants displayed amplified structural deviations, eroded compactness, and modified solvent exposure. These perturbations may conjointly attest that T313M and L347P destabilize the key- intrinsic rigidity of the PINK1–Ub complex. Hydrogen bonding scrutiny and contact durability assessments further indicate that mutants may not perpetuate enduring intermolecular liaisons across the simulation duration. Whereas the wild-type sustains coherent interaction matrices, the mutants proffer episodic and tenuous associations, consonant with eroded interfacial robustness. Principal Component Analysis unveils divergences in the conformational terrains traversed by wild-type and mutant ensembles. The wild-type complex populates a circumscribed, thermodynamically privileged basin, signifying a stable, converged structural ensemble. By contrast, T313M and L347P mutants span broader, more diffuse realms in principal component coordinates, indicating increased conformational heterogeneity.

The corresponding free energy landscapes further demonstrate that mutant systems populate multiple shallow minima rather than a single dominant basin which is consistent with reduced interface stability, increased conformational heterogeneity, and altered dynamic behavior rather than a substantial reduction in binding affinity. Notably, T313M exhibits a greater degree of dispersion compared to L347P, aligning with its more pronounced destabilizing effect observed in docking and energetic analyses. Binding free energy calculations using the MM/PBSA approach indicate broadly comparable binding free energies across the wild-type and mutant complexes. Although minor differences in the energetic components were observed, these were small relative to the associated variability and do not support substantial mutation-induced changes in overall binding affinity. Instead, the molecular dynamics analyses suggest that the functional consequences of the mutations arise primarily from altered interface stability, conformational flexibility, and dynamic behavior. Given that ATP–Mg^2 +^-dependent kinase activity is expected to rely on precise electrostatic environments, the observed electrostatic differences may contribute to altered functional interactions; however, these observations are computational predictions that require experimental validation.

Dynamic cross-correlation matrix (DCCM) analysis provides additional insight into how these mutations influence intramolecular communication networks. The wild-type complex exhibits coordinated correlated motions between the kinase domain and the ubiquitin-binding interface, reflecting an efficient transmission of dynamic signals necessary for substrate recognition and catalysis. In contrast, both mutants show a reduction in these correlated motions, accompanied by an increase in anti-correlated regions. This disruption of coordinated dynamics suggests that the mutations impair long-range communication pathways within the protein. For T313M, this effect may be linked to its proximity to flexible and regulatory regions, including IDR-2, supporting a potential allosteric mechanism. For L347P, the disruption appears more structurally localized but still propagates to affect global dynamic behavior. Within the context of ATP–Mg^2 +^-dependent kinase stabilization, these perturbations may compromise the precise structural and electrostatic environment required for efficient catalysis and substrate recognition.

Collectively, these findings converge to indicate that mutations such as T313M (Al-Mubarak et al., 2015) and L347P induce subtle yet functionally significant perturbations in structural stability, The PTM analysis suggests that Thr313 may represent a functionally important phosphorylation-associated residue in PINK1. Previous experimental studies have shown that phosphorylation at Thr313 contributes to PINK1 kinase activity, and that the T313M substitution can impair phosphorylation-dependent regulation. Similarly, L347P is predicted to perturb the structural integrity of the kinase domain (Guo et al., 2017; Luo et al., 2014). Furthermore, although the identified IDRs are spatially distinct from the kinase-domain variants, they may participate in PTM-mediated regulatory mechanisms; however, their functional relationship with T313M and L347P remains to be established, further offering hypothesis-generating insights to a deeper understanding of the molecular determinants underlying mitochondrial dysfunction in early-onset Parkinson’s disease (Iakoucheva et al., 2004; Valente et al., 2004). Clinical diagnosis of PD remains primarily grounded in a detailed medical history and neurological examination, with emphasis on hallmark motor features such as bradykinesia, resting tremor, rigidity, and postural instability. While this clinical framework is essential, it often reflects a stage at which substantial neuronal loss has already occurred, thereby limiting insight into the early molecular events driving disease onset. In this regard, there is a compelling need to incorporate early genetic screening for ARPD associated genes, particularly PINK1, whose functional role in mitophagy is central to mitochondrial quality control. Mutations in PINK1 can impair the initiation and regulation of mitophagic pathways well before overt clinical manifestations emerge. Consequently, identifying such variants at or near disease onset rather than after symptom progression offers a more meaningful opportunity to delineate how mitophagy dysfunction contributes to PD pathogenesis. Prioritizing early-stage genetic screening, especially in individuals with early-onset presentations in their bloodline, may therefore enhance mechanistic stratification of the disease by linking clinical phenotypes with underlying mitochondrial and mitophagic defects. Our study’s approach does not imply reversal of established neurodegeneration, but rather underscores the importance of capturing the disease at a stage where its molecular determinants, including mitophagy impairment, can be more accurately understood and contextualized. The present study is based entirely on computational analyses, including pathogenicity prediction, structural modeling, molecular docking, molecular dynamics simulations, and binding free-energy calculations. Consequently, no experimental validation, such as biochemical, cellular, or animal-based functional assays, was performed to confirm the predicted effects of the investigated variants. In addition, no clinical or patient-derived datasets were used to validate the proposed associations between the identified mutations and disease phenotypes. Therefore, the structural and functional consequences of T313M and L347P described herein should be regarded as computationally derived hypotheses that require further experimental and clinical investigation.

## Data Availability

The original contributions presented in the study are included in the article/Supplementary material, further inquiries can be directed to the corresponding author.

## Footnotes

1
https://alphafold.ebi.ac.uk/entry/AF-Q9BXM7-F1

2
https://spdbv.unil.ch/

3
https://www.rcsb.org/structure/1UBQ

4
https://www.uniprot.org/uniprotkb/Q9BXM7/entry

5
https://www.uniprot.org/uniprotkb/Q99MQ3/entry

6
https://www.uniprot.org/uniprotkb/D6WMX4/entry#sequences

7
https://www.genome.jp/tools-bin/clustalw

8
https://sift.bii.a-star.edu.sg/

9
http://genetics.bwh.harvard.edu/pph2/

10
https://www.ensembl.org/vep

11
https://www.ncbi.nlm.nih.gov/clinvar

12
https://snps-and-go.biocomp.unibo.it/snps-and-go/

13
https://evemodel.org/

14
https://missense3d.bc.ic.ac.uk/

15
https://cupsat.brenda-enzymes.org/

## References

[B1] Adzhubei I. Jordan D. Sunyaev S. (2013). Predicting functional effect of human missense mutations using PolyPhen-2. *Curr. Protoc. Hum. Genet.* 76:7.20.1–7.20.41. 10.1002/0471142905.hg0720s76 23315928 PMC4480630

[B2] Al-Mubarak B. Bohlega S. Alkhairallah T. Magrashi A. AlTurki M. Khalil D. et al. (2015). Parkinson’s disease in Saudi patients: A genetic study. *PLoS One* 10:e0135950. 10.1371/journal.pone.0135950 26274610 PMC4537238

[B3] Bayne A. Trempe J. (2019). Mechanisms of PINK1, ubiquitin and Parkin interactions in mitochondrial quality control and beyond. *Cell. Mol. Life Sci.* 76 4589–4611. 10.1007/s00018-019-03203-4 31254044 PMC11105328

[B4] Beilina A. Van Der Brug M. Ahmad R. Kesavapany S. Miller D. Petsko G. et al. (2005). Mutations in PTEN-induced putative kinase 1 associated with recessive parkinsonism have differential effects on protein stability. *Proc. Natl. Acad. Sci. U S A.* 102 5703–5708. 10.1073/pnas.0500617102 15824318 PMC556294

[B5] Blom N. Gammeltoft S. Brunak S. (1999). Sequence and structure-based prediction of eukaryotic protein phosphorylation sites. *J. Mol. Biol.* 294 1351–1362. 10.1006/jmbi.1999.3310 10600390

[B6] Bugnon M. Goullieux M. Röhrig U. Perez M. Daina A. Michielin O. et al. (2023). SwissParam 2023: A modern web-based tool for efficient small molecule parametrization. *J. Chem. Inf. Model.* 63 6469–6475. 10.1021/acs.jcim.3c01053 37853543 PMC10649791

[B7] Capriotti E. Calabrese R. Fariselli P. Martelli P. Altman R. Casadio R. W. S. - (2013). SNPs&GO: A web server for predicting the deleterious effect of human protein variants using functional annotation. *BMC Genomics* 14:S6. 10.1186/1471-2164-14-S3-S6 23819482 PMC3665478

[B8] Chamraj S. A. (2023). Rare and novel pathogenic variant of the PINK1 gene associated with early-onset of Parkinson’s disease - A case study. *Med. Case Rep. J.* 6 1–4. 10.31531/2581-5563.1000129

[B9] Chishti M. Bohlega S. Ahmed M. Loualich A. Carroll P. Sato C. et al. (2006). T313M PINK1 mutation in an extended highly consanguineous Saudi family with early-onset Parkinson disease. *Arch. Neurol.* 63 1483–1485. 10.1001/archneur.63.10.1483 17030667

[B10] Cogan G. Lesage S. Brice A. (2025). The genetics of autosomal recessive early-onset Parkinson’s disease. *Curr. Opin. Neurobiol.* 95:103141. 10.1016/j.conb.2025.103141 41253044

[B11] Doostzadeh J. Tetrud J. Allen-Auerbach M. Langston J. Schüle B. (2007). Novel features in a patient homozygous for the L347P mutation in the PINK1 gene. *Parkinsonism Relat. Disord.* 13 359–361. 10.1016/j.parkreldis.2006.08.009 17055324

[B12] Frazer J. Notin P. Dias M. Gomez A. Min J. Brock K. et al. (2021). Disease variant prediction with deep generative models of evolutionary data. *Nature* 599 91–95. 10.1038/s41586-021-04043-8 34707284

[B13] Gan Z. Callegari S. Cobbold S. Cotton T. Mlodzianoski M. Schubert A. et al. (2022). Activation mechanism of PINK1. *Nature* 602 328–335. 10.1038/s41586-021-04340-2 34933320 PMC8828467

[B14] Gan Z. Callegari S. Nguyen T. Kirk N. Leis A. Lazarou M. et al. (2024). Interaction of PINK1 with nucleotides and kinetin. *Sci. Adv.* 10:eadj7408. 10.1126/sciadv.adj7408 38241364 PMC10798554

[B15] Ganley I. (2022). Strengthening the link between mitophagy and Parkinson’s disease. *Brain* 145 4154–4156. 10.1093/brain/awac405 36319596 PMC9762929

[B16] Gao X. Yang T. Gu Y. Sun X. (2022). Mitochondrial dysfunction in Parkinson’s disease: From mechanistic insights to therapy. *Front. Aging Neurosci*. 14:885500. 10.3389/fnagi.2022.885500 35795234 PMC9250984

[B17] Gladkova C. Schubert A. Wagstaff J. Pruneda J. Freund S. Komander D. (2017). An invisible ubiquitin conformation is required for efficient phosphorylation by PINK1. *EMBO J.* 36 3555–3572. 10.15252/embj.201797876 29133469 PMC5730886

[B18] Guo J. Yao L. Sun Q. Cui Y. Yang Y. Xu Q. et al. (2017). Identification of Ser465 as a novel PINK1 autophosphorylation site. *Transl. Neurodegener.* 6:34. 10.1186/s40035-017-0103-7 29255601 PMC5729251

[B19] Henrich M. Oertel W. Surmeier D. Geibl F. (2023). Mitochondrial dysfunction in Parkinson’s disease - a key disease hallmark with therapeutic potential. *Mol. Neurodegener.* 18:83. 10.1186/s13024-023-00676-7 37951933 PMC10640762

[B20] Honorato R. Trellet M. Jiménez-García B. Schaarschmidt J. Giulini M. Reys V. et al. (2024). The HADDOCK2.4 web server for integrative modeling of biomolecular complexes. *Nat. Protoc.* 19 3219–3241. 10.1038/s41596-024-01011-0 38886530

[B21] Hornbeck P. Zhang B. Murray B. Kornhauser J. Latham V. Skrzypek E. (2015). PhosphoSitePlus, 2014: Mutations, PTMs and recalibrations. *Nucleic Acids Res.* 43 D512–D520. 10.1093/nar/gku1267 25514926 PMC4383998

[B22] Hu C. Su T. Zhang Y. Yang J. Su X. Zhang Z. et al. (2025). Estradiol alleviates disease phenotypes caused by m.3635G > A mutation by activating mitochondrial biogenesis and PINK1-Parkin mediated mitophagy in iPSC-derived retinal pigment epithelium cells. *Cell. Signal.* 136:112121. 10.1016/j.cellsig.2025.112121 40930474

[B23] Huang J. MacKerell A. (2013). CHARMM36 all-atom additive protein force field: Validation based on comparison to NMR data. *J. Comput. Chem.* 34 2135–2145. 10.1002/jcc.23354 23832629 PMC3800559

[B24] Iakoucheva L. Radivojac P. Brown C. O’Connor T. Sikes J. Obradovic Z. et al. (2004). The importance of intrinsic disorder for protein phosphorylation. *Nucleic Acids Res*. 32 1037–1049. 10.1093/nar/gkh253 14960716 PMC373391

[B25] Ittisoponpisan S. Islam S. Khanna T. Alhuzimi E. David A. Sternberg M. (2019). Can predicted protein 3D structures provide reliable insights into whether missense variants are disease associated? *J. Mol. Biol.* 431 2197–2212. 10.1016/j.jmb.2019.04.009 30995449 PMC6544567

[B26] Janezic S. Threlfell S. Dodson P. Dowie M. Taylor T. Potgieter D. et al. (2013). Deficits in dopaminergic transmission precede neuron loss and dysfunction in a new Parkinson model. *Proc. Natl. Acad. Sci. U S A.* 110 E4016–E4025. 10.1073/pnas.1309143110 24082145 PMC3801069

[B27] Jendele L. Krivak R. Skoda P. Novotny M. Hoksza D. (2019). PrankWeb: A web server for ligand binding site prediction and visualization. *Nucleic Acids Res.* 47 W345–W349. 10.1093/nar/gkz424 31114880 PMC6602436

[B28] John P. Sudandiradoss C. (2025). Neurogenic locus notch homolog protein 1 (NOTCH 1) SNP informatics coupled with intrinsically disordered regions and post-translational modifications reveals the complex structural crosstalk of Lung Adenocarcinoma (LUAD). *Front. Bioinform.* 5:1641521. 10.3389/fbinf.2025.1641521 41451346 PMC12727990

[B29] Kane L. Lazarou M. Fogel A. Li Y. Yamano K. Sarraf S. et al. (2014). PINK1 phosphorylates ubiquitin to activate Parkin E3 ubiquitin ligase activity. *J. Cell. Biol.* 205 143–153. 10.1083/jcb.201402104 24751536 PMC4003245

[B30] Kazlauskaite A. Kondapalli C. Gourlay R. Campbell D. Ritorto M. Hofmann K. et al. (2014). Parkin is activated by PINK1-dependent phosphorylation of ubiquitin at Ser65. *Biochem. J.* 460 127–139. 10.1042/BJ20140334 24660806 PMC4000136

[B31] Kitada T. Asakawa S. Hattori N. Matsumine H. Yamamura Y. Minoshima S. et al. (1998). Mutations in the parkin gene cause autosomal recessive juvenile parkinsonism. *Nature* 392 605–608. 10.1038/33416 9560156

[B32] Kumar A. Tamjar J. Waddell A. Woodroof H. Raimi O. Shaw A. et al. (2017). Structure of PINK1 and mechanisms of Parkinson’s disease-associated mutations. *Elife* 6:e29985. 10.7554/eLife.29985 28980524 PMC5679756

[B33] La Sala G. Riccardi L. Gaspari R. Cavalli A. Hantschel O. De Vivo M. (2016). HRD motif as the central hub of the signaling network for activation loop autophosphorylation in Abl kinase. *J. Chem. Theory Comput.* 12 5563–5574. 10.1021/acs.jctc.6b00600 27682200

[B34] Lambourne O. Bell S. Wilhelm L. Yarbrough E. Holly G. Russell O. et al. (2023). PINK1-dependent mitophagy inhibits elevated ubiquitin phosphorylation caused by mitochondrial damage. *J. Med. Chem*. 66 7645–7656. 10.1021/acs.jmedchem.3c00555 37248632 PMC10258795

[B35] Laskowski R. Swindells M. (2011). LigPlot+: Multiple ligand-protein interaction diagrams for drug discovery. *J. Chem. Inf. Model.* 51 2778–2786. 10.1021/ci200227u 21919503

[B36] Lazarou M. Jin S. Kane L. Youle R. (2012). Role of PINK1 binding to the TOM complex and alternate intracellular membranes in recruitment and activation of the E3 ligase Parkin. *Dev. Cell.* 22 320–333. 10.1016/j.devcel.2011.12.014 22280891 PMC3288275

[B37] Li Y. Lv Z. Dai Y. Yu L. Zhang K. Wang P. (2025). The global, regional, and National burden of parkinson’s disease in 204 countries and territories, 1990–2021: A systematic analysis for the global burden of disease study 2021. *BMC Public Health* 25:3047. 10.1186/s12889-025-23492-8 40940662 PMC12427116

[B38] Luo Q. Yang X. Yao Y. Li H. Wang Y. (2014). T313M polymorphism of the PINK1 gene in Parkinson’s disease. *Exp. Ther. Med.* 8 286–290. 10.3892/etm.2014.1702 24944636 PMC4061194

[B39] McWilliams T. Barini E. Pohjolan-Pirhonen R. Brooks S. Singh F. Burel S. et al. (2018). Phosphorylation of Parkin at serine 65 is essential for its activation in vivo. *Open Biol*. 8:180108. 10.1098/rsob.180108 30404819 PMC6282074

[B40] Moriwaki Y. Kim Y. Ido Y. Misawa H. Kawashima K. Endo S. et al. (2008). L347P PINK1 mutant that fails to bind to Hsp90/Cdc37 chaperones is rapidly degraded in a proteasome-dependent manner. *Neurosci. Res.* 61 43–48. 10.1016/j.neures.2008.01.006 18359116

[B41] Okatsu K. Sato Y. Yamano K. Matsuda N. Negishi L. Takahashi A. et al. (2018). Structural insights into ubiquitin phosphorylation by PINK1. *Sci. Rep.* 8:10382. 10.1038/s41598-018-28656-8 29991771 PMC6039469

[B42] Olszewska D. McCarthy A. Soto-Beasley A. Walton R. Ross O. Lynch T. P. A. R. K. I. N. (2022). PINK1, and DJ1 analysis in early-onset Parkinson’s disease in Ireland. *Ir. J. Med. Sci.* 191 901–907. 10.1007/s11845-021-02563-w 33751372 PMC9376961

[B43] Parthiban V. Gromiha M. Schomburg D. (2006). CUPSAT: Prediction of protein stability upon point mutations. *Nucleic Acids Res*. 34 W239–W242. 10.1093/nar/gkl190 16845001 PMC1538884

[B44] Rrustemi T. Meyer K. Roske Y. Uyar B. Akalin A. Imami K. et al. (2024). Pathogenic mutations of human phosphorylation sites affect protein-protein interactions. *Nat. Commun.* 15:3146. 10.1038/s41467-024-46794-8 38605029 PMC11009412

[B45] Sim N. Kumar P. Hu J. Henikoff S. Schneider G. Ng P. C. S. I. F. T. (2012). web server: Predicting effects of amino acid substitutions on proteins. *Nucleic Acids Res*. 40 W452–W457. 10.1093/nar/gks539 22689647 PMC3394338

[B46] Simossis V. Heringa J. (2005). PRALINE: A multiple sequence alignment toolbox that integrates homology-extended and secondary structure information. *Nucleic Acids Res*. 33 W289–W294. 10.1093/nar/gki390 15980472 PMC1160151

[B47] Strong T. Kaur G. Thomas J. (2011). Mutations in the catalytic loop HRD motif alter the activity and function of Drosophila Src64. *PLoS One* 6:e28100. 10.1371/journal.pone.0028100 22132220 PMC3223231

[B48] Surmeier D. (2018). Determinants of dopaminergic neuron loss in Parkinson’s disease. *FEBS J*. 285 3657–3668. 10.1111/febs.14607 30028088 PMC6546423

[B49] Valente E. Abou-Sleiman P. Caputo V. Muqit M. Harvey K. Gispert S. et al. (2004). Hereditary early-onset Parkinson’s disease caused by mutations in PINK1. *Science* 304 1158–1160. 10.1126/science.1096284 15087508

[B50] Vives-Bauza C. Zhou C. Huang Y. Cui M. de Vries R. Kim J. et al. (2010). PINK1-dependent recruitment of Parkin to mitochondria in mitophagy. *Proc. Natl. Acad. Sci. U S A.* 107 378–383. 10.1073/pnas.0911187107 19966284 PMC2806779

[B51] Woodroof H. Pogson J. Begley M. Cantley L. Deak M. Campbell D. et al. (2011). Discovery of catalytically active orthologues of the Parkinson’s disease kinase PINK1: Analysis of substrate specificity and impact of mutations. *Open Biol.* 1:110012. 10.1098/rsob.110012 22645651 PMC3352081

[B52] Yang Y. Gehrke S. Imai Y. Huang Z. Ouyang Y. Wang J. et al. (2006). Mitochondrial pathology and muscle and dopaminergic neuron degeneration caused by inactivation of Drosophila Pink1 is rescued by Parkin. *Proc. Natl. Acad. Sci. U S A.* 103 10793–10798. 10.1073/pnas.0602493103 16818890 PMC1502310

[B53] Yin E. Dieriks B. (2025). Rethinking ‘rare’ PINK1 Parkinson’s disease: A meta-analysis of geographical prevalence, phenotypic diversity, and α-synuclein pathology. *J. Parkinsons Dis.* 15 41–65. 10.1177/1877718X241304814 39973502 PMC13347432

[B54] Zhou C. Huang Y. Shao Y. May J. Prou D. Perier C. et al. (2008). The kinase domain of mitochondrial PINK1 faces the cytoplasm. *Proc. Natl. Acad. Sci. U S A.* 105 12022–12027. 10.1073/pnas.0802814105 18687899 PMC2575334

