# Supplementary material for: Dynamic structural profiling of PINK1 mutations (T313M and L347P) reveals a vital molecular perturbation namely phospho-Serine 65 ubiquitin recognition point in mitophagy mediated autosomal recessive Parkinson’s disease (ARPD)
Source: Front Mol Neurosci. 2026 Jul 31;19:1875988. doi: 10.3389/fnmol.2026.1875988 (PMC13473279; doi:10.3389/fnmol.2026.1875988)
Supplement: Supplementary file 1 [file Data_Sheet_1.docx]

**Supplementary Fig1.** Ramachandran plots are shown for six residue categories: General case, Isoleucine and valine, Pre-proline, Glycine, Trans-proline, and Cis-proline. Residues falling within favored regions are represented as filled circles, while outliers are highlighted in pink/red with residue labels. The analysis shows that 89.5% (518/579) of residues fall in favored (≥98%) regions and 97.1% (562/579) in allowed (≥99.8%) regions, confirming overall good stereochemical quality of the predicted model. A total of 17 outliers were identified, including Ala28, Tyr29, Pro34, Pro36, Val41, Arg42, Pro46, Ala49, Arg58, Asn65, Cys92, Lys186, Thr185, Val184, Gly189, Glu203, and Lys262, predominantly located in loop or disordered regions. These results validate the structural reliability of the Human PINK1 model.

**Supplementary Fig 2.** Stepwise workflow for filtering and prioritizing PINK1 missense variants. A total of 825 missense variants (UniProt: Q9BXM7) were functionally annotated using Ensembl VEP and independently assessed by SIFT and PolyPhen-2. Variants predicted as deleterious by both tools (SIFT ≤ 0.05 and PolyPhen-2 ≥ 0.90) were retained using an AND criterion (n = 276). These variants were further filtered using ClinVar records, clinical significance, and review status to identify the final prioritized variants for structural and molecular dynamics analyses.

**Supplementary Fig 3.** Principal component analysis (PCA) and dynamic cross-correlation matrix (DCCM) analysis of WT_PINK1–Ub, T313M_PINK1–Ub, and L347P_PINK1–Ub complexes (Replicate 2). (A–C) Free-energy landscapes (FELs) generated using the first two principal components (PC1 and PC2) for WT_PINK1–Ub, T313M_PINK1–Ub, and L347P_PINK1–Ub systems, respectively. Regions of low free energy correspond to energetically favorable conformational states sampled during the simulation. (D–F) Dynamic cross-correlation matrices illustrating correlated and anti-correlated residue motions within WT_PINK1–Ub, T313M_PINK1–Ub, and L347P_PINK1–Ub complexes. Positive correlations indicate concerted residue movements, whereas negative correlations represent opposing motions.

**Supplementary Fig 4.** Principal component analysis (PCA) and dynamic cross-correlation matrix (DCCM) analysis of WT_PINK1–Ub, T313M_PINK1–Ub, and L347P_PINK1–Ub complexes (Replicate 3). (A–C) Free-energy landscapes projected along the first two principal components for WT_PINK1–Ub, T313M_PINK1–Ub, and L347P_PINK1–Ub systems. (D–F) Corresponding DCCM maps showing mutation-associated alterations in residue communication networks and collective motions across the protein complexes.

**Supplementary Fig 5.** Intermolecular interaction analysis of WT_PINK1–Ub and mutant complexes across three independent simulation replicates. (A–C) Time-dependent profiles of hydrogen bonds, hydrophobic interactions, and salt bridges observed between PINK1 and ubiquitin in WT_PINK1–Ub, T313M_PINK1–Ub, and L347P_PINK1–Ub complexes during replicates 1, 2, and 3, respectively. The analysis was performed to evaluate the stability and persistence of key intermolecular interactions contributing to complex formation and maintenance throughout the molecular dynamics simulations.

**Supplementary Table 1.** Stepwise filtering pipeline used to prioritize PINK1 missense variants. Starting from 825 missense variants (UniProt: Q9BXM7), variants were filtered using SIFT (≤ 0.05) and PolyPhen-2 (≥ 0.90), with the intersection (AND) yielding 276 high-confidence deleterious variants. Subsequent filtering based on ClinVar records, clinical significance, and review status identified five variants for downstream analyses.

| **Step No.** | **Filtering step/ Criteria** | **Tool /Database** | **Variants remaining (n)** | **Description** |
| --- | --- | --- | --- | --- |
| 1. | Total missense variants in Human PINK1 (Q9BXM7) | Ensembl/ UniProt | 825 | Initial Dataset |
| 2. | Functional annotation (Transcript aggregation) | Ensembl VEP | 825 | Max. PolyPhen-2 score and min SIFT score retained per variant |
| 3a. | SIFT Prediction (Score ≤ 0.05) | SIFT | 412 | Predicted deleterious by SIFT |
| 3b. | PolyPhen-2 prediction (score ≥ 0.90) | PolyPhen-2 | 524 | Predicted damaging by PolyPhen-2 |
| 4. | Intersection (AND) SIFT ≤ 0.05 AND PolyPhen-2 ≥ 0.90 | SIFT ∩ PolyPhen-2 (AND) | 276 | High confidence deleterious variants (intersection) |
| 5. | Variants with ClinVar Records | ClinVar | 203 | ClinVar associated |
| 6. | Clinical Significance | ClinVar | 20 | Variants with clinical significance |
| 7. | Review status filter (Criteria provided) | ClinVar | 5 | Single submitter or multiple submitter with no conflicts |

**Supplementary Table 2**

This table lists a total 824 missense variants identified by reference SNP ID (rsID) together with the corresponding amino acid substitution and the predicted functional effect from two complementary in silico prediction algorithms, PolyPhen-2 and SIFT. PolyPhen-2 scores range from 0 (benign) to 1 (probably damaging); SIFT scores range from 0 (deleterious) to 1 (tolerated), with a standard threshold of 0.05 distinguishing deleterious from tolerated substitutions. Entries listed as “NA” indicate that a prediction score was not available for that variant/algorithm.

| **rs_id** | **amino_acid_substitution** | **polyphen_score** | **polyphen_prediction** | **sift_score** | **sift_prediction** |
| --- | --- | --- | --- | --- | --- |
| rs1043424 | N521T | 0.006 | benign | 0.33 | tolerated |
| rs1043424 | N521S | 0.006 | benign | 0.33 | tolerated |
| rs3738136 | A340T | 0.001 | benign | 1 | tolerated |
| rs28940284 | H271Q | 0.984 | probably_damaging | 0 | deleterious |
| rs28940285 | L347P | 1 | probably_damaging | 0 | deleterious |
| rs34203620 | C377F | 0.992 | probably_damaging | 0.02 | deleterious |
| rs34677717 | P209R | 0.038 | benign | 0.05 | deleterious |
| rs34677717 | P209L | 0.038 | benign | 0.05 | deleterious |
| rs35813094 | M341I | 0.035 | benign | 0.03 | deleterious |
| rs45478900 | G411S | 0.018 | benign | 0.36 | tolerated |
| rs45515602 | A383T | 0.007 | benign | 0 | deleterious_low_confidence |
| rs45515602 | A383S | 0.007 | benign | 0 | deleterious_low_confidence |
| rs45604240 | T145M | 0.01 | benign | 0.02 | deleterious |
| rs45608139 | R152W | 0.645 | possibly_damaging | 0 | deleterious |
| rs55831733 | A339T | 0.606 | possibly_damaging | 0 | deleterious |
| rs56217826 | K496N | NA | NA | NA | NA |
| rs61735932 | R279C | 0.987 | probably_damaging | 0 | deleterious |
| rs61744200 | R501Q | 0.997 | probably_damaging | 0 | deleterious |
| rs61744200 | R501P | 0.997 | probably_damaging | 0 | deleterious |
| rs74315355 | G309D | 1 | probably_damaging | 0 | deleterious |
| rs74315358 | R279H | 0.724 | possibly_damaging | 0 | deleterious |
| rs74315359 | T313M | 0.992 | probably_damaging | 0 | deleterious |
| rs74315360 | A217D | 0.985 | probably_damaging | 0 | deleterious |
| rs74315361 | Y431H | 0.996 | probably_damaging | 0 | deleterious |
| rs76753586 | A359T | 1 | probably_damaging | 0 | deleterious |
| rs112600292 | P305A | 0.98 | probably_damaging | 0 | deleterious |
| rs113092523 | S284Y | 0.888 | possibly_damaging | 0 | deleterious |
| rs113092523 | S284C | 0.888 | possibly_damaging | 0 | deleterious |
| rs113092523 | S284F | 0.888 | possibly_damaging | 0 | deleterious |
| rs115477764 | E476K | 0.011 | benign | 0.34 | tolerated |
| rs115477764 | E476Q | 0.011 | benign | 0.34 | tolerated |
| rs119451946 | P399L | 0.977 | probably_damaging | 0 | deleterious |
| rs138302371 | P196L | 0.132 | benign | 0.03 | deleterious |
| rs139226733 | M318V | 0.636 | possibly_damaging | 0 | deleterious |
| rs139226733 | M318L | 0.636 | possibly_damaging | 0 | deleterious |
| rs141363209 | H515R | 0 | benign | 0.07 | tolerated |
| rs143204084 | K186N | 0.151 | benign | 0.08 | tolerated |
| rs143620063 | R492Q | 0.982 | probably_damaging | 0.01 | deleterious |
| rs143620063 | R492L | 0.982 | probably_damaging | 0.01 | deleterious |
| rs144071530 | A232V | 0.974 | probably_damaging | 0 | deleterious |
| rs144330127 | S335R | 0.043 | benign | 0.16 | tolerated |
| rs144330127 | S335G | 0.043 | benign | 0.16 | tolerated |
| rs144440543 | L461R | 0.992 | probably_damaging | 0 | deleterious |
| rs145650643 | L249V | 0.809 | possibly_damaging | 0.02 | deleterious_low_confidence |
| rs145876364 | R407W | 0.983 | probably_damaging | 0 | deleterious |
| rs146691996 | P286Q | 0.983 | probably_damaging | 0.01 | deleterious |
| rs146691996 | P286R | 0.983 | probably_damaging | 0.01 | deleterious |
| rs146691996 | P286L | 0.983 | probably_damaging | 0.01 | deleterious |
| rs148871409 | Q115P | 0.857 | possibly_damaging | 0.04 | deleterious |
| rs148871409 | Q115L | 0.857 | possibly_damaging | 0.04 | deleterious |
| rs149822257 | A200V | 0.027 | benign | 0.12 | tolerated |
| rs150964528 | Q456R | 0.145 | benign | 0.2 | tolerated |
| rs184043309 | R337H | 0.101 | benign | 0.07 | tolerated |
| rs200729715 | D366N | 0.975 | probably_damaging | 0.01 | deleterious |
| rs200949139 | V317I | 0.66 | possibly_damaging | 0.01 | deleterious |
| rs200949139 | V317L | 0.66 | possibly_damaging | 0.01 | deleterious |
| rs201491485 | R422H | 0 | benign | 0.13 | tolerated |
| rs201940147 | R302H | 0.045 | benign | 0 | deleterious |
| rs201940147 | R302L | 0.045 | benign | 0 | deleterious |
| rs202048763 | S73L | 0.991 | probably_damaging | 0 | deleterious |
| rs202128685 | R312Q | 0.874 | possibly_damaging | 0 | deleterious |
| rs370906995 | T257S | 0.006 | benign | 0.02 | deleterious_low_confidence |
| rs370906995 | T257I | 0.006 | benign | 0.02 | deleterious_low_confidence |
| rs371338035 | K526E | 0.003 | benign | 0.02 | deleterious |
| rs371385940 | K186Q | 0.007 | benign | 0.11 | tolerated |
| rs371385940 | K186E | 0.007 | benign | 0.11 | tolerated |
| rs371854396 | P215R | 0.989 | probably_damaging | 0 | deleterious |
| rs371854396 | P215L | 0.989 | probably_damaging | 0 | deleterious |
| rs372280083 | L268V | 0.723 | possibly_damaging | 0.14 | tolerated |
| rs373417787 | C323Y | 0.017 | benign | 0.08 | tolerated |
| rs373417787 | C323S | 0.017 | benign | 0.08 | tolerated |
| rs374167096 | R422C | 0 | benign | 0.2 | tolerated |
| rs374891130 | T133N | 0.203 | benign | 0.12 | tolerated |
| rs374891130 | T133I | 0.203 | benign | 0.12 | tolerated |
| rs375601968 | R246Q | 0.408 | benign | 0.31 | tolerated |
| rs376829940 | D481V | 0.936 | probably_damaging | 0 | deleterious |
| rs398124659 | L308Q | 0.285 | benign | 0 | deleterious |
| rs531477772 | D525N | 0.113 | benign | 0.06 | tolerated |
| rs531477772 | D525Y | 0.113 | benign | 0.06 | tolerated |
| rs542258150 | A494D | 0.015 | benign | 0.01 | deleterious |
| rs542258150 | A494V | 0.015 | benign | 0.01 | deleterious |
| rs543071128 | G105E | 0.866 | possibly_damaging | 0 | deleterious |
| rs543071128 | G105A | 0.866 | possibly_damaging | 0 | deleterious |
| rs543071128 | G105V | 0.866 | possibly_damaging | 0 | deleterious |
| rs544457754 | I275V | 0.127 | benign | 0.07 | tolerated |
| rs548506734 | R276Q | 0.802 | possibly_damaging | 0 | deleterious |
| rs548506734 | R276L | 0.802 | possibly_damaging | 0 | deleterious |
| rs551542832 | G23S | 0.019 | benign | 0.19 | tolerated_low_confidence |
| rs553247784 | R66S | 0.997 | probably_damaging | 0 | deleterious |
| rs553247784 | R66G | 0.997 | probably_damaging | 0 | deleterious |
| rs553247784 | R66C | 0.997 | probably_damaging | 0 | deleterious |
| rs554114655 | P425S | 0.553 | possibly_damaging | 0.04 | deleterious |
| rs556540177 | R407Q | 0.978 | probably_damaging | 0 | deleterious |
| rs556540177 | R407P | 0.978 | probably_damaging | 0 | deleterious |
| rs556540177 | R407L | 0.978 | probably_damaging | 0 | deleterious |
| rs557503577 | V285M | 0.857 | possibly_damaging | 0 | deleterious |
| rs557503577 | V285L | 0.857 | possibly_damaging | 0 | deleterious |
| rs569753606 | G30S | 0.34 | benign | 0 | deleterious_low_confidence |
| rs569753606 | G30R | 0.34 | benign | 0 | deleterious_low_confidence |
| rs570833319 | S301L | 0 | benign | 0.09 | tolerated |
| rs746940423 | G47S | 0.973 | probably_damaging | 0 | deleterious_low_confidence |
| rs746940423 | G47R | 0.973 | probably_damaging | 0 | deleterious_low_confidence |
| rs746940423 | G47C | 0.973 | probably_damaging | 0 | deleterious_low_confidence |
| rs747133533 | W577R | 0.816 | possibly_damaging | 0 | deleterious |
| rs747400197 | N451S | 0.996 | probably_damaging | 0 | deleterious |
| rs747400197 | N451I | 0.996 | probably_damaging | 0 | deleterious |
| rs748041977 | R337G | 0.966 | probably_damaging | 0 | deleterious |
| rs748041977 | R337C | 0.966 | probably_damaging | 0 | deleterious |
| rs749456905 | Q115H | 0.924 | probably_damaging | 0.06 | tolerated |
| rs751012180 | M527T | 0.171 | benign | 0 | deleterious_low_confidence |
| rs751012180 | M527R | 0.171 | benign | 0 | deleterious_low_confidence |
| rs752317048 | E563D | 0.046 | benign | 0.38 | tolerated_low_confidence |
| rs753186891 | M342L | 0.965 | probably_damaging | 0 | deleterious |
| rs753186891 | M342V | 0.965 | probably_damaging | 0 | deleterious |
| rs753196052 | I158V | 0.665 | possibly_damaging | 0.01 | deleterious |
| rs755060576 | Y321C | 0.999 | probably_damaging | 0 | deleterious |
| rs755060576 | Y321F | 0.999 | probably_damaging | 0 | deleterious |
| rs757581951 | G189R | 0 | benign | 0.19 | tolerated |
| rs759468742 | R207Q | 0 | benign | 0.15 | tolerated |
| rs759468742 | R207L | 0 | benign | 0.15 | tolerated |
| rs759968468 | G62R | 0.001 | benign | 0.07 | tolerated_low_confidence |
| rs762698585 | F104C | 0.716 | possibly_damaging | 0 | deleterious |
| rs763142730 | L67F | 0 | benign | 0.04 | deleterious |
| rs763416852 | F385L | 0.978 | probably_damaging | 0 | deleterious |
| rs763416852 | F385V | 0.978 | probably_damaging | 0 | deleterious |
| rs764328076 | R464H | 0.995 | probably_damaging | 0.01 | deleterious |
| rs764454206 | K520N | 0.265 | benign | 0.03 | deleterious |
| rs768091663 | A168T | 0.998 | probably_damaging | 0 | deleterious |
| rs768091663 | A168P | 0.998 | probably_damaging | 0 | deleterious |
| rs770790488 | E206K | 0.041 | benign | 0.29 | tolerated |
| rs771032673 | A537T | 0.66 | possibly_damaging | 0.01 | deleterious_low_confidence |
| rs772510148 | A280T | 0.809 | possibly_damaging | 0 | deleterious |
| rs772774256 | G424D | 0.595 | possibly_damaging | 0 | deleterious |
| rs775160272 | P142R | 0.966 | probably_damaging | 0.01 | deleterious |
| rs775160272 | P142L | 0.966 | probably_damaging | 0.01 | deleterious |
| rs775165635 | M237V | 0.482 | possibly_damaging | 0 | deleterious |
| rs775809722 | Q126P | 0.138 | benign | 0 | deleterious |
| rs775809722 | Q126R | 0.138 | benign | 0 | deleterious |
| rs775809722 | Q126L | 0.138 | benign | 0 | deleterious |
| rs776134109 | Y171C | 0.999 | probably_damaging | 0 | deleterious |
| rs776293086 | P52Q | 0.007 | benign | 0.01 | deleterious_low_confidence |
| rs776293086 | P52L | 0.007 | benign | 0.01 | deleterious_low_confidence |
| rs777088052 | P289T | 1 | probably_damaging | 0 | deleterious |
| rs777146957 | Q129E | 0.007 | benign | 0 | deleterious |
| rs777439010 | E392D | 0.026 | benign | 0.14 | tolerated |
| rs777895210 | V418M | 0.998 | probably_damaging | 0 | deleterious |
| rs777895210 | V418L | 0.998 | probably_damaging | 0 | deleterious |
| rs777895210 | V418L | 0.998 | probably_damaging | 0 | deleterious |
| rs778009684 | R276W | 0.991 | probably_damaging | 0 | deleterious |
| rs779060308 | P296R | 1 | probably_damaging | 0 | deleterious |
| rs779060308 | P296L | 1 | probably_damaging | 0 | deleterious |
| rs779294259 | A504T | 1 | probably_damaging | 0 | deleterious |
| rs779798626 | M527V | 0.003 | benign | 0.1 | tolerated |
| rs780971409 | A269V | 0.365 | benign | 0.01 | deleterious |
| rs781745700 | P138Q | 0.001 | benign | 0.25 | tolerated |
| rs781745700 | P138R | 0.001 | benign | 0.25 | tolerated |
| rs781745700 | P138L | 0.001 | benign | 0.25 | tolerated |
| rs867771554 | A54E | 0.116 | benign | 0 | deleterious |
| rs867771554 | A54G | 0.116 | benign | 0 | deleterious |
| rs886044686 | L11M | 0 | unknown | 0 | deleterious_low_confidence |
| rs886045848 | K526N | 0.229 | benign | 0.03 | deleterious |
| rs889189102 | R147C | 0.011 | benign | 0.03 | deleterious_low_confidence |
| rs897203855 | A16E | 0 | unknown | 0 | deleterious |
| rs897203855 | A16G | 0 | unknown | 0 | deleterious |
| rs897203855 | A16V | 0 | unknown | 0 | deleterious |
| rs956491558 | R58K | 0.035 | benign | 0.05 | deleterious |
| rs956491558 | R58T | 0.035 | benign | 0.05 | deleterious |
| rs1005937012 | Q5E | NA | NA | NA | NA |
| rs1006830855 | P34R | 0.076 | benign | 0 | deleterious_low_confidence |
| rs1006830855 | P34L | 0.076 | benign | 0 | deleterious_low_confidence |
| rs1057519104 | R57C | 0 | benign | 0.02 | deleterious |
| rs1159680235 | P212S | 0.005 | benign | 0.22 | tolerated |
| rs1178309546 | T333I | 0.365 | benign | 0.11 | tolerated |
| rs1200609271 | P374R | 0.223 | benign | 0.01 | deleterious |
| rs1200609271 | P374L | 0.223 | benign | 0.01 | deleterious |
| rs1207717010 | A75S | 0.003 | benign | 0.45 | tolerated |
| rs1252036117 | G163D | 0.999 | probably_damaging | 0 | deleterious |
| rs1255530845 | R42C | 0.343 | benign | 0 | deleterious |
| rs1265322947 | A536S | 0.998 | probably_damaging | 0 | deleterious |
| rs1274588239 | A124G | 0.56 | possibly_damaging | 0 | deleterious |
| rs1274588239 | A124V | 0.56 | possibly_damaging | 0 | deleterious |
| rs1275557772 | A38V | 0.039 | benign | 0.01 | deleterious_low_confidence |
| rs1285969593 | G395S | 0.729 | possibly_damaging | 0.19 | tolerated |
| rs1310430648 | E252K | 0.689 | possibly_damaging | 0.01 | deleterious |
| rs1355118616 | A38T | 0.019 | benign | 0.01 | deleterious_low_confidence |
| rs1355386291 | S393R | 0.007 | benign | 0.6 | tolerated |
| rs1390551264 | R68C | 0.716 | possibly_damaging | 0 | deleterious |
| rs1432225013 | R501G | NA | NA | 0 | deleterious |
| rs1433292317 | V122I | 0.007 | benign | 0.04 | deleterious |
| rs1433292317 | V122L | 0.007 | benign | 0.04 | deleterious |
| rs1453237796 | P336A | 0.204 | benign | 0.49 | tolerated |
| rs1456150277 | L81M | 0.631 | possibly_damaging | 0.01 | deleterious |
| rs1456585417 | N223S | 0.496 | possibly_damaging | 0 | deleterious |
| rs1456585417 | N223I | 0.496 | possibly_damaging | 0 | deleterious |
| rs1553146806 | C388R | 1 | probably_damaging | 0 | deleterious |
| rs1570405073 | V370M | 0.913 | probably_damaging | 0.03 | deleterious |
| rs1570407259 | L396R | 0.991 | probably_damaging | 0 | deleterious |
| rs1570409378 | Q534K | 0.502 | possibly_damaging | 0.01 | deleterious |
| rs2053014865 | F21L | 0.833 | possibly_damaging | 0.09 | tolerated_low_confidence |
| rs2053015141 | G26R | 0.647 | possibly_damaging | 0.02 | deleterious |
| rs2053015980 | G39D | 0.003 | benign | 0.01 | deleterious_low_confidence |
| rs2053015980 | G39V | 0.003 | benign | 0.01 | deleterious_low_confidence |
| rs2053021240 | L110I | 0.778 | possibly_damaging | 0 | deleterious |
| rs2053021240 | L110V | 0.778 | possibly_damaging | 0 | deleterious |
| rs2053021240 | L110F | 0.778 | possibly_damaging | 0 | deleterious |
| rs2053073220 | G150S | 1 | probably_damaging | 0 | deleterious |
| rs2053073220 | G150C | 1 | probably_damaging | 0 | deleterious |
| rs2053073251 | G150D | 0.999 | probably_damaging | 0 | deleterious |
| rs2053073251 | G150A | 0.999 | probably_damaging | 0 | deleterious |
| rs2053151793 | A269T | 0.047 | benign | 0.01 | deleterious_low_confidence |
| rs2053171495 | N367H | 1 | probably_damaging | 0 | deleterious |
| rs2053171495 | N367D | 1 | probably_damaging | 0 | deleterious |
| rs2053171495 | N367Y | 1 | probably_damaging | 0 | deleterious |
| rs2053215939 | C377W | 0.996 | probably_damaging | 0 | deleterious |
| rs2545244807 | T22M | 0.53 | possibly_damaging | 0.1 | tolerated_low_confidence |
| rs2545244881 | Y29D | 0.028 | benign | 0.02 | deleterious |
| rs2545244892 | L31V | 0.036 | benign | 0.07 | tolerated |
| rs2545245029 | G53D | 0.711 | possibly_damaging | 0.01 | deleterious |
| rs2545245366 | R88Q | 0.994 | probably_damaging | 0 | deleterious |
| rs2545245366 | R88L | 0.994 | probably_damaging | 0 | deleterious |
| rs2545245426 | C96W | 0.895 | possibly_damaging | 0.02 | deleterious |
| rs2545250336 | M221T | 0.921 | probably_damaging | 0 | deleterious |
| rs2545258128 | K262N | 0.129 | benign | 0.02 | deleterious_low_confidence |
| rs2545258132 | R263K | 0.055 | benign | 0.29 | tolerated |
| rs2545258365 | F315L | 0.99 | probably_damaging | 0.02 | deleterious |
| rs2545265447 | D430H | 0.985 | probably_damaging | 0 | deleterious |
| rs2545265790 | Q491P | 0.96 | probably_damaging | 0.01 | deleterious |
| rs2545267724 | A500G | 0.999 | probably_damaging | 0 | deleterious |
| rs7349186 | P305R | 0.99 | probably_damaging | 0 | deleterious |
| rs7349186 | P305L | 0.99 | probably_damaging | 0 | deleterious |
| rs17852513 | P209A | 0.001 | benign | 0.08 | tolerated |
| rs17852513 | P209S | 0.001 | benign | 0.08 | tolerated |
| rs34416410 | S477T | 0.011 | benign | 0.31 | tolerated |
| rs35802484 | P196S | 0.132 | benign | 0.08 | tolerated |
| rs45467995 | G440E | 1 | probably_damaging | 0 | deleterious |
| rs45545034 | G227R | 0.982 | probably_damaging | 0 | deleterious |
| rs56297806 | L148S | 0 | benign | 0.04 | deleterious |
| rs56297806 | L148W | 0 | benign | 0.04 | deleterious |
| rs61749520 | A541S | 0.087 | benign | 0.42 | tolerated |
| rs138018628 | G227D | 0.875 | possibly_damaging | 0 | deleterious |
| rs138018628 | G227V | 0.875 | possibly_damaging | 0 | deleterious |
| rs138050841 | R147H | 0.011 | benign | 0.05 | tolerated |
| rs138135040 | A443V | 0.649 | possibly_damaging | 0 | deleterious |
| rs140652495 | F447L | 0.023 | benign | 0.38 | tolerated |
| rs142028165 | R326H | 0.806 | possibly_damaging | 0 | deleterious |
| rs142028165 | R326L | 0.806 | possibly_damaging | 0 | deleterious |
| rs143659957 | Y444C | 0.99 | probably_damaging | 0 | deleterious |
| rs145821082 | V405M | 0.918 | probably_damaging | 0.02 | deleterious |
| rs146126901 | S535L | 0.998 | probably_damaging | 0 | deleterious |
| rs148313557 | A443T | 0.174 | benign | 0.04 | deleterious |
| rs148313557 | A443S | 0.174 | benign | 0.04 | deleterious |
| rs181675228 | G290E | 0.997 | probably_damaging | 0 | deleterious |
| rs183454580 | A438E | 0.942 | probably_damaging | 0 | deleterious |
| rs183454580 | A438V | 0.942 | probably_damaging | 0 | deleterious |
| rs200036561 | T282S | 0.946 | probably_damaging | 0 | deleterious |
| rs200462866 | C387G | 0.923 | probably_damaging | 0 | deleterious |
| rs200728364 | S402N | 0.991 | probably_damaging | 0.02 | deleterious |
| rs201885172 | A211T | 0.006 | benign | 0.25 | tolerated |
| rs201885172 | A211S | 0.006 | benign | 0.25 | tolerated |
| rs201920474 | Q345H | 1 | probably_damaging | 0 | deleterious |
| rs202130946 | L413V | 0.999 | probably_damaging | 0 | deleterious |
| rs367628601 | Q205R | 0 | benign | 0.17 | tolerated |
| rs368216507 | L396V | 0.924 | probably_damaging | 0 | deleterious |
| rs368438636 | S432N | 0.991 | probably_damaging | 0.01 | deleterious |
| rs368939214 | S393N | 0.193 | benign | 0.23 | tolerated |
| rs368939214 | S393T | 0.193 | benign | 0.23 | tolerated |
| rs369043567 | A441T | 0.149 | benign | 0.1 | tolerated |
| rs369569239 | D141G | 0.003 | benign | 0.02 | deleterious |
| rs371775590 | K433N |  |  |  |  |
| rs373473255 | D294N | 0.955 | probably_damaging | 0 | deleterious |
| rs373473255 | D294Y | 0.955 | probably_damaging | 0 | deleterious |
| rs373657922 | Q467E | 0.355 | benign | 0.08 | tolerated |
| rs374204111 | I158M | 0.965 | probably_damaging | 0.01 | deleterious |
| rs374547622 | S199T | 0 | benign | 0.18 | tolerated |
| rs374657174 | C412W | 0.957 | probably_damaging | 0 | deleterious |
| rs374910091 | A434T | 0.719 | possibly_damaging | 0.03 | deleterious |
| rs375197494 | H310R | 0.033 | benign | 0.06 | tolerated |
| rs376323248 | R326C | 0.157 | benign | 0 | deleterious |
| rs527713210 | A217T | 0.942 | probably_damaging | 0 | deleterious |
| rs527713210 | A217S | 0.942 | probably_damaging | 0 | deleterious |
| rs536023801 | N542T | 0.16 | benign | 0.05 | tolerated_low_confidence |
| rs536023801 | N542S | 0.16 | benign | 0.05 | tolerated_low_confidence |
| rs536146282 | R312W | 0.989 | probably_damaging | 0 | deleterious |
| rs543939499 | G193R | 0.189 | benign | 0.12 | tolerated |
| rs544984102 | R83Q | 0.64 | possibly_damaging | 0.01 | deleterious |
| rs548701521 | I382M | 0.983 | probably_damaging | 0 | deleterious |
| rs552625273 | I224T | 0.901 | possibly_damaging | 0 | deleterious |
| rs552625273 | I224S | 0.901 | possibly_damaging | 0 | deleterious |
| rs554043854 | L544P | 0.926 | probably_damaging | 0 | deleterious_low_confidence |
| rs559412676 | V247M | 0.025 | benign | 0.02 | deleterious_low_confidence |
| rs559412676 | V247L | 0.025 | benign | 0.02 | deleterious_low_confidence |
| rs560377198 | A504G | 1 | probably_damaging | 0 | deleterious |
| rs560377198 | A504V | 1 | probably_damaging | 0 | deleterious |
| rs561948589 | I233V | 0.274 | benign | 0 | deleterious |
| rs563467429 | R88W | 0.999 | probably_damaging | 0 | deleterious |
| rs570488420 | G195D | 0.006 | benign | 0.12 | tolerated |
| rs573411900 | L413P | 1 | probably_damaging | 0 | deleterious |
| rs573931674 | E240K | 0.974 | probably_damaging | 0 | deleterious |
| rs574137009 | V59A | 0.003 | benign | 0.01 | deleterious_low_confidence |
| rs574137009 | V59G | 0.003 | benign | 0.01 | deleterious_low_confidence |
| rs574890623 | G409R | 0.996 | probably_damaging | 0 | deleterious |
| rs575668171 | R98G | 0.825 | possibly_damaging | 0 | deleterious |
| rs575668171 | R98W | 0.825 | possibly_damaging | 0 | deleterious |
| rs577010119 | L31F | 0.019 | benign | 0.12 | tolerated |
| rs746115592 | L574P | 0.992 | probably_damaging | 0 | deleterious |
| rs746698825 | C166Y | 0.963 | probably_damaging | 0 | deleterious |
| rs746698825 | C166S | 0.963 | probably_damaging | 0 | deleterious |
| rs746698825 | C166F | 0.963 | probably_damaging | 0 | deleterious |
| rs746882245 | S238I | 0.921 | probably_damaging | 0 | deleterious |
| rs747359904 | R302C | 0.878 | possibly_damaging | 0.02 | deleterious |
| rs747778265 | M220V | 0.552 | possibly_damaging | 0 | deleterious |
| rs747865765 | R80Q | 0.171 | benign | 0 | deleterious |
| rs747865765 | R80L | 0.171 | benign | 0 | deleterious |
| rs748103970 | Q534P | 0.933 | probably_damaging | 0 | deleterious |
| rs748103970 | Q534R | 0.933 | probably_damaging | 0 | deleterious |
| rs748343847 | A54T | 0.001 | benign | 0.1 | tolerated |
| rs748904588 | V478A | 0.846 | possibly_damaging | 0.15 | tolerated |
| rs749040285 | N367S | 1 | probably_damaging | 0 | deleterious |
| rs749040285 | N367I | 1 | probably_damaging | 0 | deleterious |
| rs749546463 | A89D | 0.148 | benign | 0.12 | tolerated |
| rs749546463 | A89V | 0.148 | benign | 0.12 | tolerated |
| rs749553312 | V293I | 0.095 | benign | 0.23 | tolerated |
| rs749553312 | V293L | 0.095 | benign | 0.23 | tolerated |
| rs749629580 | Q160P | 0.473 | possibly_damaging | 0.01 | deleterious |
| rs750332155 | R259K | 0.144 | benign | 0 | deleterious |
| rs750589610 | A473V | 0.619 | possibly_damaging | 0.07 | tolerated |
| rs751020216 | G109D | 0.999 | probably_damaging | 0 | deleterious |
| rs751259577 | L557R | 0.574 | possibly_damaging | 0.02 | deleterious |
| rs751275626 | G448R | 1 | probably_damaging | 0.01 | deleterious |
| rs751522476 | A427T | 0.015 | benign | 0 | deleterious_low_confidence |
| rs751853691 | E154G | 0.978 | probably_damaging | 0 | deleterious |
| rs752704476 | E112K | 0.487 | possibly_damaging | 0 | deleterious |
| rs752704476 | E112Q | 0.487 | possibly_damaging | 0 | deleterious |
| rs754543494 | V528A | 0.009 | benign | 0.39 | tolerated_low_confidence |
| rs754884141 | G76R | 0.999 | probably_damaging | 0 | deleterious |
| rs754928971 | L288M | 0.996 | probably_damaging | 0 | deleterious |
| rs755069256 | E155G | 0.352 | benign | 0 | deleterious |
| rs755584202 | M220I | 0.653 | possibly_damaging | 0 | deleterious |
| rs756084756 | C564Y | 0.049 | benign | 0.01 | deleterious |
| rs756084756 | C564S | 0.049 | benign | 0.01 | deleterious |
| rs756873821 | E231K | 0.66 | possibly_damaging | 0 | deleterious |
| rs757102843 | L573P | 0.972 | probably_damaging | 0 | deleterious |
| rs757253324 | I394V | 0.003 | benign | 0.57 | tolerated |
| rs757292901 | Q467P | 0.884 | possibly_damaging | 0 | deleterious |
| rs757292901 | Q467R | 0.884 | possibly_damaging | 0 | deleterious |
| rs757669987 | D351N | 0.97 | probably_damaging | 0 | deleterious |
| rs757930144 | Q72H | 0.197 | benign | 0.04 | deleterious |
| rs757930144 | Q72H | 0.197 | benign | 0.04 | deleterious |
| rs757969881 | P265T | 0.373 | benign | 0.01 | deleterious_low_confidence |
| rs759620550 | M341L | 0.003 | benign | 0.41 | tolerated |
| rs759620550 | M341V | 0.003 | benign | 0.41 | tolerated |
| rs759686620 | L369F | 1 | probably_damaging | 0 | deleterious |
| rs759723977 | G91S | 0.884 | possibly_damaging | 0 | deleterious |
| rs759723977 | G91R | 0.884 | possibly_damaging | 0 | deleterious |
| rs759723977 | G91C | 0.884 | possibly_damaging | 0 | deleterious |
| rs759849441 | V184L | 0.015 | benign | 0.3 | tolerated |
| rs759849441 | V184L | 0.015 | benign | 0.3 | tolerated |
| rs760436322 | S463G | 0.99 | probably_damaging | 0 | deleterious |
| rs760436322 | S463C | 0.99 | probably_damaging | 0 | deleterious |
| rs761085380 | S283C | 0.949 | probably_damaging | 0 | deleterious |
| rs761156393 | E546G | 0.039 | benign | 0.04 | deleterious_low_confidence |
| rs761290240 | T174A | 0 | benign | 1 | tolerated |
| rs762044569 | A580V | 0.767 | possibly_damaging | 0 | deleterious_low_confidence |
| rs762287023 | T552A | 0.106 | benign | 0.33 | tolerated_low_confidence |
| rs762760806 | Q180H | 0 | benign | 0.08 | tolerated |
| rs763000118 | V59I | 0.003 | benign | 0.26 | tolerated_low_confidence |
| rs763000118 | V59L | 0.003 | benign | 0.26 | tolerated_low_confidence |
| rs763284341 | V506M | 0.808 | possibly_damaging | 0 | deleterious_low_confidence |
| rs763628949 | G251E | 0.306 | benign | 0 | deleterious |
| rs763628949 | G251A | 0.306 | benign | 0 | deleterious |
| rs764042726 | M221V | 0.552 | possibly_damaging | 0 | deleterious |
| rs764116037 | A213D | 0.127 | benign | 0.04 | deleterious |
| rs764648391 | V350M | 0.998 | probably_damaging | 0 | deleterious |
| rs764648391 | V350L | 0.998 | probably_damaging | 0 | deleterious |
| rs764654160 | K547T | 0.28 | benign | 0.02 | deleterious_low_confidence |
| rs765900048 | L557F | 0.473 | possibly_damaging | 0.01 | deleterious |
| rs766319968 | R426G | 0.011 | benign | 0 | deleterious |
| rs766319968 | R426W | 0.011 | benign | 0 | deleterious |
| rs767633723 | A169T | 0.998 | probably_damaging | 0 | deleterious |
| rs767633723 | A169P | 0.998 | probably_damaging | 0 | deleterious |
| rs767633723 | A169S | 0.998 | probably_damaging | 0 | deleterious |
| rs767833301 | D375H | 0.878 | possibly_damaging | 0.02 | deleterious |
| rs768019187 | P322L | 0.997 | probably_damaging | 0 | deleterious |
| rs768456559 | A49T | 0.019 | benign | 0.15 | tolerated |
| rs768758677 | I382V | 0.968 | probably_damaging | 0 | deleterious |
| rs768758677 | I382F | 0.968 | probably_damaging | 0 | deleterious |
| rs769130824 | R578S | 0.997 | probably_damaging | 0 | deleterious |
| rs769395168 | G424R | 0.998 | probably_damaging | 0 | deleterious |
| rs769395168 | G424C | 0.998 | probably_damaging | 0 | deleterious |
| rs769460438 | Y454C | 0.999 | probably_damaging | 0 | deleterious |
| rs769626575 | Q82P | 0.647 | possibly_damaging | 0 | deleterious |
| rs769626575 | Q82L | 0.647 | possibly_damaging | 0 | deleterious |
| rs769767842 | P176L | 0.015 | benign | 0.01 | deleterious |
| rs770625275 | D481E | NA | NA | NA | NA |
| rs770984783 | S225L | 0.459 | possibly_damaging | 0 | deleterious |
| rs771094362 | L153P | 0.999 | probably_damaging | 0 | deleterious |
| rs771094362 | L153R | 0.999 | probably_damaging | 0 | deleterious |
| rs771245131 | S161P | 0.001 | benign | 0.07 | tolerated |
| rs771245131 | S161A | 0.001 | benign | 0.07 | tolerated |
| rs771433825 | S419F | 0.947 | probably_damaging | 0.01 | deleterious |
| rs771748415 | P142A | 0.752 | possibly_damaging | 0.01 | deleterious |
| rs771836537 | L234S | 0.173 | benign | 0 | deleterious |
| rs771836537 | L234W | 0.173 | benign | 0 | deleterious |
| rs772238634 | C575S | 0 | benign | 0.18 | tolerated |
| rs772239179 | S123W | 0.794 | possibly_damaging | 0 | deleterious |
| rs772239179 | S123L | 0.794 | possibly_damaging | 0 | deleterious |
| rs772448788 | G94S | 0.001 | benign | 0.57 | tolerated |
| rs772593766 | A421V | 0.83 | possibly_damaging | 0 | deleterious |
| rs772706227 | V298M | 0.163 | benign | 0.03 | deleterious |
| rs773504394 | N505S | 0.998 | probably_damaging | 0 | deleterious |
| rs773504394 | N505I | 0.998 | probably_damaging | 0 | deleterious |
| rs773843241 | V482M | 0.311 | benign | 0.02 | deleterious |
| rs773843241 | V482L | 0.311 | benign | 0.02 | deleterious |
| rs773843241 | V482L | 0.311 | benign | 0.02 | deleterious |
| rs773906022 | G311S | 0.087 | benign | 0.17 | tolerated |
| rs774261081 | W512S | 0.999 | probably_damaging | 0 | deleterious |
| rs774312092 | W90S | 0 | benign | 0.14 | tolerated |
| rs774647122 | I368N | 0.992 | probably_damaging | 0 | deleterious |
| rs774946874 | N410K | NA | NA | NA | NA |
| rs775043563 | T420P | 0.129 | benign | 0.04 | deleterious |
| rs775043563 | T420A | 0.129 | benign | 0.04 | deleterious |
| rs776101618 | C96Y | 0.743 | possibly_damaging | 0.08 | tolerated |
| rs776101618 | C96S | 0.743 | possibly_damaging | 0.08 | tolerated |
| rs776953195 | A244G | 0.64 | possibly_damaging | 0 | deleterious |
| rs776953195 | A244V | 0.64 | possibly_damaging | 0 | deleterious |
| rs777060019 | I382T | 0.935 | probably_damaging | 0 | deleterious |
| rs777060019 | I382S | 0.935 | probably_damaging | 0 | deleterious |
| rs777145627 | A579E | 0.466 | possibly_damaging | 0.01 | deleterious_low_confidence |
| rs777444688 | V478M | 0.998 | probably_damaging | 0.01 | deleterious |
| rs777444688 | V478L | 0.998 | probably_damaging | 0.01 | deleterious |
| rs777546990 | S229F | 0.972 | probably_damaging | 0 | deleterious |
| rs777892391 | A436D | 0.936 | probably_damaging | 0 | deleterious |
| rs777892391 | A436G | 0.936 | probably_damaging | 0 | deleterious |
| rs777892391 | A436V | 0.936 | probably_damaging | 0 | deleterious |
| rs777903263 | T566M | 0.272 | benign | 0 | deleterious |
| rs779148944 | P472L | 0.999 | probably_damaging | 0 | deleterious |
| rs779265931 | R120Q | 0.012 | benign | 0.12 | tolerated |
| rs779418622 | V502I | 0.139 | benign | 0 | deleterious_low_confidence |
| rs779418622 | V502L | 0.139 | benign | 0 | deleterious_low_confidence |
| rs779637743 | V354I | 0.812 | possibly_damaging | 0 | deleterious |
| rs779637743 | V354F | 0.812 | possibly_damaging | 0 | deleterious |
| rs780102678 | F400L | 0.903 | possibly_damaging | 0 | deleterious |
| rs780115582 | C40W | 0 | benign | 0.19 | tolerated |
| rs780200003 | I131V | 0.401 | benign | 0.01 | deleterious |
| rs780200003 | I131F | 0.401 | benign | 0.01 | deleterious |
| rs780580396 | A359V | 1 | probably_damaging | 0 | deleterious |
| rs780970528 | I111M | 0.588 | possibly_damaging | 0 | deleterious |
| rs781425640 | Y156H | 0.999 | probably_damaging | 0 | deleterious |
| rs781425640 | Y156D | 0.999 | probably_damaging | 0 | deleterious |
| rs866153120 | A75D | 0.027 | benign | 0.05 | deleterious |
| rs866153120 | A75V | 0.027 | benign | 0.05 | deleterious |
| rs866159295 | S118R | 0.057 | benign | 0.01 | deleterious |
| rs866876745 | R71H | 0.995 | probably_damaging | 0 | deleterious |
| rs866876745 | R71L | 0.995 | probably_damaging | 0 | deleterious |
| rs867839970 | Q129P | 0.007 | benign | 0 | deleterious |
| rs867839970 | Q129R | 0.007 | benign | 0 | deleterious |
| rs868737708 | P179S | 0.463 | possibly_damaging | 0.04 | deleterious |
| rs869033002 | R119G | 0.966 | probably_damaging | 0 | deleterious |
| rs869033002 | R119W | 0.966 | probably_damaging | 0 | deleterious |
| rs869165879 | L519M | 0.061 | benign | 0.16 | tolerated |
| rs869218919 | D481N | 0.877 | possibly_damaging | 0.07 | tolerated |
| rs890075608 | G376S | 0.599 | possibly_damaging | 0 | deleterious_low_confidence |
| rs890629217 | Q356P | 0.973 | probably_damaging | 0 | deleterious |
| rs890629217 | Q356R | 0.973 | probably_damaging | 0 | deleterious |
| rs890987834 | A103T | 0.263 | benign | 0 | deleterious |
| rs897100989 | L490F | 1 | probably_damaging | 0 | deleterious |
| rs897150702 | R80W | 0.728 | possibly_damaging | 0 | deleterious |
| rs911360656 | T420M | 0.53 | possibly_damaging | 0 | deleterious_low_confidence |
| rs914435888 | R152Q | 0.005 | benign | 0.09 | tolerated |
| rs914435888 | R152L | 0.005 | benign | 0.09 | tolerated |
| rs936186701 | R361S | 1 | probably_damaging | 0 | deleterious |
| rs938160611 | R543S | 0.639 | possibly_damaging | 0.22 | tolerated |
| rs938442681 | L292R | 0.455 | possibly_damaging | 0.19 | tolerated |
| rs959857439 | D406N | 0.936 | probably_damaging | 0 | deleterious |
| rs959857439 | D406H | 0.936 | probably_damaging | 0 | deleterious |
| rs962124411 | I274V | 0.167 | benign | 0.39 | tolerated |
| rs971528785 | F104L | NA | NA | NA | NA |
| rs979262553 | G159E | 1 | probably_damaging | 0 | deleterious |
| rs979262553 | G159A | 1 | probably_damaging | 0 | deleterious |
| rs979262553 | G159V | 1 | probably_damaging | 0 | deleterious |
| rs980886088 | P270A | 0.881 | possibly_damaging | 0.02 | deleterious |
| rs980886088 | P270S | 0.881 | possibly_damaging | 0.02 | deleterious |
| rs991809705 | A173T | 0.998 | probably_damaging | 0 | deleterious |
| rs991809705 | A173S | 0.998 | probably_damaging | 0 | deleterious |
| rs997542525 | L278P | 0.039 | benign | 0.01 | deleterious |
| rs1007114265 | E113K | 0.024 | benign | 0.07 | tolerated |
| rs1017955040 | S228F | 0.982 | probably_damaging | 0 | deleterious |
| rs1018195590 | E113A | 0.001 | benign | 0 | deleterious |
| rs1024670343 | K266N | 0.947 | probably_damaging | 0 | deleterious |
| rs1032715657 | V86M | 0.005 | benign | 0.26 | tolerated |
| rs1032715657 | V86L | 0.005 | benign | 0.26 | tolerated |
| rs1034789713 | N235K | 0.001 | benign | 0.02 | deleterious |
| rs1035071310 | G395D | 0.994 | probably_damaging | 0 | deleterious |
| rs1035071310 | G395A | 0.994 | probably_damaging | 0 | deleterious |
| rs1035071310 | G395V | 0.994 | probably_damaging | 0 | deleterious |
| rs1038345456 | A79E | 0.146 | benign | 0.01 | deleterious |
| rs1038345456 | A79V | 0.146 | benign | 0.01 | deleterious |
| rs1038480938 | A6E | 0 | unknown | 0.1 | tolerated |
| rs1038480938 | A6V | 0 | unknown | 0.1 | tolerated |
| rs1051163566 | Q126H | 0.003 | benign | 0.01 | deleterious |
| rs1163737040 | T282I | 0.955 | probably_damaging | 0.01 | deleterious |
| rs1166974640 | A116T | 0.007 | benign | 0.03 | deleterious |
| rs1169627314 | W530R | 0.999 | probably_damaging | 0 | deleterious |
| rs1169627314 | W530R | 0.999 | probably_damaging | 0 | deleterious |
| rs1173206266 | E462A | 0.881 | possibly_damaging | 0 | deleterious |
| rs1173206266 | E462G | 0.881 | possibly_damaging | 0 | deleterious |
| rs1174275419 | P64R | 0.998 | probably_damaging | 0 | deleterious |
| rs1174275419 | P64L | 0.998 | probably_damaging | 0 | deleterious |
| rs1174882196 | A488V | 0.193 | benign | 0.04 | deleterious |
| rs1179973655 | L522V | 0.106 | benign | 0.08 | tolerated |
| rs1180600586 | C564W | 0 | benign | 0.02 | deleterious |
| rs1188055566 | S499C | 0.998 | probably_damaging | 0 | deleterious |
| rs1188055566 | S499F | 0.998 | probably_damaging | 0 | deleterious |
| rs1191944886 | M175I | 0 | benign | 0.43 | tolerated |
| rs1191944886 | M175I | 0 | benign | 0.43 | tolerated |
| rs1192758278 | K547N | 0.219 | benign | 0.02 | deleterious_low_confidence |
| rs1192758278 | K547N | 0.219 | benign | 0.02 | deleterious_low_confidence |
| rs1195757670 | A291T | 0.953 | probably_damaging | 0 | deleterious |
| rs1195888869 | L369P | 1 | probably_damaging | 0 | deleterious |
| rs1201909102 | I111L | 0.012 | benign | 0.02 | deleterious |
| rs1201909102 | I111V | 0.012 | benign | 0.02 | deleterious |
| rs1202852211 | T236A | 0 | benign | 0.14 | tolerated |
| rs1215757455 | Q72K | 0.017 | benign | 0.19 | tolerated |
| rs1218418788 | A537D | 0.932 | probably_damaging | 0 | deleterious_low_confidence |
| rs1221389630 | E468Q | 0.998 | probably_damaging | 0 | deleterious |
| rs1222822124 | V256I | 0.003 | benign | 0.01 | deleterious |
| rs1222822124 | V256F | 0.003 | benign | 0.01 | deleterious |
| rs1224927529 | F315L | 0.99 | probably_damaging | 0.02 | deleterious |
| rs1227931926 | I162T | 0.634 | possibly_damaging | 0 | deleterious |
| rs1228820479 | H352Y | 1 | probably_damaging | 0 | deleterious |
| rs1237810987 | I358V | 0.028 | benign | 0.61 | tolerated |
| rs1239850116 | G448E | 1 | probably_damaging | 0 | deleterious |
| rs1240259518 | A211V | 0.041 | benign | 0.19 | tolerated |
| rs1240286523 | G455S | 0.069 | benign | 0.17 | tolerated |
| rs1240286523 | G455C | 0.069 | benign | 0.17 | tolerated |
| rs1240948632 | L287R | 0.933 | probably_damaging | 0 | deleterious |
| rs1244021484 | M341T | 0.129 | benign | 0.01 | deleterious |
| rs1245195510 | R20H | 0 | unknown | 0.13 | tolerated |
| rs1245195510 | R20L | 0 | unknown | 0.13 | tolerated |
| rs1246576354 | P46A | 0.027 | benign | 0.1 | tolerated_low_confidence |
| rs1246576354 | P46S | 0.027 | benign | 0.1 | tolerated_low_confidence |
| rs1247061281 | E252D | NA | NA | 0.24 | tolerated_low_confidence |
| rs1250268293 | G60E | 0.084 | benign | 0.02 | deleterious |
| rs1255549417 | P56T | 0 | benign | 0.1 | tolerated |
| rs1255549417 | P56A | 0 | benign | 0.1 | tolerated |
| rs1256466032 | Q126E | 0 | benign | 0 | deleterious |
| rs1259028085 | T538S | 0.172 | benign | 0.01 | deleterious |
| rs1259028085 | T538I | 0.172 | benign | 0.01 | deleterious |
| rs1271227682 | E127G | 0.061 | benign | 0 | deleterious |
| rs1271975485 | L108V | 0.013 | benign | 0.31 | tolerated |
| rs1280351293 | I162V | 0.204 | benign | 0.03 | deleterious |
| rs1282435860 | L511I | 0.999 | probably_damaging | 0 | deleterious |
| rs1282435860 | L511F | 0.999 | probably_damaging | 0 | deleterious |
| rs1285137369 | N521K | 0.003 | benign | 0.23 | tolerated |
| rs1286502283 | R71S | 0.991 | probably_damaging | 0 | deleterious |
| rs1286950600 | G210R | 0 | benign | 0.21 | tolerated |
| rs1286950600 | G210R | 0 | benign | 0.21 | tolerated |
| rs1291407025 | V405A | 0.97 | probably_damaging | 0.01 | deleterious |
| rs1300454219 | L102M | 0.717 | possibly_damaging | 0 | deleterious |
| rs1300454219 | L102V | 0.717 | possibly_damaging | 0 | deleterious |
| rs1302762072 | V350G | 0.999 | probably_damaging | 0 | deleterious |
| rs1303202391 | R263S | 0.003 | benign | 0.4 | tolerated |
| rs1303935100 | E231G | 0.047 | benign | 0 | deleterious |
| rs1309593098 | C387F | 0.985 | probably_damaging | 0 | deleterious |
| rs1309867551 | C125Y | 0.919 | probably_damaging | 0 | deleterious |
| rs1309867551 | C125S | 0.919 | probably_damaging | 0 | deleterious |
| rs1309867551 | C125F | 0.919 | probably_damaging | 0 | deleterious |
| rs1313244602 | V277I | 0.809 | possibly_damaging | 0.05 | tolerated |
| rs1313969452 | G105R | 0.105 | benign | 0.01 | deleterious |
| rs1313969452 | G105W | 0.105 | benign | 0.01 | deleterious |
| rs1314235592 | N367K | 1 | probably_damaging | 0 | deleterious |
| rs1318236292 | L539F | 0.914 | probably_damaging | 0.02 | deleterious |
| rs1318357743 | R45H | 0.285 | benign | 0 | deleterious |
| rs1318357743 | R45P | 0.285 | benign | 0 | deleterious |
| rs1320834693 | E551Q | 0.998 | probably_damaging | 0.01 | deleterious |
| rs1328305235 | S187N | 0.082 | benign | 0.12 | tolerated |
| rs1331775010 | F69L | 0.379 | benign | 0 | deleterious |
| rs1333621796 | E117K | 0.767 | possibly_damaging | 0.01 | deleterious |
| rs1334106557 | C166R | 0.946 | probably_damaging | 0 | deleterious |
| rs1339849774 | G23D | 0.019 | benign | 0.11 | tolerated_low_confidence |
| rs1339849774 | G23V | 0.019 | benign | 0.11 | tolerated_low_confidence |
| rs1342739662 | E565A | 0.998 | probably_damaging | 0 | deleterious |
| rs1342739662 | E565G | 0.998 | probably_damaging | 0 | deleterious |
| rs1344177821 | G159R | 1 | probably_damaging | 0 | deleterious |
| rs1344177821 | G159R | 1 | probably_damaging | 0 | deleterious |
| rs1344260414 | A93T | 0.267 | benign | 0.01 | deleterious |
| rs1344260414 | A93S | 0.267 | benign | 0.01 | deleterious |
| rs1345721785 | P56L | 0.012 | benign | 0.04 | deleterious |
| rs1347237673 | L7M | 0 | unknown | 0 | deleterious |
| rs1349949487 | A89T | 0.003 | benign | 0.18 | tolerated |
| rs1349949487 | A89P | 0.003 | benign | 0.18 | tolerated |
| rs1352475242 | A213T | 0.007 | benign | 0.12 | tolerated |
| rs1352475242 | A213P | 0.007 | benign | 0.12 | tolerated |
| rs1358387259 | F281S | 0.992 | probably_damaging | 0 | deleterious |
| rs1359360604 | G457D | 0.987 | probably_damaging | 0.02 | deleterious |
| rs1361880248 | E462K | 0.818 | possibly_damaging | 0 | deleterious |
| rs1361880248 | E462Q | 0.818 | possibly_damaging | 0 | deleterious |
| rs1366918258 | A130P | 0.767 | possibly_damaging | 0 | deleterious |
| rs1366918258 | A130S | 0.767 | possibly_damaging | 0 | deleterious |
| rs1375755897 | L344M | 1 | probably_damaging | 0 | deleterious |
| rs1377297630 | W577C | 0.924 | probably_damaging | 0 | deleterious |
| rs1377868920 | L509V | 0.975 | probably_damaging | 0 | deleterious |
| rs1383828848 | V428M | 0.678 | possibly_damaging | 0 | deleterious_low_confidence |
| rs1385309950 | R68P | 0.558 | possibly_damaging | 0 | deleterious |
| rs1385309950 | R68L | 0.558 | possibly_damaging | 0 | deleterious |
| rs1389741334 | A116E | 0.127 | benign | 0.02 | deleterious |
| rs1389741334 | A116G | 0.127 | benign | 0.02 | deleterious |
| rs1389741334 | A116V | 0.127 | benign | 0.02 | deleterious |
| rs1390363246 | W48C | 0.098 | benign | 0.02 | deleterious_low_confidence |
| rs1391821022 | Q12R | 0 | unknown | 0 | deleterious |
| rs1391821022 | Q12L | 0 | unknown | 0 | deleterious |
| rs1396603457 | P52T | 0.079 | benign | 0.01 | deleterious_low_confidence |
| rs1396603457 | P52S | 0.079 | benign | 0.01 | deleterious_low_confidence |
| rs1396751963 | C412S | 0.012 | benign | 0.37 | tolerated |
| rs1396751963 | C412R | 0.012 | benign | 0.37 | tolerated |
| rs1400562167 | D362N | 1 | probably_damaging | 0 | deleterious |
| rs1400562167 | D362Y | 1 | probably_damaging | 0 | deleterious |
| rs1403635755 | Q397H | NA | NA | NA | NA |
| rs1404815028 | L572F | 0.003 | benign | 0.61 | tolerated_low_confidence |
| rs1407018357 | Q456H | NA | NA | NA | NA |
| rs1409111496 | A78E | 0.622 | possibly_damaging | 0 | deleterious |
| rs1409111496 | A78V | 0.622 | possibly_damaging | 0 | deleterious |
| rs1410017139 | C96S | 0.361 | benign | 0.13 | tolerated |
| rs1410017139 | C96R | 0.361 | benign | 0.13 | tolerated |
| rs1412577584 | V331A | 0 | benign | 0.34 | tolerated |
| rs1421454894 | K114E | 0.005 | benign | 0.05 | deleterious |
| rs1426133466 | A459V | 0.011 | benign | 0.07 | tolerated |
| rs1426868527 | R98Q | 0.335 | benign | 0 | deleterious |
| rs1426868527 | R98L | 0.335 | benign | 0 | deleterious |
| rs1427965886 | G32R | 0.998 | probably_damaging | 0.11 | tolerated |
| rs1427965886 | G32W | 0.998 | probably_damaging | 0.11 | tolerated |
| rs1427977954 | A200T | 0.027 | benign | 0.15 | tolerated |
| rs1428369222 | K319N | 0.947 | probably_damaging | 0 | deleterious |
| rs1428369222 | K319N | 0.947 | probably_damaging | 0 | deleterious |
| rs1429153319 | Q239H | 0.551 | possibly_damaging | 0.04 | deleterious |
| rs1430354103 | R58G | 0 | benign | 0.04 | deleterious |
| rs1430699620 | A415V | 0.997 | probably_damaging | 0 | deleterious |
| rs1433102917 | Y258C | 0.356 | benign | 0.05 | deleterious |
| rs1443552897 | G43R | 0.001 | benign | 0 | deleterious_low_confidence |
| rs1443552897 | G43R | 0.001 | benign | 0 | deleterious_low_confidence |
| rs1449858096 | I446V | 0.619 | possibly_damaging | 0 | deleterious |
| rs1449858096 | I446F | 0.619 | possibly_damaging | 0 | deleterious |
| rs1460162636 | P423T | 0.619 | possibly_damaging | 0 | deleterious |
| rs1464230596 | R464C | 0.995 | probably_damaging | 0.05 | tolerated |
| rs1464914100 | Y253F | 0.635 | possibly_damaging | 0.27 | tolerated |
| rs1465298222 | A579S | 0.066 | benign | 0.47 | tolerated_low_confidence |
| rs1465983168 | R119Q | 0.073 | benign | 0 | deleterious |
| rs1469164217 | G60R | 0.005 | benign | 0.06 | tolerated |
| rs1469164217 | G60R | 0.005 | benign | 0.06 | tolerated |
| rs1478952289 | E551A | 0.998 | probably_damaging | 0 | deleterious |
| rs1478952289 | E551G | 0.998 | probably_damaging | 0 | deleterious |
| rs1480364441 | D373N | 0.998 | probably_damaging | 0.01 | deleterious |
| rs1482407232 | P95T | 0.857 | possibly_damaging | 0 | deleterious |
| rs1482407232 | P95S | 0.857 | possibly_damaging | 0 | deleterious |
| rs1488635869 | A93E | 0.267 | benign | 0.01 | deleterious |
| rs1488635869 | A93V | 0.267 | benign | 0.01 | deleterious |
| rs1553145550 | C92F | 0 | benign | 0 | deleterious |
| rs1553145560 | I111S | 0.793 | possibly_damaging | 0 | deleterious |
| rs1553145929 | V170G | 0.998 | probably_damaging | 0 | deleterious |
| rs1553145944 | G202R | 0.251 | benign | 0.08 | tolerated |
| rs1553146419 | R263G | 0 | benign | 0.01 | deleterious_low_confidence |
| rs1553146430 | L278V | 0.013 | benign | 0.02 | deleterious_low_confidence |
| rs1553146562 | M342I | 0.968 | probably_damaging | 0.01 | deleterious |
| rs1553146578 | H360R | 1 | probably_damaging | 0 | deleterious |
| rs1553146818 | G409A | 0.996 | probably_damaging | 0 | deleterious |
| rs1553146818 | G409V | 0.996 | probably_damaging | 0 | deleterious |
| rs1553146821 | P416R | 1 | probably_damaging | 0 | deleterious |
| rs1553146821 | P416L | 1 | probably_damaging | 0 | deleterious |
| rs1553146822 | E417G | 1 | probably_damaging | 0 | deleterious |
| rs1553146849 | S419P | 0.164 | benign | 0.03 | deleterious |
| rs1553146861 | A427E | 0.438 | benign | 0.01 | deleterious |
| rs1553146877 | I442T | 0.758 | possibly_damaging | 0 | deleterious |
| rs1553146903 | L489P | 1 | probably_damaging | 0 | deleterious |
| rs1553147010 | P498L | 0.632 | possibly_damaging | 0.35 | tolerated |
| rs1553147049 | L574F | 0.979 | probably_damaging | 0.03 | deleterious |
| rs1553147052 | C575R | 0 | benign | 0.24 | tolerated |
| rs1557561324 | G197D | 0.024 | benign | 0.11 | tolerated |
| rs1557561324 | G197V | 0.024 | benign | 0.11 | tolerated |
| rs1557562082 | N235I | 0.098 | benign | 0 | deleterious |
| rs1557564850 | L338F | 0.975 | probably_damaging | 0.13 | tolerated |
| rs1557564978 | P374S | 0.005 | benign | 0.89 | tolerated |
| rs1557566897 | W437R | 1 | probably_damaging | 0 | deleterious |
| rs1570396856 | L17Q | 0 | unknown | 0 | deleterious |
| rs1570396856 | L17P | 0 | unknown | 0 | deleterious |
| rs1570396862 | L18Q | 0 | unknown | 0 | deleterious_low_confidence |
| rs1570396870 | L19Q | 0 | unknown | 0.19 | tolerated |
| rs1570396903 | G30D | 0.132 | benign | 0 | deleterious_low_confidence |
| rs1570396903 | G30V | 0.132 | benign | 0 | deleterious_low_confidence |
| rs1570397179 | L81F | 0.556 | possibly_damaging | 0 | deleterious |
| rs1570397205 | W90R | 0 | benign | 0.13 | tolerated |
| rs1570403956 | P300L | 0.987 | probably_damaging | 0 | deleterious |
| rs1570404869 | T324P | 0.998 | probably_damaging | 0 | deleterious |
| rs1570404990 | V354G | 0.834 | possibly_damaging | 0.01 | deleterious |
| rs1570407166 | V381G | 0.959 | probably_damaging | 0 | deleterious |
| rs1570407771 | A436T | 0.219 | benign | 0.04 | deleterious |
| rs1570407771 | A436S | 0.219 | benign | 0.04 | deleterious |
| rs1570407855 | L449I | 0.987 | probably_damaging | 0.07 | tolerated |
| rs1570407855 | L449F | 0.987 | probably_damaging | 0.07 | tolerated |
| rs1570407857 | V450D | 0 | benign | 0.72 | tolerated |
| rs1570407857 | V450A | 0 | benign | 0.72 | tolerated |
| rs1570407909 | K458R | 0.001 | benign | 0.62 | tolerated |
| rs1570407915 | K458N | 0.06 | benign | 0.29 | tolerated |
| rs1570407922 | H460P | 0.376 | benign | 0.14 | tolerated |
| rs1570407929 | L461F | 0.462 | possibly_damaging | 0 | deleterious |
| rs1570407952 | S463N | 0.911 | probably_damaging | 0.03 | deleterious |
| rs1570407952 | S463T | 0.911 | probably_damaging | 0.03 | deleterious |
| rs1570409132 | R497S | 0.999 | probably_damaging | 0 | deleterious |
| rs1570409245 | I516L | 0 | benign | 0.39 | tolerated |
| rs1570409245 | I516V | 0 | benign | 0.39 | tolerated |
| rs1570409339 | V528I | 0 | benign | 0.68 | tolerated_low_confidence |
| rs1570409349 | G529A | 0.001 | benign | 0.56 | tolerated |
| rs1570409457 | A560S | 0.734 | possibly_damaging | 0.14 | tolerated_low_confidence |
| rs1570409472 | T566A | 0 | benign | 0.45 | tolerated |
| rs2053012917 | M1I | 0 | unknown | 0 | deleterious |
| rs2053013079 | G8S | 0 | unknown | 0.4 | tolerated |
| rs2053013114 | G10S | 0 | unknown | 0 | deleterious |
| rs2053013114 | G10R | 0 | unknown | 0 | deleterious |
| rs2053013176 | G10A | 0 | unknown | 0 | deleterious |
| rs2053013176 | G10V | 0 | unknown | 0 | deleterious |
| rs2053013259 | L11Q | 0 | unknown | 0 | deleterious |
| rs2053013403 | L13M | 0 | unknown | 0 | deleterious |
| rs2053013433 | G14S | 0 | unknown | 0 | deleterious_low_confidence |
| rs2053015060 | P25H | 0.996 | probably_damaging | 0 | deleterious |
| rs2053015060 | P25L | 0.996 | probably_damaging | 0 | deleterious |
| rs2053015185 | R27W | 0.325 | benign | 0.01 | deleterious_low_confidence |
| rs2053015233 | A28T | 0 | benign | 0.12 | tolerated |
| rs2053015285 | A28V | 0 | benign | 0.19 | tolerated |
| rs2053015599 | G32E | 0.998 | probably_damaging | 0.06 | tolerated |
| rs2053015697 | G35S | 0.769 | possibly_damaging | 0.02 | deleterious_low_confidence |
| rs2053015697 | G35C | 0.769 | possibly_damaging | 0.02 | deleterious_low_confidence |
| rs2053015724 | G35D | 0.846 | possibly_damaging | 0.01 | deleterious |
| rs2053015724 | G35A | 0.846 | possibly_damaging | 0.01 | deleterious |
| rs2053015747 | A37V | 0.053 | benign | 0.55 | tolerated |
| rs2053017045 | G51R | 0.435 | benign | 0.02 | deleterious |
| rs2053017529 | R57H | 0.197 | benign | 0.03 | deleterious |
| rs2053018518 | L63H | 0.998 | probably_damaging | 0 | deleterious |
| rs2053020457 | G97S | 0.541 | possibly_damaging | 0.1 | tolerated |
| rs2053020457 | G97C | 0.541 | possibly_damaging | 0.1 | tolerated |
| rs2053020653 | A99P | 0.699 | possibly_damaging | 0 | deleterious |
| rs2053020653 | A99S | 0.699 | possibly_damaging | 0 | deleterious |
| rs2053020842 | A103D | 0.489 | possibly_damaging | 0 | deleterious |
| rs2053020842 | A103V | 0.489 | possibly_damaging | 0 | deleterious |
| rs2053023341 | S118N | 0.038 | benign | 0.05 | tolerated |
| rs2053023467 | R120G | 0.728 | possibly_damaging | 0.01 | deleterious |
| rs2053023467 | R120W | 0.728 | possibly_damaging | 0.01 | deleterious |
| rs2053072441 | A130G | 0.331 | benign | 0 | deleterious |
| rs2053072441 | A130V | 0.331 | benign | 0 | deleterious |
| rs2053073292 | F151L | 0.727 | possibly_damaging | 0 | deleterious |
| rs2053073741 | Q160K | 0.009 | benign | 0.26 | tolerated |
| rs2053074109 | A168V | 0.998 | probably_damaging | 0 | deleterious |
| rs2053074232 | V170M | 0.999 | probably_damaging | 0 | deleterious |
| rs2053074382 | A173V | 0.998 | probably_damaging | 0 | deleterious |
| rs2053075003 | K186R | 0.005 | benign | 0.28 | tolerated |
| rs2053075565 | S199R | 0.049 | benign | 0.14 | tolerated |
| rs2053075565 | S199G | 0.049 | benign | 0.14 | tolerated |
| rs2053075840 | G204A | 0 | benign | 0.55 | tolerated |
| rs2053076441 | P212L | 0.09 | benign | 0.04 | deleterious |
| rs2053098150 | S228T | 0.919 | probably_damaging | 0 | deleterious |
| rs2053098606 | S229P | 0.085 | benign | 0 | deleterious |
| rs2053151529 | G264D | 0.052 | benign | 0.04 | deleterious |
| rs2053151529 | G264V | 0.052 | benign | 0.04 | deleterious |
| rs2053151636 | K266R | 0.255 | benign | 0.09 | tolerated |
| rs2053153305 | F281L | 0.304 | benign | 0 | deleterious |
| rs2053169573 | Q327R | 0.489 | possibly_damaging | 0.01 | deleterious |
| rs2053169621 | N332S | 0.006 | benign | 0.84 | tolerated |
| rs2053169742 | P334R | 1 | probably_damaging | 0 | deleterious |
| rs2053170545 | L344Q | 1 | probably_damaging | 0 | deleterious |
| rs2053170545 | L344P | 1 | probably_damaging | 0 | deleterious |
| rs2053170545 | L344R | 1 | probably_damaging | 0 | deleterious |
| rs2053170598 | L346P | 1 | probably_damaging | 0 | deleterious |
| rs2053171048 | G357C | 0.999 | probably_damaging | 0 | deleterious |
| rs2053171075 | G357D | 0.997 | probably_damaging | 0 | deleterious |
| rs2053171075 | G357V | 0.997 | probably_damaging | 0 | deleterious |
| rs2053171704 | E371A | 0.997 | probably_damaging | 0 | deleterious |
| rs2053171727 | L372V | 0.456 | possibly_damaging | 0 | deleterious |
| rs2053171796 | D373E | 0.996 | probably_damaging | 0.01 | deleterious |
| rs2053216591 | E392K | 0.255 | benign | 0.05 | tolerated |
| rs2053217073 | Q397P | 0.023 | benign | 0.49 | tolerated |
| rs2053217073 | Q397R | 0.023 | benign | 0.49 | tolerated |
| rs2053217273 | S401N | 0.003 | benign | 0.42 | tolerated |
| rs2053217339 | W403R | 0.378 | benign | 0.04 | deleterious |
| rs2053228553 | V418A | 0.995 | probably_damaging | 0 | deleterious |
| rs2053228553 | V418G | 0.995 | probably_damaging | 0 | deleterious |
| rs2053229322 | I429T | 0.913 | probably_damaging | 0 | deleterious |
| rs2053229491 | S432G | 0.457 | possibly_damaging | 0.02 | deleterious |
| rs2053229557 | K433E | 0.997 | probably_damaging | 0 | deleterious |
| rs2053232597 | R483K | 0.276 | benign | 0.59 | tolerated |
| rs2053232768 | R487T | 0.935 | probably_damaging | 0.03 | deleterious |
| rs2053233024 | Q491E | 0.771 | possibly_damaging | 0.01 | deleterious |
| rs2053233063 | Q491H | 0.417 | benign | 0.05 | tolerated |
| rs2053252151 | N505H | 0.997 | probably_damaging | 0.01 | deleterious |
| rs2053252151 | N505D | 0.997 | probably_damaging | 0.01 | deleterious |
| rs2053252318 | L507I | 0.997 | probably_damaging | 0 | deleterious |
| rs2053252318 | L507F | 0.997 | probably_damaging | 0 | deleterious |
| rs2053252478 | S510C | 0.96 | probably_damaging | 0.03 | deleterious |
| rs2053252525 | S510I | 0.898 | possibly_damaging | 0 | deleterious |
| rs2053252695 | G513D | 1 | probably_damaging | 0 | deleterious |
| rs2053252695 | G513V | 1 | probably_damaging | 0 | deleterious |
| rs2053252936 | I516T | 0.001 | benign | 0.25 | tolerated |
| rs2053252936 | I516S | 0.001 | benign | 0.25 | tolerated |
| rs2053253036 | A518V | 0.058 | benign | 0 | deleterious |
| rs2053254496 | T538P | 0.481 | possibly_damaging | 0.01 | deleterious |
| rs2053254771 | L539S | 0.936 | probably_damaging | 0 | deleterious_low_confidence |
| rs2053255579 | C549Y | 0.039 | benign | 0.08 | tolerated_low_confidence |
| rs2053255579 | C549F | 0.039 | benign | 0.08 | tolerated_low_confidence |
| rs2053255687 | V550M | 0.265 | benign | 0 | deleterious_low_confidence |
| rs2053255727 | V550A | 0.568 | possibly_damaging | 0.02 | deleterious |
| rs2053255892 | T552R | 0.921 | probably_damaging | 0 | deleterious |
| rs2053255892 | T552I | 0.921 | probably_damaging | 0 | deleterious |
| rs2053255975 | M556I | 0.036 | benign | 0.02 | deleterious |
| rs2053256265 | L562M | NA | NA | NA | NA |
| rs2053257033 | C568Y | 0 | benign | 1 | tolerated |
| rs2053257292 | L573F | 0.088 | benign | 0.12 | tolerated_low_confidence |
| rs2053258096 | L581V | 0.066 | benign | 0.05 | deleterious_low_confidence |
| rs2154533495 | Q72R | 0.024 | benign | 0.26 | tolerated |
| rs2154533498 | C92W | NA | NA | NA | NA |
| rs2154533505 | A121V | 0.12 | benign | 0 | deleterious |
| rs2154533641 | L216F | 0.03 | benign | 0.02 | deleterious |
| rs2154533641 | L216F | 0.03 | benign | 0.02 | deleterious |
| rs2154533700 | E240V | 0.975 | probably_damaging | 0 | deleterious |
| rs2154533870 | T324I | 0.998 | probably_damaging | 0 | deleterious |
| rs2154534008 | F453L | 0.999 | probably_damaging | 0 | deleterious |
| rs2154534075 | L539M | 0.457 | possibly_damaging | 0 | deleterious |
| rs2154534076 | R543G | 0.026 | benign | 0.38 | tolerated |

**Supplementary Table 3.** Identification of intrinsically disordered regions (IDRs) and binding-prone segments in PINK1. Residue-wise disorder propensity was evaluated using IUPred and ANCHOR, with MetaPredict used for regional classification. Consensus interpretation highlights two disordered regions (IDR-1: 1–136 and IDR-2: 175–211) and a linker/transition segment enriched in disorder-to-order binding potential. Residues with elevated ANCHOR scores indicate potential molecular recognition features (MoRFs) involved in protein–protein interactions.

| **Pink1**  **Position** | **Residue** | **IUPred Score** | **ANCHOR Score** | **MetaPredict Region** | **Consensus**  **Interpretation** |
| --- | --- | --- | --- | --- | --- |
| 42 | R | 0.5419 | 0.32 | IDR-1 (1–136) | Disordered |
| 43 | G | 0.5419 | 0.34 | IDR-1 | Disordered |
| 44 | E | 0.5055 | 0.36 | IDR-1 | Disordered |
| 46 | P | 0.5176 | 0.38 | IDR-1 | Disordered |
| 48 | W | 0.5139 | 0.41 | IDR-1 | Disordered |
| 52 | P | 0.5665 | 0.44 | IDR-1 | Disordered |
| 54 | A | 0.5055 | 0.42 | IDR-1 | Disordered |
| 55 | E | 0.5098 | 0.45 | IDR-1 | Disordered |
| 56 | P | 0.5055 | 0.47 | IDR-1 | Disordered |
| 135 | K | 0.5176 | 0.48 | IDR-1 (boundary) | Disordered |
| 136 | S | 0.6375 | 0.52 | IDR-1 (boundary) | Disordered + Binding-prone |
| 137 | K | 0.6227 | 0.54 | Linker/transition | Disordered + Binding-prone |
| 138 | P | 0.5098 | 0.55 | Linker/transition | Disordered + Binding-prone |
| 139 | G | 0.6227 | 0.57 | Linker/transition | Disordered + Binding-prone |
| 140 | P | 0.6136 | 0.58 | Linker/transition | Disordered + Binding-prone |
| 141 | D | 0.5211 | 0.59 | Linker/transition | Disordered + Binding-prone |
| 142 | P | 0.6227 | 0.61 | Linker/transition | Disordered + Binding-prone |
| 143 | L | 0.6427 | 0.63 | Linker/transition | Disordered + Binding-prone |
| 144 | D | 0.6482 | 0.65 | Linker/transition | Disordered + Binding-prone |
| 145 | T | 0.6531 | 0.67 | Linker/transition | Disordered + Binding-prone |
| 146 | R | 0.5296 | 0.68 | Linker/transition | Disordered + Binding-prone |
| 182 | L | 0.5211 | 0.60 | IDR-2 (175–211) | Disordered + Binding-prone |
| 183 | E | 0.5055 | 0.61 | IDR-2 | Disordered + Binding-prone |
| 184 | V | 0.5456 | 0.63 | IDR-2 | Disordered + Binding-prone |
| 185 | T | 0.5342 | 0.65 | IDR-2 | Disordered + Binding-prone |
| 186 | K | 0.6136 | 0.68 | IDR-2 | Disordered + Binding-prone |
| 187 | S | 0.5707 | 0.70 | IDR-2 | Disordered + Binding-prone |
| 188 | T | 0.5758 | 0.71 | IDR-2 | Disordered + Binding-prone |
| 189 | G | 0.6755 | 0.74 | IDR-2 | Disordered + Binding-prone |
| 190 | L | 0.5951 | 0.76 | IDR-2 | Disordered + Binding-prone |
| 191 | L | 0.6269 | 0.78 | IDR-2 | Disordered + Binding-prone |
| 192 | P | 0.6136 | 0.80 | IDR-2 | Disordered + Binding-prone |
| 193 | G | 0.7369 | 0.82 | IDR-2 | Disordered + Binding-prone |
| 194 | R | 0.7209 | 0.84 | IDR-2 | Disordered + Binding-prone |
| 195 | G | 0.8085 | 0.86 | IDR-2 | Disordered + Binding-prone |
| 196 | P | 0.8198 | 0.88 | IDR-2 | Disordered + Binding-prone |
| 197 | G | 0.8279 | 0.89 | IDR-2 | Disordered + Binding-prone |
| 198 | T | 0.8013 | 0.90 | IDR-2 | Disordered + Binding-prone |
| 199 | S | 0.8235 | 0.91 | IDR-2 | Disordered + Binding-prone |
| 200 | A | 0.8279 | 0.92 | IDR-2 | Disordered + Binding-prone |
| 201 | P | 0.8713 | 0.93 | IDR-2 | Disordered + Binding-prone |
| 202 | G | 0.9381 | 0.95 | IDR-2 | Highest disorder + strongest MoRF |
| 203 | E | 0.9057 | 0.93 | IDR-2 | Disordered + Binding-prone |
| 204 | G | 0.8279 | 0.91 | IDR-2 | Disordered + Binding-prone |
| 205 | Q | 0.8565 | 0.89 | IDR-2 | Disordered + Binding-prone |
| 206 | E | 0.7799 | 0.86 | IDR-2 | Disordered + Binding-prone |
| 207 | R | 0.7120 | 0.83 | IDR-2 | Disordered + Binding-prone |
| 208 | A | 0.5951 | 0.80 | IDR-2 | Disordered + Binding-prone |
| 209 | P | 0.5854 | 0.78 | IDR-2 | Disordered + Binding-prone |
| 210 | G | 0.5456 | 0.75 | IDR-2 | Disordered + Binding-prone |
| 211 | A | 0.5456 | 0.73 | IDR-2 | Disordered + Binding-prone |
| 261 | S | 0.5055 | 0.41 | Ordered  (212–581) | Local disorder |
| 262 | K | 0.5419 | 0.44 | Ordered | Local disorder |
| 263 | R | 0.5382 | 0.46 | Ordered | Local disorder |
| 264 | G | 0.5419 | 0.48 | Ordered | Local disorder |
| 416 | P | 0.5254 | 0.39 | Ordered | Isolated disorder |

**Supplementary Table 4.** Domain architecture and functional annotation of human PINK1. Pfam-based mapping identified key structural regions, including the mitochondrial targeting sequence, regulatory segments, and the protein kinase domain with conserved catalytic motifs. Integration of IUPred-based Pfam domain-specific mapping linked intrinsically disordered regions to regulatory and linker segments, highlighting their potential role in protein interactions and functional flexibility.

| **Region** | **PFAM**  **Domain Analysis** | **Motif / Structural Feature** | **Conserved Residues (Human PINK1 Position)** | **Functional Role** |
| --- | --- | --- | --- | --- |
| N-terminal targeting region | Mitochondrial targeting sequence | Amphipathic helix | R10, L13, L16, L19, F20, G24, R27, Y28 | Mitochondrial import and membrane targeting |
| Transmembrane proximal region | Mitochondrial localization region | Hydrophobic segment | L45, F49, V52, L56 | Membrane association |
| Pre-kinase regulatory region | Regulatory linker | Conserved loop | R90, L95, F98 | Structural stabilization before kinase domain |
| Kinase N-lobe | Protein kinase domain | Glycine-rich ATP binding loop (GxGxxG motif) | G309, K310, G312, **T313**, I314, G315, K316 | ATP phosphate coordination |
| Kinase N-lobe | Protein kinase domain | VAIK catalytic motif | V327, A328, I329, K330 | ATP positioning |
| Kinase hinge region | Protein kinase domain | Structural hinge preceding catalytic loop | V340, D341, Y343, P344, V345, **L347**, H348, P349 | Stabilizes catalytic pocket |
| Catalytic loop | Protein kinase domain | HRDLKPEN motif | H357, R358, D359, L360, K361, P362, N363 | Catalytic phospho-Transfer |
| Activation loop | Protein kinase domain | DFG motif | D384, F385, G386 | Mg²⁺ coordination and kinase activation |
| Activation segment | Protein kinase domain | Substrate positioning loop | T386, V393, A394, H395, R396, D397 | Substrate orientation |
| Substrate recognition region | Protein kinase domain | Ub-binding surface | E417, G418, H419, I420, L421, K423 | Ubiquitin recognition |
| C-terminal kinase lobe | Protein kinase domain | Structural helices | V450, L455, E460, D462 | Kinase fold stabilization |
| Post-kinase regulatory region | C-terminal regulatory domain | Conserved structural motif | D525, G526, C527, P528, W529 | Structural integrity |
| Extreme C-terminal tail | Regulatory region | Terminal helix | R560, V562, A564, L566, H568, L570, S571 | Protein stability and regulation |

**Supplementary Table 5.** Functional mapping of PINK1 PTM sites showing phosphorylation and ubiquitination residues across different domains, with emphasis on kinase domain residues critical for catalytic activity and substrate interaction.

| **Residue** | **PhosphositePlus**  **PTM Type** | **NetPhos Prediction** | **NetPhos**  **Domain / Region** | **UniProt** | |
| --- | --- | --- | --- | --- | --- |
|  |  |  |  | **Residues** | **Regions** |
| S137 | Phosphorylation | Moderate | Near TM region | Ser228 | Autophosphorylation site important for kinase activation |
| S144 | Phosphorylation | High | N-terminal regulatory region | Ser402 | Required for catalytic activity and substrate phosphorylation |
| T175 | Phosphorylation | Moderate | Beginning of kinase domain | Lys137 | Ubiquitination |
| S228 | Phosphorylation | High | Kinase domain | ~156–511 | Catalytic region responsible for phosphorylation of ubiquitin and Parkin |
| S229 | Phosphorylation | Moderate | Kinase domain | 1–34 | Directs protein import into mitochondria |
| T257 | Phosphorylation | Moderate | Kinase domain | - | - |
| S265 | Phosphorylation | High | Kinase domain | - | - |
| T313 | Phosphorylation | High | Kinase domain | - | - |
| S402 | Phosphorylation | High | Kinase domain | - | - |
| S407 | Phosphorylation | Moderate | Kinase domain | - | - |
| S465 | Phosphorylation | Moderate | Kinase domain | - | - |
| S495 | Phosphorylation | High | Kinase domain | - | - |
| S548 | Phosphorylation | Moderate | C-terminal tail | - | - |

**Supplementary Table 6.** Average Molecular Dynamics Metrics Across Three Independent Replicates for Apo-PINK1 and PINK1–Ubiquitin Complexes. Values represent mean ± SD obtained from RMSF, RMSD, Rg, SASA, and intermolecular hydrogen bond analyses.

| **Analysis** | **Apo-PINK1** | **WT_PINK1-Ub** | **T313M_PINK1_Ub** | **L347P_PINK1-Ub** |
| --- | --- | --- | --- | --- |
| rmsf_mean | 1.0839 ± 0.3867 | 0.4521 ± 0.2223 | 1.0189 ± 0.4278 | 0.8416 ± 0.0593 |
| rmsf_std | 0.3964 ± 0.1578 | 0.2445 ± 0.0466 | 0.4129 ± 0.1504 | 0.3367 ± 0.0307 |
| rmsd_mean | 0.8102 ± 0.0000 | 0.9785 ± 0.1050 | 1.0700 ± 0.2185 | 1.0177 ± 0.0140 |
| rmsd_std | 0.1196 ± 0.0000 | 0.0838 ± 0.0112 | 0.1457 ± 0.0326 | 0.1102 ± 0.0243 |
| rg_mean | 3.0003 ± 0.1266 | 3.0923 ± 0.0843 | 3.1760 ± 0.0733 | 3.0199 ± 0.0528 |
| rg_std | 0.0815 ± 0.0303 | 0.0786 ± 0.0340 | 0.0637 ± 0.0119 | 0.0778 ± 0.0265 |
| sasa_mean | 318.3748 ± 5.5279 | 355.3442 ± 4.5139 | 356.9244 ± 1.6072 | 360.2930 ± 3.7158 |
| sasa_std | 7.9509 ± 0.5429 | 11.8733 ± 1.9025 | 10.9432 ± 3.1410 | 11.0537 ± 3.0878 |
| hbond_mean | N/A | 3.4606 ± 0.7426 | 3.7720 ± 1.4532 | 3.1206 ± 0.8755 |
| hbond_std | N/A | 2.2609 ± 0.1767 | 2.2455 ± 0.5374 | 2.3775 ± 0.3652 |

**Supplementary Table 7**

MM/PBSA binding free energies (ΔGbind) of wild-type and mutant protein–protein complexes calculated from three independent simulation replicates. Abbreviations: WT, wild type; SD, standard deviation. The values represent MM/PBSA binding free energies obtained from three independent molecular dynamics simulation replicates. More negative values indicate stronger predicted binding affinity.

| **System** | **Replicate 1** | **Replicate 2** | **Replicate 3** | **Mean ± SD (kcal/mol)** |
| --- | --- | --- | --- | --- |
| **WT** | -905.35 | -1874.49 | -1875.67 | -1551.84 ± 559.87 |
| **T313M** | -905.44 | -1872.71 | -1864.18 | -1547.44 ± 556.01 |
| **L347P** | -902.05 | -1873.02 | -1864.54 | -1546.54 ± 558.16 |
